# Marine protected areas for dive tourism

**DOI:** 10.1038/s41598-024-83664-1

**Published:** 2025-02-11

**Authors:** Reniel B. Cabral, Katherine D. Millage, Juan Mayorga, Tia Kordell, Mukta Kelkar, Alicia M. Caughman, Fabio Favoretto, Anna Schuhbauer, Octavio Aburto-Oropeza, Enric Sala, Darcy Bradley

**Affiliations:** 1 College of Science and Engineering, James Cook University, Townsville, QLD Australia; 2 Centre for Sustainable Tropical Fisheries and Aquaculture, James Cook University, Townsville, QLD Australia; 3 Environmental Markets Lab, University of California Santa Barbara, Santa Barbara, CA USA; 4 Pristine Seas, National Geographic Society, Washington, DC USA; 5 Bren School of Environmental Science and Management, University of California Santa Barbara, Santa Barbara, CA USA; 6 Marine Science Institute, University of California Santa Barbara, Santa Barbara, CA USA; 7 Centro Para la Biodiversidad Marina y Conservación, A.C., La Paz, Baja California Sur Mexico; 8 Scripps Institution of Oceanography, University of California San Diego, La Jolla, CA USA; 9 Institute for the Oceans and Fisheries, University of British Columbia, Vancouver, Canada

**Keywords:** Biodiversity, Marine biology, Sustainability

## Abstract

Marine and coastal tourism deliver economic benefits to coastal communities that far surpass those generated by fisheries, yet its potential contribution to global marine conservation remains underexamined. Marine Protected Areas (MPAs) help restore biodiversity and enhance nearby fisheries, but their direct tourism benefits are not well understood. Here, we estimate the global demand for recreational scuba dive tourism, map the distribution and protection status of all marine dive sites globally, and develop a bioeconomic model to estimate the revenue gains from upgrading unprotected dive sites to fully protected MPAs. We estimate that 33.1 million scuba dives occur annually in marine environments worldwide, with 70% taking place within MPAs. However, only 15% of these MPA-affiliated dive sites are highly or fully protected. We show that designating all unprotected recreational dive sites, representing less than 1% of the global ocean, as highly or fully protected MPAs would improve fish biomass and biodiversity while generating an additional US$2 billion per year in direct tourism revenue (not including economic multipliers). Importantly, 62% of marine diving currently occurs in developing countries, underscoring the potential for dive tourism to support both marine conservation and local livelihoods in regions where such benefits are most needed.

**Supplementary Information:**

The online version contains supplementary material available at 10.1038/s41598-024-83664-1.

## Introduction

Highly protected marine areas^1^ increase the biomass and diversity of marine life (and in particular, commercial species) and restore marine ecosystems within their borders^2–4^. The increase in marine life can in turn create premium tourism opportunities by attracting divers, snorkelers, whale watchers, and other recreational ocean users who travel to see abundant marine life they cannot see in overexploited areas^5^. However, most dive sites in the world are not protected from unsustainable fishing and other damaging activities, and thus cannot realize their full ecological and economic potential. Here we ask: What is the current geography, extent, and protection status of recreational scuba diving globally, and what would be the potential change in ecological and economic benefits if all dive sites globally were fully protected?

Recreational scuba diving is a multi-billion dollar industry that has experienced rapid growth since the 1970s. One of the largest global certification agencies for scuba divers, the Professional Association of Diving Instructors (PADI), issued more than 28 million dive certifications between 1968 and 2020, and certifies an additional 1 million new divers each year^6^. Scuba divers pay more to see more fish in the water^7,8^, and are willing to pay more for more biodiversity on their dives^7,9^—both of which increase with increasing levels of protection^2,10,11^. Additionally, the mere fact that a dive site is within an MPA increases divers’ willingness to pay for access—hereafter referred to as the “MPA name effect” — even before biological improvements have occurred^12,13^. We use these well-documented increases in divers’ willingness to pay to dive in areas designated as MPAs, with greater biomass, and greater species diversity to assess the untapped economic opportunity presented by expanding a global network of MPAs to include all dive sites globally.

For our analysis, we assembled a database of dive shops, sites, and prices from thousands of locations to estimate diving supply and demand curves at a 50 km x 50 km resolution globally. We estimate shifts in the demand for diving (i.e., the number of dives at a site) resulting from protection via (i) increases in fish biomass and diversity, and (ii) the MPA name effect using a spatially explicit model of biodiversity and biomass benefits of MPAs^14^ and a synthesis of diving-specific willingness to pay studies (Supplementary Figs. 1, 2). We report expected changes to total economic revenue and consumer surplus (i.e., diver satisfaction) that result from increased demand for diving associated with the implementation of a network of MPAs on existing dive sites (Supplementary Fig. 3). We then examine the potential to locally capture the additional value generated by new or enhanced (protection level) MPAs (Supplementary Fig. 4).

## Results

### Global patterns of diving

**Fig. 1:**
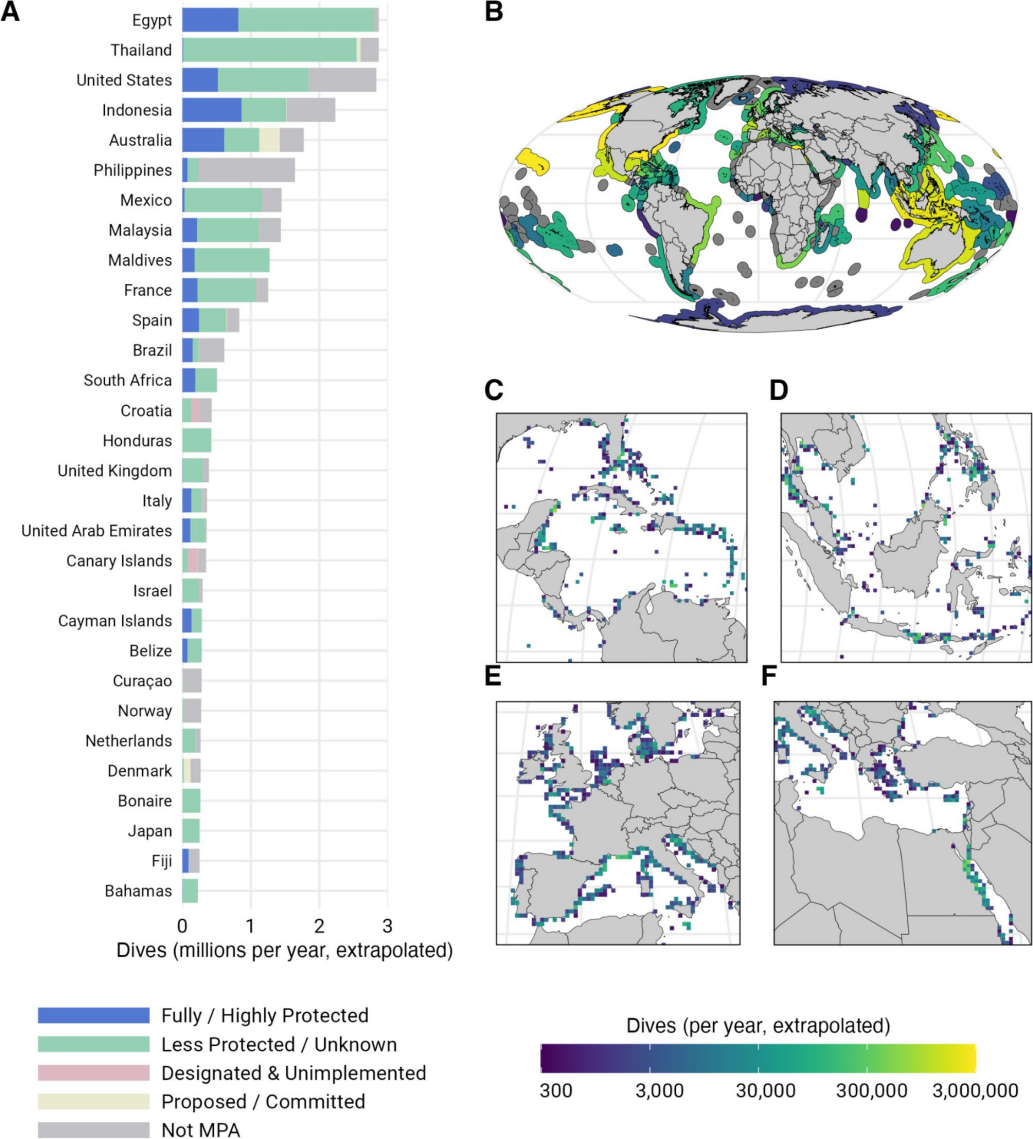
Current status of marine scuba diving worldwide. (**A**) Estimated number of marine dives made annually within each country or territory by protection status. Joint regime areas and contested areas are not included. (**B**) Estimated number of marine dives made annually by EEZ. (**C**) Close-up view of the Caribbean Sea and Central America. (**D**) Close-up view of the Coral Triangle in the Western Pacific Ocean. (**E**) Close-up view of Western Europe and the Mediterranean Sea. (**F**) Close-up view of Eastern Europe, the Mediterranean Sea, and the Red Sea. Panels (**C**–**F**) have the extrapolated number of marine dives made annually binned into 50 km x 50 km pixels. Base maps were generated using the R package rnaturalearth version 0.1.0^45^.

Though the recreational dive industry is estimated to be worth billions of dollars, global statistics on scuba diving are sparse due to the decentralization of certification agencies. We estimate 50.7 million scuba dives are made worldwide each year (lower bound = 26.2 million, upper bound = 82.7 million; see “Supplementary Information”), 65.3% (33.1 million) of which are made in the marine environment (lower bound = 17.1 million, upper bound = 54.0 million, Fig. 1A,B). More than half (51.69%) of all recreational dives in the ocean take place within the Exclusive Economic Zones (EEZs) of eight countries: Egypt, Thailand, the United States, Indonesia, Australia, the Philippines, Mexico, and Malaysia (Fig. 1A,B). Moreover, 62% of all marine diving occurs in developing countries.

Using a spatial grid with 50 km x 50 km resolution pixels, we estimate recreational scuba diving was present in 1.21% or 4.54 million km^2^ of the global ocean between 2010 and 2020 (Supplementary Fig. 11). A pixel is considered highly or fully protected if at least 50% of its area is in highly or fully protected MPAs. Less than 2% of the pixels with diving are highly or fully protected. Nonetheless, the proportion of protected marine dive sites increases as we increase the spatial resolution of our analysis because most MPAs are small and coastal^15^.

Using the central coordinates of 27,609 established marine dive sites worldwide (see “Supplementary Information”), we estimate that 67.35% are located within the boundaries of MPAs, but less than a quarter of those (15.48% of all dive sites) are within highly or fully protected areas (Supplementary Figs. 13, 14). An even greater fraction of all marine dives are made in MPAs: 70.15% of recorded dives between 2010 and 2020 were made at sites within MPAs, with 16.64% of all dives being made in highly or fully protected MPAs (Supplementary Figs. 16, 17).

The median price per dive is US$58.75 globally (Supplementary Figs. 9, 10; Supplementary Table 3), resulting in a total revenue of US$1.94 billion per year (lower bound = US$1 billion per year, upper bound = US$3.17 billion per year) from only the scuba diving activity that occurs in the marine environment (that is, the revenue generated at a dive center, not accounting for other costs such as transportation, accommodation, gastronomy, and services). The consumer surplus of marine scuba diving is US$2.67 billion per year (lower bound = US$1.38 billion per year, upper bound = US$4.36 billion per year), which is greater than the revenue of the dive industry from recreational scuba dive tourism.

### Changes resulting from MPAs

To model changes in the demand for diving (i.e., the number of dives at a site) due to the upgrade of dive sites’ protection status, we need to model changes in biomass and biodiversity due to MPA and estimate the value of the “MPA name effect.” We note that other factors could affect the demand and supply of diving but are difficult to predict and model, including political unrest, peace and order, social marketing, and the idiosyncrasies of social media.

We model biomass change of fish and invertebrates from MPAs by modifying the methods described in Sala et al.^14^. In particular, we explicitly parameterize the dispersal of adults and larvae using a random forest regression model informed by empirical home range and pelagic larval duration estimates collected from the literature^16^ (Supplementary Figs. 5, 6). We also assume that the spillover of biomass from protected areas to adjacent fished areas will all be captured by fishers, hence, any build-up of biomass from MPAs will only happen inside the MPAs.

**Fig. 2:**
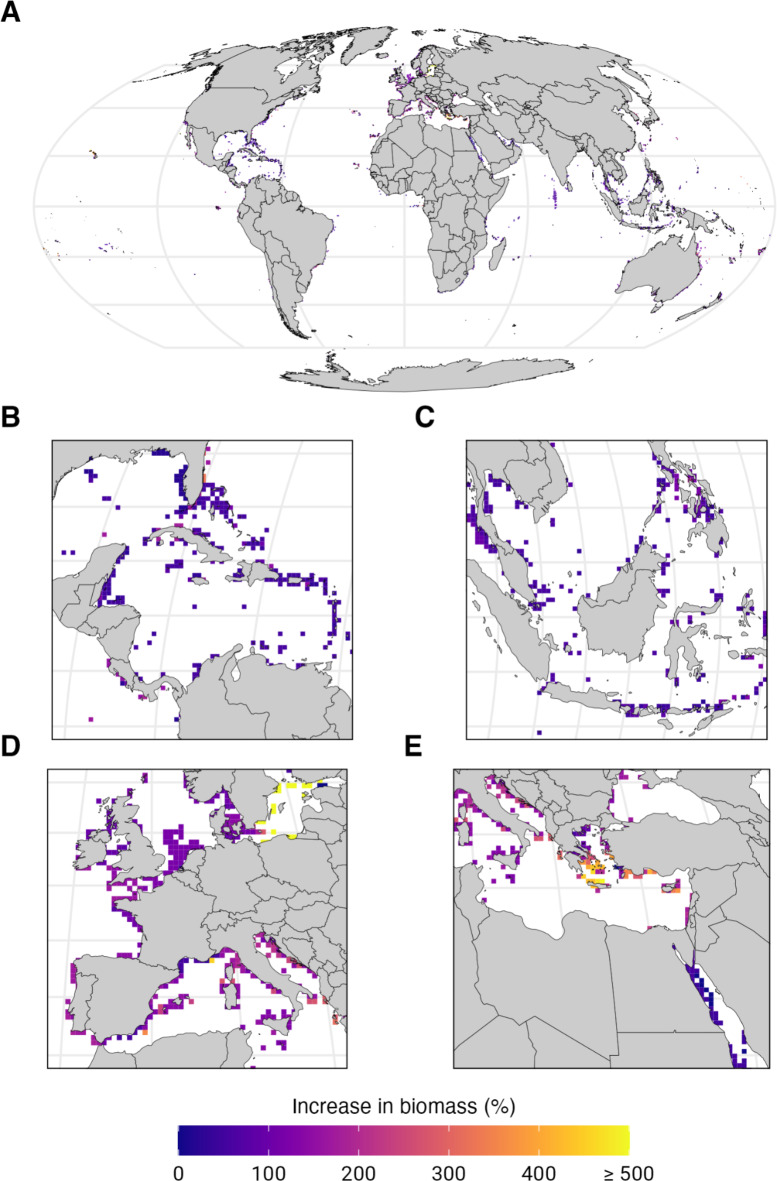
Predicted change in biomass resulting from the implementation of highly protected marine protected areas in ocean areas with recreational diving (% change relative to business as usual). (**A**) Global view binned into 50 km x 50 km pixels. (**B**) Close-up view of the Caribbean Sea and Central America. (**C**) Close-up view of the Coral Triangle in the Western Pacific Ocean. (**D**) Close-up view of Western Europe and the Mediterranean Sea. (**E**) Close-up view of Eastern Europe, the Mediterranean Sea, and the Red Sea. Base maps were generated using the R package rnaturalearth version 0.1.0^45^.

Our results show that upgrading the protection status of all unprotected dive site pixels to fully-protected MPAs can increase the biomass in those pixels by an average (weighted mean by fish stock’s carrying capacity) of 113% (± 133% s.d.) (Fig. 2). This is an underestimate relative to global meta-analyses finding on average 400–570% higher biomass in fully-protected MPAs relative to unprotected areas nearby^2,4^. Our conservative estimate is driven by fish populations with unreported and miscellaneous (not explicitly identified) catches, which are likely overfished and would benefit most from MPAs, but they have insufficient data to be included in our analysis. Specifically, biomass recovery estimates are likely underestimated in the Coral Triangle (Fig. 2), where illegal, unreported, and unregulated fishing remains a major concern^17^.

We follow the approach of Sala et al.^14^ to model biodiversity benefits from MPAs. Briefly, we use a biodiversity score to quantify biodiversity benefits from MPAs which is calculated as the weighted sum of the marginal gain in the persistence of marine species resulting from the removal of abatable impacts (i.e., fishing) relative to business as usual (i.e., no additional MPA). We consider the native ranges of 4,242 marine species^18^ that are directly or indirectly affected by fishing as reported by the International Union for Conservation of Nature (IUCN) or reported in global catch databases (see “Supplementary Information”). Placing the ~ 1% of ocean area with recreational scuba diving that is currently unprotected in highly protected MPAs can improve the global biodiversity score by 5% (or from a biodiversity score of 0.54 to 0.57, with a score of 1 indicating that all marine species are free from any threats). For reference, protecting the entire ocean would improve the biodiversity score by 39% (or from a biodiversity score of 0.54 to 0.76)^14^, because full protection of marine biodiversity can only be achieved by also addressing threats unabatable by MPAs such as nutrient pollution and global warming.

We estimate the additional demand for diving resulting from highly protected MPAs and their biological effects by synthesizing willingness to pay studies. Our synthesis shows that divers are willing to pay 4% more to dive in MPAs just because they are “protected” (“MPA name effect,” Supplementary Table 7), up to 84% more for biomass increases (Supplementary Table 8), and up to 82% more for biodiversity increases (Supplementary Table 9). We use these empirical estimates to parameterize the magnitude of the diver’s willingness to pay as a function of model-derived changes in biomass and biodiversity from MPAs, where willingness to pay is higher for bigger changes in biomass and biodiversity (see “Supplementary Information”).

**Fig. 3:**
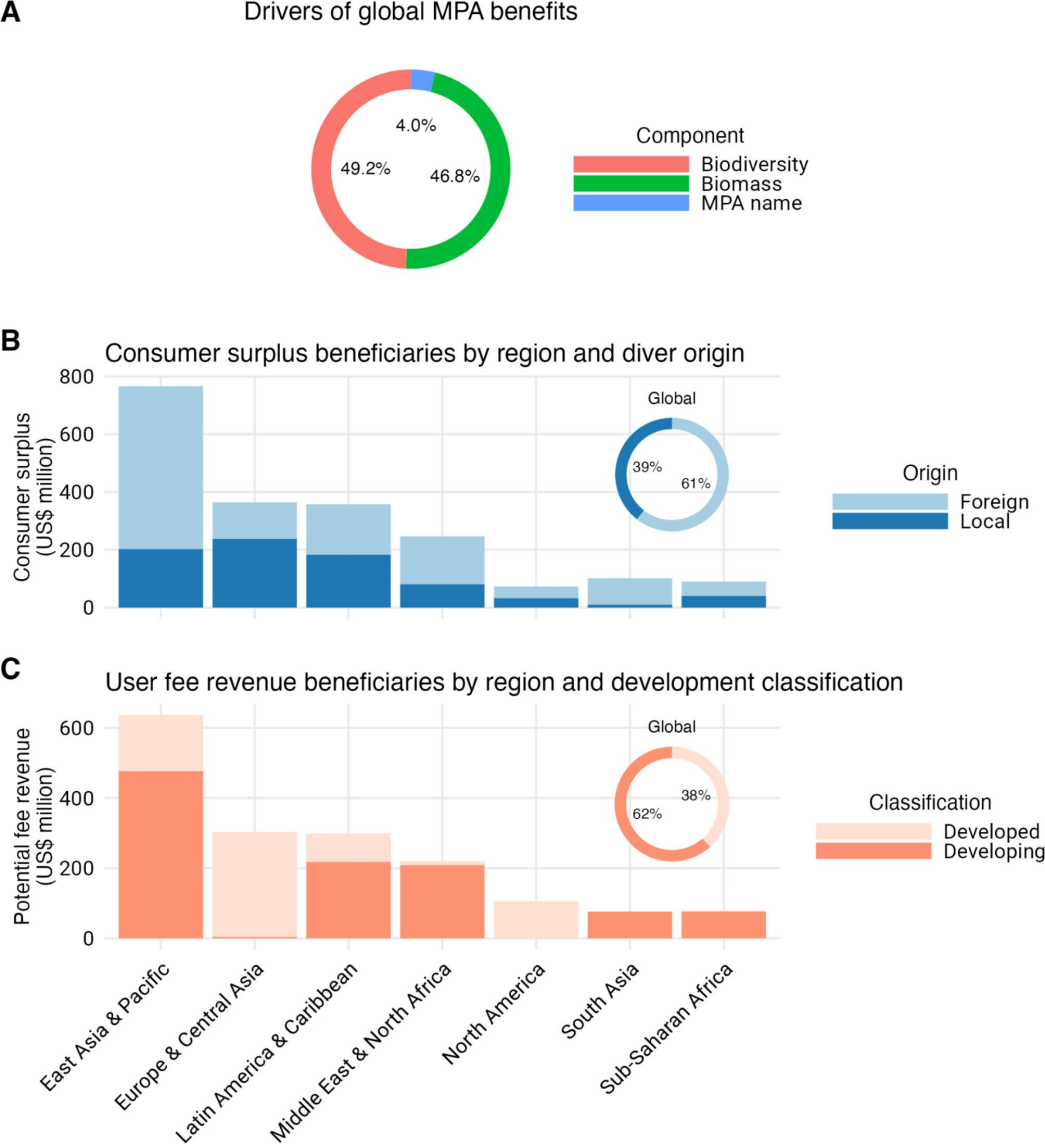
Drivers and beneficiaries of dive tourism benefits. (**A**) Contribution of different components to dive tourism benefits from marine protected areas (MPAs). (**B**) Distribution of consumer surplus benefits associated with MPA-driven dive tourism benefits by region and diver origin (local or foreign). (**C**) Distribution of dive fee revenue associated with dive tourism by region and country classification if a US$53 dive fee were to be implemented to capture MPA-driven benefits. This user fee was chosen because it results in no change in the number of dives, dive revenue, and consumer surplus pre- and post-MPA. The inset plots in (**B**,**C**) show the distribution of consumer surplus benefits and dive fee revenue by country globally.

Together with the expected increase in biodiversity, biomass, and the MPA name effect, implementing highly and fully protected MPAs in pixels with existing recreational diving would increase the demand for diving and the number of dives by 32% (or 10.49 million more dives per year), dive industry revenue by US$616 million per year, and consumer surplus by US$2 billion per year. 49% of these economic improvements are due to the increase in biodiversity, 47% to biomass increase, and 4% to the MPA name effect (Fig. 3A). We estimate that 61% of the total global consumer surplus from diving in newly implemented MPAs would be captured by foreign divers (based on current visitation rates by region, see “Supplementary Information”; Supplementary Fig. 20), with most of the consumer surplus from diving captured by foreign divers in almost all regions of the world (Fig. 3B). These consumer surpluses are produced mainly in dive sites in East Asia, Pacific (including Oceania), Europe, Latin America, and the Caribbean (Fig. 3B). While over 60% of new dives are demanded in developing countries, 67% of the consumer surplus in these countries would be captured by foreign recreational divers (Supplementary Fig. 23).

**Fig. 4:**
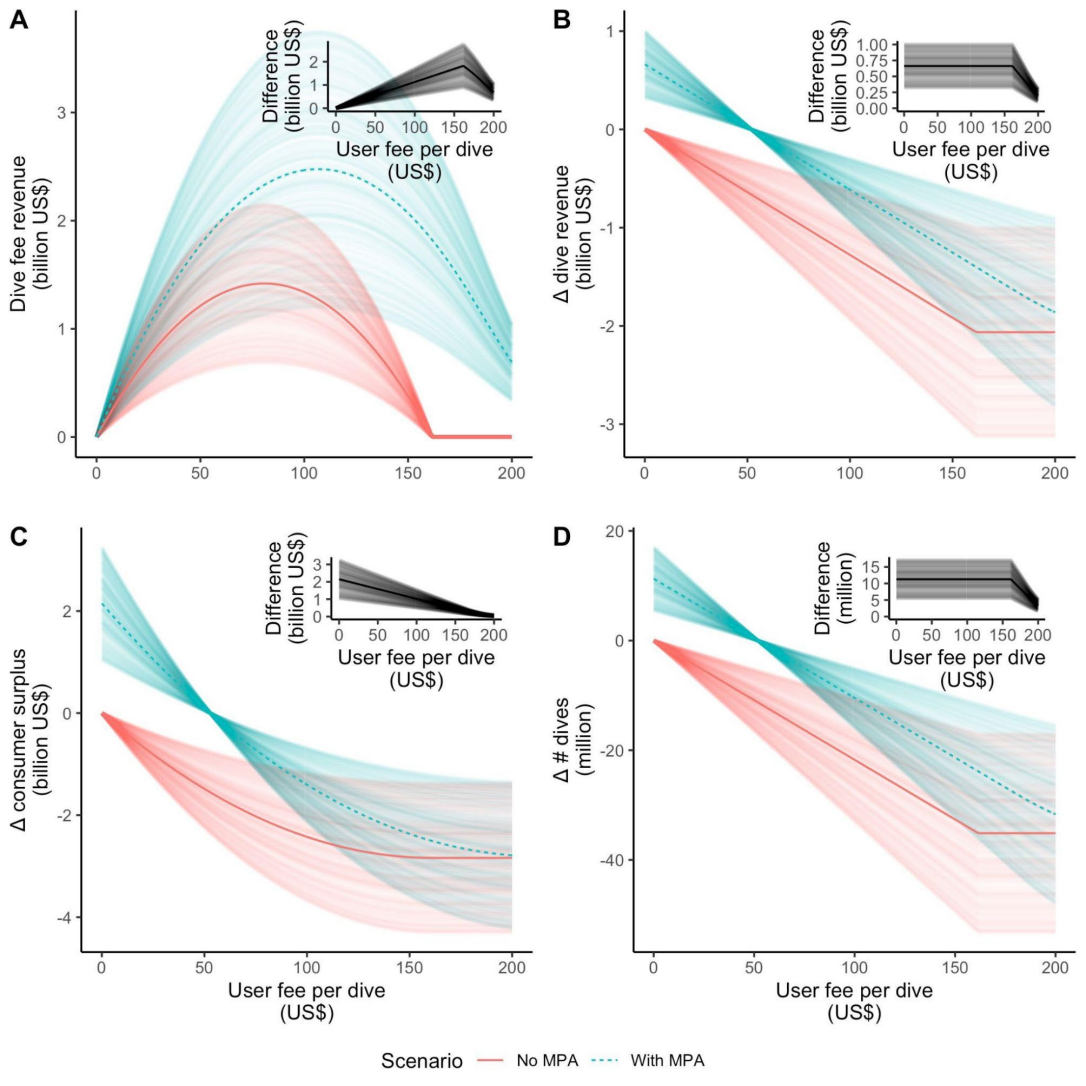
Economic effects of implementing dive fees in marine areas with and without fully or highly protected marine protected areas (MPAs). (**A**) Total revenue generated from dive fees at different levels of fees charged per dive, with and without MPAs. The other panels show the corresponding (**B**) changes in dive industry revenue, (**C**) changes in consumer surplus, and (**D**) changes in the number of dives. The multiple lines per scenario represent the results of 500 model runs that consider randomly assigned dive numbers drawn from our estimated range of global dive numbers. The insets show the value difference between the MPA and no-MPA scenarios per model run. Note that in all model runs, the MPA scenario always generates greater dive fee revenue, dive revenue to the industry, consumer surplus, and change in the number of dives compared to the no-MPA scenario.

If inadequately monitored, increasing the number of dives at a site could undermine positive MPA effects^19,20^. For example, direct anthropogenic impacts (i.e., divers causing damage to coral reefs) can negatively impact biodiversity^21^. We thus assessed the change in revenue associated with holding the number of dives at a site constant (e.g., by imposing a dive fee), thereby isolating revenue changes due to divers’ willingness to pay to access an MPA. When all dive sites are protected, we find that, on average, divers are willing to pay an additional US$53 per dive inside an MPA, because the individual diving experience is better due to the biological improvements and the perception of better diving within MPAs (Fig. 4). This increase in willingness to pay will result in increased dive activities in the absence of a dive fee. Implementing a dive fee equal to the additional willingness to pay of divers holds dive numbers constant pre- and post-MPA implementation, while generating US$1.74 billion in additional revenue (i.e., additional revenue from imposing an MPA use fee that does not affect the revenue of dive operators, Fig. 4). Majority (62%) of the potential dive fee revenue would go to the developing countries, with sizable benefits in East Asia and Pacific, Latin America and the Caribbean, and Middle East and North Africa (Fig. 3C).

## Opportunities for cost recovery

We assess how to optimize a dive fee to maximize economic benefits, and evaluate whether protection of dive sites brings in added revenue. With a dive fee set between US$0 and US$53 per dive to access highly and fully protected dive sites, it is possible to generate an additional US$1.97 billion per year from dive fee revenue while also increasing consumer surplus (diver satisfaction) and industry revenue (Fig. 4). This potential revenue is comparable to global estimates suggesting a running cost of US$0.3 – US$0.6 billion per year^22^ (with the higher bound cost estimate applicable to coastal areas where scuba dive tourism is concentrated) to US$1.2 billion per year^23^ to operate additional MPAs with a total size equivalent to 1% of the global ocean (the establishment and opportunity costs, if any, are not included in the cost estimates above). Making an equivalent change to the effective price per dive without establishing MPAs would reduce consumer surplus, the total number of dives, and consequently dive industry revenue (Fig. 4B, C).

## Discussion

Our results strongly suggest that upgrading the protection status of all recreational scuba dive sites can generate substantial economic benefits, with added revenue that could be used to support local livelihoods and offset MPA establishment and management costs, particularly in developing countries.

Marine and coastal tourism is projected to be the top ocean industry by 2030 when it will account for 26% of the ocean-based economy^24^. The economies of many developing and least-developed countries, where overfishing is common, will continue to rely heavily on diving tourism^25^ which is touted as an alternative or supplement to fishing as a source of income^26^. Our findings that most of the diving occurs in developing countries provide opportunities for MPAs to improve the local economy and well-being of local communities in developing countries. High-priority developing countries where more MPAs linked to dive tourism can generate substantial economic benefits are countries with high demand for dive tourism such as Thailand, Indonesia, and the Philippines. However, it is common that the benefits from dive tourism do not accrue locally (because dive operations may be foreign-owned and employ non-locals)^27–29^. Applying well-designed user fees in tandem with well-designed and effectively managed MPAs provides opportunities for multiple-win outcomes. Also unaccounted for, improved demand for tourism generates indirect economic benefits (also known as the multiplier effect or positive externalities from dive tourism) through increased demand for transportation, accommodation, gastronomy, and other services. It will thus be important to design interventions to locally capture the dive tourism benefits associated with new MPAs.

Examples of interventions that help local communities capture the benefits from MPA dive tourism include communities imposing taxes on businesses that benefit from MPAs, mandating that locals be prioritized for tourism employment opportunities, and collecting user fees with collection revenue that flows back to the local community. Perhaps a combination of the interventions identified above can be appropriate to ensure that economic benefits from MPA dive tourism stay in local communities and Island nations (versus ending up with big corporations). While user fees can be set at an amount that results in no change in dive numbers pre-MPA, a hybrid approach allowing increased tourism and charging some user fees is more likely in many coastal communities. The user fee amount can be tuned according to community goals. Setting a lower dive fee allows for increased tourism, benefiting areas requiring more tourism. Setting a high dive fee limits tourism demand, which is ideal for areas already experiencing overcrowding.

We recognize that there may be costs to fishers, at least in the short term, resulting from upgrading the protection status of dive sites. It is essential, then, that some of the benefits generated from MPA dive tourism are distributed in such a way that it compensates individuals negatively impacted by such an upgrade. For example, dive centers and fishing communities could partner in a joint venture where fishers obtain a share of the tourism profits proportional to their investment in the business (e.g., forgone fisheries profit)^30^. Communities will also need to carefully consider both the positive and negative consequences of increased tourism from MPAs and design the appropriate user fees and revenue sharing mechanism that generate net positive outcomes and fully offset the costs of fisher displacement and other potential societal disruptions.

There are key assumptions made in our analyses that merit attention: (1) We conservatively assume that increases in tourism demand from dive tourism MPAs will only occur in areas where dive tourism already exists. We recognize that MPAs can also stimulate the growth of the dive tourism industry in areas currently not frequented by tourists, but also recognize that some areas require infrastructure investments for tourism to happen. (2) We assume that the spillover of adult biomass and larval subsidies from MPAs to fished areas will be fully captured by fishers. As a result, our estimated tourism benefits are likely conservative given that an improvement in biomass and diversity in adjacent non-MPA areas may also benefit marine tourism in those areas and that the MPA name effect may similarly extend beyond the MPA border. (3) The economic benefits of MPA tourism reported here only include scuba dive tourism and underestimate the economic potential of MPAs, as other marine-based tourism activities positively interact with MPAs such as snorkeling and wildlife viewing tours^30^. (4) Another reason why divers may prefer to dive in MPAs, in addition to the MPA name effect considered here, is fish behavior. Fish wariness to divers is less inside MPAs^31^, creating a more enjoyable diving experience relative to an alternative non-protected dive site with comparable biodiversity and fish biomass. (5) It is possible that divers would select alternative dive sites as a response to changes in diving prices instead of increasing their diving frequency. Our model scenario that holds the number of dives constant using a dive fee as a mechanism solves the potential substitution effect among dive sites.

The tourism benefits we report here are unique to highly/fully protected MPAs, which produce the greatest improvements in marine life within their borders. On average, highly/fully protected MPAs experience increases of 21% in the number of species, 28% in the size of the organism, 166% in density, and 446% in biomass relative to unprotected areas nearby^4^. Significant increases in fish biomass, abundance, and diversity can be detected within 1–3 years of full protection^32^. Because the demand for diving is positively related to fish biomass and species diversity, we can strategically design a global network of highly/fully protected MPAs that improves dive tourism and captures the economic benefits from the rapid biological improvements within MPAs. However, only 8% of the global ocean is in MPA, with just 3% of the area fully or highly protected^15^. This extent of protection falls short of the global commitment to place 30% of the global ocean in effective protection and management by 2030^33^. The alignment of economic benefits with biodiversity conservation can help motivate more protection to help achieve this global target, restore marine life, support jobs, and generate increased economic benefits for coastal communities.

## Methods

### Quantifying changes to dive tourism benefits

We represent the number of dives demanded in non-MPA pixel *i* (${Q_{d,{i_0}}}$) as a function of the price per dive (${P_i}$) as:

1 $${Q_{d,{i_0}}}=a - b{P_i}$$

where *a* and *b* are constants and represent the number of dives that would be demanded when the price of diving is zero (i.e., ${P_i}=0$) and the slope of the demand curve, respectively. We estimate *a* and *b* by collecting spatially explicit diving data globally and estimate the current number of dives demanded per non-MPA pixel ($Q_{{{i_0}}}^{*}$), price per dive $(P_{{{i_0}}}^{*})$ information from thousands of dive sites globally (see Supplementary Information Sect. 2.4.1), and assumed that the scuba diving market is currently in equilibrium (i.e., $Q_{{{i_0}}}^{*}=a - bP_{{{i_0}}}^{*}$).

Our empirical analysis indicates that the price per dive is nearly constant across countries and regions (see Supplementary Information Sect. 2.4.1), implying that the supply curve is horizontal.

We quantified changes in dive revenue, producer surplus, and consumer surplus resulting from dive site protection upgrades and the collection of dive fees. Producer surplus is zero for a horizontal supply curve.

The change in the number of dives ($\Delta {Q_{{i_{mpa/fee}}}}$) when a dive fee (*F*_*i*_) or an MPA is applied at pixel *i* is:

2 $$\Delta {Q_{{i_{mpa/fee}}}}=\frac{{Q_{{{i_0}}}^{*}\left( {\Sigma WT{P_{mpa}} - {F_i}} \right)}}{{{C_{{i_0}}} - P_{{{i_0}}}^{*}}}.$$

where ${C_{{i_0}}}$ is the choke price or the price at which no tourist will pay to dive in non-MPA dive pixel *i*. Although the price per dive is horizontally flat on average, dive price variations around the mean exist. We assumed that the upper 99th percentile of our empirically derived dive price data constitutes our choke price. The change in the willingness to pay (WTP) of divers to dive in pixel *i* caused by the protection upgrades is denoted as $\Sigma WT{P_{mpa}}$, where the summation represents the combined effects of increases in fish biomass and biodiversity and the MPA name effect on WTP.

The change in the revenue of the dive industry from pixel *i* due to the implementation of a dive fee and/or MPA is:

3 $$\Delta DiveRevenu{e_i}=P_{{{i_0}}}^{*}\Delta {Q_{{i_{mpa/fee}}}}.$$

The dive fee revenue generated from implementing a dive fee in pixel *i* is:

4 $$FeeRevenu{e_i}={F_i}\left( {Q_{{{i_0}}}^{*}+\Delta {Q_{{i_{mpa/fee}}}}} \right).$$

The change in consumer surplus at site *i* due to the implementation of a dive fee and/or MPA is:

5 $$\Delta C\;Surplu{s_i}=0.5\frac{{{{\left( {Q_{{{i_0}}}^{*}+\Delta {Q_{{i_{mpa/fee}}}}} \right)}^2}\left( {{C_{i0}} - P_{{{i_0}}}^{*}} \right)}}{{Q_{{{i_0}}}^{*}}} - 0.5Q_{{{i_0}}}^{*}\left( {{C_{{i_0}}} - P_{{{i_0}}}^{*}} \right).$$

## Modeling biomass accrual and spillover in response to protection upgrades

We describe the biomass of stock *x* in dive MPA pixel *i* (i.e., *i* = MPA) at time *t + 1* following dive site protection upgrades as:

6 $$\begin{aligned} {B_{x,{i_{mpa}},t+1}} & ={s_{x,i \to i}}{B_{x,i,t}}+\sum\limits_{{j \ne i}} {{s_{x,j \to i}}{B_{x,j,t}}} +{\rho _{x,i \to i}}{r_x}{B_{x,i,t}}\left( {1 - \frac{{{B_{x,i,t}}}}{{{K_{x,i}}}}} \right) \\ & +\sum\limits_{{j \ne i}} {{\rho _{x,j \to i}}{r_x}{B_{x,i,t}}} \left( {1 - \frac{{{B_{x,i,t}}}}{{{K_{x,i}}}}} \right) \\ \end{aligned}$$

where ${s_{x,i \to i}}{B_{x,i,t}}$ *i*s the portion of adult biomass of stock *x* from pixel *i* that stays in pixel *i*, $\sum\nolimits_{{j \ne i}} {{s_{x,j \to i}}{B_{x,j,t}}}$ is the sum of all biomass of stock *x* from other pixels that move to pixel *i*, ${r_x}$ is the population growth rate, ${K_{x,i}}$ is the carrying capacity of stock x in pixel *i*, and ${\rho _{x,i \to i}}$ is the proportion of viable larvae of stock *x* produced in pixel *i* that settles in pixel *i*.

We used the species list reported in Sala et al.^14^ in our model. We performed an extensive literature review and built machine learning models to estimate the pelagic larval duration (PLD) and home range parameters information for as many of the 811 species (1,150 commercially relevant marine stocks) reported in Sala et al.^14^ as possible (see Supplementary Information Sect. 2.1, 2.2). We considered only those stocks whose geographic range intersects with any of the dive sites and with complete biological parameters. Our final database includes 813 commercially relevant fish stocks (representing 599 species), comprising 74% of the total carrying capacity from Sala et al.^14^.

Using data from Sala et al.^14^, we calculated the equilibrium biomass per stock at each pixel, assuming a business-as-usual scenario where there is no change in the protection status of all dive sites, by simultaneously running the biomass equation for all MPA pixels worldwide for 100 time-step iterations. We then calculated the equilibrium biomass for the scenario where all unprotected dive sites are upgraded into fully protected MPAs (i.e., no fishing inside MPAs). When the protection status of dive pixel *i* is upgraded, we assume that the biomass density in the surrounding fishing areas will be unchanged. This assumption implies that fishers will capture all adult biomass spillover and larval subsidy generated by the MPA.

We derived the stock geographic ranges from Aquamaps predicted species distribution maps^18^. Aquamaps produced a species-specific probability of occurrence maps with values ranging from 0 to 1, indicating the likelihood of the species being found in a specific area of the ocean. We apply a threshold of 0.5 to the Aquamaps predicted species distribution maps to generate the geographic range of stocks (i.e., the probability of occurrence must be 0.5 or above in pixel *i* for that pixel to be considered part of the stock range). The growth rate (${r_x}$) and carrying capacity (${K_x}$) for each stock are derived from Sala et al.^14^. We assume the carrying capacity for each stock is homogeneously distributed across the entire geographic range of the stock (i.e., ${K_{x,i}}={K_{x,j}}$). We take these simplifying assumptions regarding species ranges and carrying capacity distributions because there is no empirical information on stock distributions for most stocks considered in our model. In reality, species ranges may extend beyond the 0.5 probability of occurrence used here, and the distribution of carrying capacities per stock will vary in space.

### Parameterizing adult mobility

In parameterizing the adult mobility (${s_x}$), we assume that the home range (in km^2^) represents the area of a circle. So, the biomass at pixel *i* can only redistribute to other pixels within the radius $\zeta =\sqrt {homerange/\pi }$. Home range values for a total of 667 out of the 811 commercially relevant marine species (Supplementary Fig. 5) from Sala et al.^14 ^were predicted by a random forest regression model^16^. The model predicted home range values based on the intrinsic growth rate, carrying capacity, species length, species trophic level, movement keyword, and geographic range size. Empirical home range values for training the random forest were collected via literature review, which used a variety of field and analytic techniques for home range estimation. A total of 221 empirical home range values were collected, a number of which describe the same species. Life history information was obtained from Fishbase^34^. Geometric means were calculated for species with home range values from multiple studies, resulting in 67 unique species with observed home ranges and all required model data (i.e., life history and geographic range data). We give the empirically derived home range data priority when available and then use the predicted home range data for species with no empirical information.

### Parameterizing larval distribution

We represented the distribution of larvae produced at a specific site to other sites by a Gaussian dispersal kernel. The spread of the kernel is driven by the amount of time spent by larvae in the pelagic (i.e., stock-specific pelagic larval duration, $PL{D_x}$). We assumed that the larvae spread symmetrically from the source (i.e., we are neglecting the drift component of the larval dispersal). The proportion of larvae of stock *x* from pixel *j* that settles in pixel *i* is given by:

7 $${\rho _{x,j \to i}}=\frac{1}{{2\pi \sigma _{{larvae,x}}^{2}}}{e^{ - \frac{{d_{{j \to i}}^{2}}}{{2\sigma _{{larvae,x}}^{2}}}}}$$

where ${d_{j \to i}}$ is the distance from pixel *j* to pixel *i* and ${\sigma _{larvae,x}}$ is the spread of the larval dispersal kernel of stock *x*. The relationship between ${\sigma _{larvae,x}}$ and the pelagic larval dispersal of stock *x* ($PL{D_x}$) has been empirically derived by Siegel et al.^35^ and is given by:

8 $${\sigma _{larvae,x}}=1.33\sqrt {\frac{\pi }{2}} PLD_{x}^{{1.3}}$$

We used a similar random forest regression approach (as that used to estimate home range) to estimate PLD in days using empirical data for 91 species. PLD values were averaged for species with multiple empirical PLD values, and values for Elasmobranchs were removed, as they do not have pelagic larval stages. PLD values in days for 610 out of 811 of the commercially relevant species from Sala et al.^14^ were then predicted using intrinsic growth rate, carrying capacity, species length, trophic level, and movement keywords (see “Supplementary Information”).

### Modeling biodiversity effects in response to protection upgrades

We adapted Sala et al.’s^14^ approach to model the biodiversity benefits that could be gained for each species from additional protection conferred by dive tourism MPAs. Biodiversity benefits are defined as the weighted sum of the marginal gain in the persistence of marine species resulting from the removal of abatable impacts relative to business as usual. We consider the native ranges of 4,242 marine species^18^ that are directly or indirectly affected by fishing as reported by the International Union for Conservation of Nature (IUCN) or reported in global catch databases^36,37^.

Sala et al.^14^ weighted species as a function of their extinction risk, functional distinctiveness, and evolutionary distinctiveness such that not all species would contribute equally to the biodiversity benefit. This method resulted in species in the subclass Elasmobranchii (sharks, rays, skates, and sawfish; 34.0%) being given the greatest aggregate weight, followed by species in the class Actinopterygii (ray-finned fishes; 27.3%), species in the subphylum Anthozoa (sea anemones, stony corals, and soft corals; 16.5%), birds (5.2%), mammals (5.0%), species in the class Malacostraca (crabs, lobsters, crayfish, shrimp, krill, prawns, woodlice, amphipods, mantis shrimp; 3.8%), and then cephalopods (squid, octopus, cuttlefish, and nautilus; 2.1%). All remaining species accounted for the remaining 6.1% (Supplementary Table S2).

As part of the general information collected for each of the dive operators represented in our global database of diving prices, we recorded mentions of specific species (or groups of species) advertised as those likely to be seen while diving with that operator and compared with the species weightings used above. In total, we recorded 1,800 mentions of different species or groups of species that we were able to match to a taxonomic class from the websites of 240 different dive operators. We then calculated the percentage of total mentions corresponding to different taxonomic groups (Supplementary Table S2). The majority of mentions were of species (or groups of species) of the class Actinopterygii and subclass Elasmobranchii. Together these two taxonomic groups accounted for 67.6% of all mentions recorded from the dive operator websites (compared to 61.3% of the aggregate species weight).

### Creating a globally representative database of diving prices

We utilized a global Google Maps-derived database of dive operators (*N*= 11,132) as a sampling frame from which to extract data directly from operators on their prices^38^. We filtered the global operator database created by Schuhbauer et al.^38^ to only include operators for which an active website had been obtained (*N* = 9,909) and then employed a geographically stratified sampling method to partition the filtered database into five strata defined based on region. We sampled 1,021 distinct operators, of which, 948 had a valid URL, 902 were deemed to be comprehensible, 762 were found to pertain to dive-related businesses, and 534 businesses were found to offer marine diving classes or services (Supplementary Fig. 8).

We spatially allocated the price per dive based on the geographic coordinates of the operators and binned into 50 km x 50 km pixels. The median price per dive was calculated for each pixel, for each country, for each region, and globally. We considered three scenarios for spatially allocating dive prices to different areas of the ocean: (1) The global median dive price is uniformly applied to all ocean pixels (reference case scenario). (2) The median dive price per country is uniformly applied to all ocean pixels falling under that country’s jurisdiction. (3) Dive prices are interpolated for all pixels based on the median price per dive per pixel (see sensitivity analysis in “Supplementary Information”).

We also gathered data on dive prices from published and gray literature using Web of Science and Google Scholar for comparison with the database on global diving prices we curated. Prices of scuba diving activities reported in the literature are summarized in Supplementary Table 4.

### Suitability for recreational dive tourism

We used a crowdsourced database of logged dives provided by Diveboard to identify where recreational diving currently occurs. We kept all dives with a valid date that occurred at or shallower than 140 ft (or 43 m) to provide a slight buffer for the “industry standard” 130 ft (40 m) limit defined for recreational diving, knowing that divers occasionally exceed this limit for short periods of time. We then identified valid dive sites from the Diveboard database by removing those where the user failed to specify a location or specified invalid coordinates. Finally, we matched the database of valid recreational dives back to their corresponding dive sites to determine whether or not each 50 km x 50 km ocean pixel is suitable for recreational diving. For our model, the presence of any dive site in a pixel with a logged recreational dive between 2010 and 2020 resulted in the pixel being deemed to be suitable for recreational diving in this analysis.

### Protection status of dive sites

We determined the protection status for the marine dive sites using the boundaries of global MPAs and other effective area-based conservation measures (OECMs) from the MPAtlas database^15^. At the time of writing, MPAtlast was the best available data source for strict protection, but we recognize that many MPAs and OECMs still need to be accounted for and assessed. Since MPAtlas no longer classify protected areas as “Fully/Highly Protected” or “Less Protected/Unknown”—used by Sala et al.^14^—we recreated these classifications for the most recent version of the MPAtlas utilizing their original methodology. In cases where a dive site is covered by multiple protected area designations, we retain the protected area designation conferring the highest protection status (“Fully/Highly Protected,” “Less Protected/Unknown,” “Designated & Unimplemented,” “Proposed/Committed”) or the oldest designation (if all designations have confer equal protection).

### Estimating the total number of recreational scuba dives made annually

The Dive Equipment and Marketing Association (DEMA) reports in the “2022 Diving Fast Facts” that there are as many as 6 million active scuba divers worldwide and there were 2.59 million active divers in the United States in 2020 (estimates for the number of active divers in the United States in previous years have ranged between 2.7 million and 3.5 million)^39^. Kieran^40^ also estimates the global number of divers. In his extrapolation, he makes reference to the estimate of 2.6 million active divers in the United States from the 2021 Sports and Fitness Industry Association (SFIA) report, but also references a Recreational Scuba Training Council (RSTC) Europe report, which claims that the number of active divers in Europe is between 3 and 4 million. Kieran uses this figure to argue that the global estimate of 6 million active divers stated by DEMA is likely too low. Citing other information suggesting that the sizes of the American and European markets are likely roughly equivalent, they go on to argue that it would be reasonable to assume that the rest of the world comprises an approximately equal market share, putting the total number of active divers worldwide in the ballpark of 9 million. We believe this to be a realistic assumption, as other sources^41,42^ suggest the total number of active divers worldwide to be higher than 6 million.

The definition of an “active” scuba diver is simply anyone who has participated in scuba diving, but the SFIA 2021 Topline Participation Report^43^—from which DEMA sourced the statistic on the number of active American divers—breaks down the information further by defining two categories of participants: those who participate in scuba diving one to seven times per year (“casual” divers) and those who participate eight or more times per year (“core” divers). We assume that the casual diver makes, on average, four dives per year and the core diver makes, on average, 10 dives per year. We then extrapolate these values globally, assuming that active divers outside of the United States have the same participation rates. Using the estimated 9 million active divers worldwide and data from 43,023 dive site locations to determine the proportion of dives in the marine environment (Supplementary Information Sect. 2.5.1), we estimate that between 26.2 and 82.7 million dives, with 50.7 million as a central value, are made per year, with 65.3% (33.1 million) of which are made in the marine environment (lower bound = 17.1 million, upper bound = 54.0 million). We use the 33.1 million total marine dives in our model as the base case scenario and perform model sensitivity with the lower and upper bounds (see Supplementary Information Sect. 3.1).

### Synthesis of willingness-to-pay studies

We searched and synthesized willingness-to-pay (WTP) studies using the Web of Science and Connected Papers to parameterize three factors known to shift the demand curve, namely: MPA name effect, changes in fish biomass (or abundance), and changes in species diversity (see “Supplementary Information”). Improvements in habitat quality due to MPAs could also lead to higher WTP, but we did not include this factor given our lack of general understanding about the magnitude of benefits MPAs provide to habitat maintenance and recovery. Furthermore, it has been shown that dive tourists respond most to changes in fish attributes (e.g., variety of fishes, abundance of fishes, big fishes) than habitat-based attributes^5^.

### Sensitivity analysis

We evaluated the sensitivity of our reported maximum revenue that can be generated from combining dive fee and MPA to alternative parameter values. We run our model using the lower and upper bound of our estimated yearly global number of marine dives (i.e., 17.1 million and 54 million dives per year, respectively), alternative assumptions about the price per dive parameterization, and alternative weighting of biodiversity scores (see “Supplementary Information”).

## Electronic supplementary material

Below is the link to the electronic supplementary material.

Supplementary Material 1

## Data Availability

The data and codes that support this study are available at 10.5281/zenodo.8034977^44^.

## References

[1] Grorud-Colvert, K. et al. The MPA guide: A framework to achieve global goals for the ocean. *Science* **373**, eabf0861 (2021). 34516798 10.1126/science.abf0861

[2] Sala, E. & Giakoumi, S. No-take marine reserves are the most effective protected areas in the ocean. *ICES J. Mar. Sci.* **75**, 1166–1168 (2018).

[3] Edgar, G. J. et al. Global conservation outcomes depend on marine protected areas with five key features. *Nature* **506**, 216–220 (2014). 24499817 10.1038/nature13022

[4] Lester, S. E. et al. Biological effects within no-take marine reserves: A global synthesis. *Mar. Ecol. Prog Ser.* **384**, 33–46 (2009).

[5] Williams, I. D. & Polunin, N. V. C. Differences between protected and unprotected reefs of the western Caribbean in attributes preferred by dive tourists. *Environ. Conserv.* **27**, 382–391 (2000).

[6] PADI. 2021 Worldwide Corporate Statistics: Data for 2015–2020 (2021).

[7] Grafeld, S. et al. Divers’ willingness to pay for improved coral reef conditions in Guam: An untapped source of funding for management and conservation? *Ecol. Econ.* **128**, 202–213 (2016).

[8] Gill, D. A., Schuhmann, P. W. & Oxenford, H. A. Recreational diver preferences for reef fish attributes: Economic implications of future change. *Ecol. Econ.* **111**, 48–57 (2015).

[9] Cazabon-Mannette, M., Schuhmann, P. W., Hailey, A. & Horrocks, J. Estimates of the non-market value of sea turtles in Tobago using stated preference techniques. *J. Environ. Manag.* **192**, 281–291 (2017). 10.1016/j.jenvman.2017.01.072 28183028

[10] Turnbull, J. W., Johnston, E. L. & Clark, G. F. Evaluating the social and ecological effectiveness of partially protected marine areas. *Conserv. Biol.* **35**, 921–932 (2021). 33448038 10.1111/cobi.13677 PMC8248084

[11] Sciberras, M., Jenkins, S. R., Kaiser, M. J., Hawkins, S. J. & Pullin, A. S. Evaluating the biological effectiveness of fully and partially protected marine areas. *Environ. Evid.* **2**, 4 (2013).

[12] Sala, E. et al. A general business model for marine reserves. *PLoS ONE* **8**, e58799 (2013). 23573192 10.1371/journal.pone.0058799 PMC3616030

[13] Viana, D. F., Halpern, B. S. & Gaines, S. D. Accounting for tourism benefits in marine reserve design. *PLoS ONE* **12**, e0190187 (2017). 29267364 10.1371/journal.pone.0190187 PMC5739484

[14] Sala, E. et al. Protecting the global ocean for biodiversity, food and climate. *Nature* **592**, 397–402 (2021). 33731930 10.1038/s41586-021-03371-z

[15] Marine Conservation Institute. Atlas of Marine Protection (2020); http://mpatlas.org.

[16] Bradley, D. et al. Marine Fish Movement: Home range sizes for commercially relevant species. *Sci. Data* **11**, 865 (2024). 39127749 10.1038/s41597-024-03728-9 PMC11316759

[17] Cabral, R. B. et al. Rapid and lasting gains from solving illegal fishing. *Nat. Ecol. Evol.* **2**, 650–658 (2018). 29572526 10.1038/s41559-018-0499-1

[18] Kaschner, K. et al. AquaMaps: Predicted range maps for aquatic species. World wide web electronic publication, Version 08/2016c (2016). www.aquamaps.org.

[19] Barker, N. H. L. & Roberts, C. M. Scuba diver behaviour and the management of diving impacts on coral reefs. *Biol. Conserv.* **120**, 481–489 (2004).

[20] Hasler, H. & Ott, J. A. Diving down the reefs? Intensive diving tourism threatens the reefs of the northern Red Sea. *Mar. Pollut Bull.* **56**, 1788–1794 (2008). 18701118 10.1016/j.marpolbul.2008.06.002

[21] Hawkins, J. P. & Roberts, C. M. Effects of recreational SCUBA diving on fore-reef slope communities of coral reefs. *Biol. Conserv.* **62**, 171–178 (1992).

[22] Balmford, A., Gravestock, P., Hockley, N., McClean, C. J. & Roberts, C. M. The worldwide costs of marine protected areas. *Proc. Natl. Acad. Sci.* **101**, 9694–9697 (2004). 15205483 10.1073/pnas.0403239101 PMC470737

[23] Cullis-Suzuki, S. & Pauly, D. Marine protected area costs as beneficial fisheries subsidies: A global evaluation. *Coast Manag.* **38**, 113–121 (2010).

[24] OECD. *The Ocean Economy in 2030* (OECD Publishing, 2016).

[25] Spalding, M. et al. Mapping the global value and distribution of coral reef tourism. *Mar. Policy* **82**, 104–113 (2017).

[26] Gier, L., Christie, P. & Amolo, R. Community perceptions of scuba dive tourism development in Bien Unido, Bohol Island, Philippines. *J. Coast Conserv.* **21**, 153–166 (2017).

[27] Pascoe, S. et al. Estimating the potential impact of entry fees for marine parks on dive tourism in South East Asia. *Mar. Policy* **47**, 147–152 (2014).

[28] Daldeniz, B. & Hampton, M. P. Dive tourism and local communities: Active participation or subject to impacts? Case studies from Malaysia. *Int. J. Tour Res.* **15**, 507–520 (2013).

[29] Gerungan, A. & Chia, K. W. Scuba diving operators’ perspective of scuba diving tourism business in Nusa Penida, Indonesia. *J. Outdoor. Recreat Tour.* **31**, 100328 (2020).

[30] Sala, E. et al. Fish banks: An economic model to scale marine conservation. *Mar. Policy* **73**, 154–161 (2016).

[31] Januchowski-Hartley, F. A., Graham, N. A. J., Cinner, J. E. & Russ, G. R. Local fishing influences coral reef fish behavior inside protected areas of the Indo-Pacific. *Biol. Conserv.* **182**, 8–12 (2015).

[32] Halpern, B. S. & Warner, R. R. Marine reserves have rapid and lasting effects. *Ecol. Lett.* **5**, 361–366 (2002).

[33] Convention on Biological Diversity. *First draft of the post-2020 global biodiversity framework* (2021).

[34] Pauly, D. & Froese, R. *FishBase* (World Wide Web Electronic Publication, 2022).

[35] Siegel, D. A., Kinlan, B. P., Gaylord, B. & Gaines, S. D. Lagrangian descriptions of marine larval dispersion. *Mar. Ecol. Prog Ser.* **260**, 83–96 (2003).

[36] FAO. *The State of World Fisheries and Aquaculture 2018 - Meeting the Sustainable Development Goals* (Rome, 2018).

[37] RAM Legacy Stock Assessment Database. Version 4.44-assessment-only. Released 2018-12-22 (2018).

[38] Schuhbauer, A. et al. *Global economic impact of scuba dive tourism* (2023); 10.21203/rs.3.rs-2609621/v1.

[39] DEMA. *2022 Diving Fast Facts: Fast Facts on Recreational Scuba Diving and Snorkeling*. 8 (2022).

[40] Kieran, D. The Size of The Scuba Diving Industry. *Medium* (2019). https://medium.com/scubanomics/the-size-of-the-scuba-diving-industry-573b8ac44c7c.

[41] Garrod, B. & Gössling, S. *New Frontiers in Marine Tourism: Diving Experiences, Management and Sustainability* (Elsevier, 2008).

[42] Cerrano, C., Milanese, M. & Ponti, M. Diving for science - science for diving: Volunteer scuba divers support science and conservation in the Mediterranean Sea. *Aquat. Conserv. Mar. Freshw. Ecosyst.* **27**, 303–323 (2017).

[43] SFIA. *2021 Fitness, and Leisure Activities Topline Participation Report* (2021).

[44] Cabral, R. B., Millage, K. D. & Mayorga, J. emlab-ucsb/ps-tourism. 10.5281/zenodo.8034977. (2023).

[45] South, A. rnaturalearth: World map data from natural earth. The R Foundation (2017); https://cran.r-project.org/web/packages/rnaturalearthdata/index.html.

