# Supplementary material for: Marine protected areas for dive tourism
Source: Sci Rep. 2025 Feb 11;15:1923. doi: 10.1038/s41598-024-83664-1 (PMC11814124; doi:10.1038/s41598-024-83664-1)
Supplement: Supplementary file 1 — Supplementary Material 1 [file 41598_2024_83664_MOESM1_ESM.pdf]

Supplementary Information

Marine protected areas for dive tourism

Reniel B. Cabral, Katherine D. Millage, Juan Mayorga, Tia Kordell, Mukta Kelkar, Alicia M. Caughman, Fabio Favoretto, Anna Schuhbauer, Octavio Aburto-Oropeza, Enric Sala, Darcy Bradley

1. Theoretical framework

We aim to quantify the ecological and economic benefits of upgrading the protection status of all unprotected recreational dive sites to highly or fully protected marine protected areas (MPAs). MPAs improve the marine ecosystem within its borders, increasing the demand for dive tourism.

Less than 10% of the global ocean is in MPAs, with only 2.4% highly or fully protected[1]. Upgrading the protection status of MPAs will increase fish biomass and species diversity inside the MPAs, which attract tourists that value higher biomass and diversity of aquatic life in the ocean (Supplementary Fig. 1). The methods for evaluating the changes in biomass and diversity due to MPA upgrades are presented in Sections 1.5 and 1.6. Additionally, we account for the MPA "name effect" – the commonly observed phenomenon that even with no biological changes in the abundance and diversity of marine life in the ocean, the mere fact that a site is designated as an MPA attracts tourists – reported in the literature[2,3].

We partition the global ocean into pixels 50 km x 50 km in size. We use the commercially harvested species reported in Sala *et al.*[4] as model species for quantifying changes in fish biomass resulting from the implementation of MPAs and adapt the biodiversity model reported in Sala *et al.*[4] (see Sections 1.5 and 1.6 for methods).

Supplementary Fig. 2A illustrates how we model the effect of implementing an MPA on diving demand in each ocean pixel with existing scuba dive tourism. Consider an unprotected dive pixel *i* (i.e., not an MPA; hereafter denoted by the subscript "0"). The number of dives demanded in pixel *i* (Q_{d,i_0}) as a function of the price per dive (P_i) can be written as:

[1] $$Q_{d,i_0} = a - bP_i$$

where parameters *a* and *b* are constants. The parameter *a* is the number of dives that would be demanded when the price of diving is zero (i.e., $P_i = 0$). The parameter *b* is the slope of the demand curve.

The number of dives supplied by the industry in pixel *i* is given by:

[2] $Q_{s,i} = c + eP_i$

where parameters *c* and *e* are constants. The parameter *c* is the quantity of dives that would be supplied when the price of diving is zero ($P_i = 0$). The parameter *e* is the slope of the supply curve.

1.1. Supply curve for scuba diving

Our empirical analysis indicates that the price per dive is nearly constant across countries and regions (see Section 2.4.1). This implies that the supply curve is horizontal (Supplementary Fig. 2B). A horizontal supply curve implies that the dive industry operates in a globally competitive market, and therefore, the price per dive remains constant regardless of the amount of diving being supplied[5].

1.2. Demand curve for scuba diving with no dive fees and no MPAs

On the demand side, we need to estimate *a* and *b*. We collect spatially explicit diving data globally and estimate the current number of dives demanded per non-MPA pixel ($Q^*_{i_0}$) and the price per dive ($P^*_{i_0}$). Assuming the scuba diving market is currently in equilibrium, we can substitute current dive numbers and price information in Eq. (1) for each pixel *i* :

[3] $Q^*_{i_0} = a - bP^*_{i_0}$

We collected price information from thousands of dive sites globally (see Section 2.4.1). Although the price per dive is horizontally flat on average, variations around the mean dive price exist. We assume that the upper 99th percentile of the empirically derived dive price data constitutes our choke price C_{i_0} or the price at which no tourist will pay to dive in pixel *i* if it is unprotected. Using dive shop level price data for one-tank and two-tank diving, we found the upper 99th percentile of the price per dive to be US$220.32. At the choke price ($P_i = C_{i_0}$), the number of dives demanded is zero (i.e., $Q_{d,i_0} = 0$). Substituting into Eq. (1):

[4] $0 = a - bC_{i_0}$

and by solving Eq. (4) for *a*:

[5] $a = bC_{i_0}$

Substituting Eq. (5) into Eq. (3) and solving for *b*:

[6] $$b = \frac{Q^*_{i0}}{C_{i0} - P^*_{i0}}$$

Substituting Eq. (6) into Eq. (5) allows us to solve for *a*:

[7] $$a = \frac{C_{i0} Q^*_{i0}}{C_{i0} - P^*_{i0}}$$

1.3. Demand curve for scuba diving with dive fees and/or MPAs

Tourism user fees can be used by managers and private sectors to monetize MPA benefits[6]. Such fees are commonly implemented to generate revenues for both terrestrial and marine settings and serve various purposes: limiting visitor numbers at popular sites or fragile sites, offsetting the costs of visitation, or providing funding for monitoring or maintenance[7–9]. Diving fees are one such type of tourism user fees that have been implemented to help finance MPAs by tapping into the recreational (or economic) value that tourists (or operators) derive from MPA use[10]. In places where such systems have been implemented, studies have shown that fees have largely been set too low to cover the actual costs associated with tourists' use of the MPA[11]. Nonetheless, the potential of diving fees to help finance MPAs is well recognized, and many studies suggest that divers would be willing to pay more for access to MPAs[12,13].

Both the MPA-induced changes in the biological conditions of the dive site and dive fees affect the demand for diving. Improvements in the biological conditions of dive sites will attract more divers (and/or encourage divers to dive more) and increase their willingness to pay to dive. Imposing dive fees will offset increased willingness to pay, resulting in reduced demand for diving if fees are set too high (as such, a dive fee will always result in reduced demand for diving).

With a dive fee and/or MPA, the demand for diving can be expressed as:

[8] $$Q_{d,i_{mpa/fee}} = a - bP_i + \Delta Q_{i_{mpa/fee}}$$

where $\Delta Q_{i_{mpa/fee}}$ represents the horizontal shift in the demand curve due to the MPA and dive fee.

Since we assume that the current number of dives at a site is in equilibrium when deriving the demand curve per dive pixel, the demand curve we have generated already accounts for the effect of any diver congestion (or decongestion). The demand curves describe the expected changes in the number of dives as dive prices change. The application of dive fee and/or MPA vertically or horizontally shifts the demand curve[3,14].

1.4. Quantifying changes to dive tourism benefits

There are three relevant benefits metrics that can be generated when mapping the supply and demand curves: dive revenue, producer surplus, and consumer surplus. Since we assume the supply curve for dive tourism is horizontal, the producer surplus is zero (0).

The application of a dive fee and/or an MPA affect(s) the demand for diving. Changes in the number of dives at a pixel and the cost of diving have a direct effect on the economic benefits derived from dive tourism.

The improvements in the biological condition in dive pixel *i* due to the MPA, together with the MPA name effect, will increase the willingness to pay of divers to dive at pixel *i*, resulting in an upward shift in the demand curve. As a consequence, there will be more dive demanded at pixel *i*. On the other hand, charging dive fees for diving at pixel *i* reduces the willingness to pay of divers to dive in pixel *i* (because of the increased total cost of diving). Consequently, there will be less dive demanded at pixel *i* compared to the case where no dive fee is collected (see Supplementary Fig. 3).

We denote the dive fee charged to divers for diving at pixel *i* as F_i. This dive fee can be imposed regardless of whether pixel *i* is an MPA. Also, we denote the change in the willingness to pay of divers to dive in pixel *i* caused by the MPA as ΣWTP_{mpa}. The summation term is added to indicate that various components cause a change in the diver's willingness to pay, particularly increases in fish biomass and biodiversity and the MPA name effect. Since both the implementation of the MPA and the dive fee shift the demand curve, we can use geometry to calculate the expected change in the number of dives. Using ratio and proportion, we have (Supplementary Fig. 4):

[9] $$\frac{C_{i_0} - P^*_{i_0}}{Q^*_{i_0}} = \frac{\Sigma WTP_{mpa} - F_i}{\Delta Q_{i_{mpa/fee}}}.$$

Solving Eq. (9) for the change in the number of dives or $\Delta Q_{i_{mpa/fee}}$ gives:

[10] $$\Delta Q_{i_{mpa/fee}} = \frac{Q^*_{i_0}(\Sigma WTP_{mpa} - F_i)}{C_{i_0} - P^*_{i_0}}.$$

The change in the revenue of the dive industry from pixel *i* due to the implementation of a diving fee and/or MPA (Supplementary Fig. 4) is:

[11] $$\Delta DiveRevenue_i = P^*_{i_0} \Delta Q_{i_{mpa/fee}}.$$

The dive fee revenue generated from implementing a dive fee in pixel *i* is:

[12] $$FeeRevenue_i = F_i(Q^*_{i0} + \Delta Q_{i_{mpa/fee}}).$$

The change in consumer surplus at site *i* equals the consumer surplus after the implementation of the dive fee and/or MPA minus the consumer surplus without the dive fee and/or MPA. The latter is given by the formula $0.5Q^*_{i0}(C_{i0} - P^*_{i0})$. The former can be derived using the formula $0.5P'(Q^*_{i0} + \Delta Q_{i_{mpa/fee}})$ where P' is shown in Supplementary Fig. 4. We can exploit the geometry in Supplementary Fig. 4 to derive P'. Using ratio and proportion, we have:

[13] $$\frac{C_{i0} - P^*_{i0}}{Q^*_{i0}} = \frac{P'}{Q^*_{i0} + \Delta Q_{i_{mpa/fee}}}$$

which can be rewritten as:

[14] $$P' = \frac{(Q^*_{i0} + \Delta Q_{i_{mpa/fee}})(C_{i0} - P^*_{i0})}{Q^*_{i0}}.$$

We then calculate the change in consumer surplus as:

[15] $$\Delta CSurplus_i = 0.5\frac{(Q^*_{i0} + \Delta Q_{i_{mpa/fee}})^2(C_{i0} - P^*_{i0})}{Q^*_{i0}} - 0.5Q^*_{i0}(C_{i0} - P^*_{i0}).$$

1.5. Modeling biomass accrual and spillover in response to protection upgrades

We assume that any dive pixel whose protection status was upgraded has the potential to experience an increase in fish biomass within its borders as a result of eliminating fishing. The amount by which biomass will increase depends on the degree of biomass depletion at the site and the biology of the target species (e.g., species mobility and growth rate). Sala *et al.*[4] utilized fisheries data for 1,150 commercially relevant marine stocks (representing 811 species) to parameterize their model estimating food provisioning benefits from fisheries. We performed an extensive literature review and built machine learning models to estimate the pelagic larval duration and home range parameters information for as many of the 811 species reported in Sala *et al.*[4] as possible (see Sections 2.1 and 2.2). Considering only those stocks whose geographic range intersects with any of the dive sites and with complete biological parameters, our final database includes 813 commercially relevant fish stocks (representing 599 species), comprising 74% of the total carrying capacity from Sala *et al.*[4].

Using data from Sala *et al.*[4], we first calculate the equilibrium biomass per stock for each pixel, assuming a business-as-usual scenario where there is no change in the protection level of dive sites. We then calculate the equilibrium biomass for the scenario where the protection status of all unprotected dive sites is upgraded into highly/fully protected MPAs. When the protection status of pixel *i* is upgraded, we assume that the biomass density in the surrounding fishing areas will be unchanged. This assumption implies that fishers will capture all adult biomass spillover and larval subsidy generated by the MPA.

While tourism benefits from MPAs are evaluated at steady-state biological conditions, our modeling framework requires us to model population dynamics as a difference equation that necessitates running our model forward until steady-state is reached. We assume that fish population growth and adult movement operate on a yearly basis. Population growth is driven by larval reproduction. Some of the larvae produced in each pixel settle within their natal site (self-seeded), and others are transported to and settle in adjacent pixels. The extent of larval transport is determined by how long the larvae stay suspended in the water column (i.e., the pelagic larval duration or PLD).

The biomass of stock *x* in pixel *i at* time *t+1* is given by:

[16] $$B_{x,i,t+1} = movement(B_{x,i,t}) + growth_{x,i,t} - harvest_{x,i,t}.$$

Adult population redistribution and larval production depend on adult biomass, and therefore, the sequence by which these processes operate needs to be specified. Initially, the adult biomass of stock *x* in pixel *i* reproduces and releases its larvae. These larvae are dispersed and will contribute to population growth at different sites after the adult population is redistributed. Whether the harvesting outside the MPA is performed before or after population growth is irrelevant, given that we assume the biomass density in fished areas remains constant.

The biomass of stock *x* at site *i* right after performing the movement operation is given by:

[17] $$movement(B_{x,i,t}) = s_{x,i\to i}B_{x,i,t} + \sum_{j\neq i} s_{x,j\to i}B_{x,j,t}$$

where $s_{x,i\to i}B_{x,i,t}$ *is* the portion of adult biomass of stock *x* from pixel *i* that stays in pixel *i*. The remaining biomass (i.e., $(1 - s_{x,i\to i})B_{x,i,t}$) moves to other pixels. The term $\sum_{j\neq i} s_{x,j\to i}B_{x,j,t}$ is the sum of all biomass of stock *x* from other pixels that move to pixel *i*.

For the purposes of this analysis, we are mainly interested in quantifying the build-up of biomass inside the MPA, as this is one factor influencing the demand for dive tourism. Inside the MPA, we assume harvest to be zero (0), so we can remove the harvest term from Eq. (16). In fished areas, fish biomass will not change because of our constant biomass density assumption

in the fishing area.

The maximum potential contribution of stock *x* biomass at time *t* in pixel *i* to the growth of stock *x* is $r_x B_{x,i,t}$, where r_x is the population growth rate. We assume that settling larvae will experience density-dependent growth (i.e., each pixel has a carrying capacity for stock *x*, $K_{x,i}$, such that it can only accommodate a certain population size). Since larvae will also be dispersed to other pixels–the extent of which depends on the PLD of the stock–the growth contribution of larvae produced in pixel *i* to the biomass of stock *x* in pixel *i* is given by $\rho_{x,i \to i} r_x B_{x,i,t}(1-(B_{x,i,t}/K_{x,i}))$ where $\rho_{x,i \to i}$ is the proportion of viable larvae of stock *x* produced in pixel *i* that settles in pixel *i*.

Biomass from other sites also contributes larvae to pixel *i*. The larval contribution of biomass from other pixels *j* to pixel *i* is:

[18] $$\sum_{j \neq i} \rho_{x,j \to i} r_x B_{x,i,t}(1-\frac{B_{x,i,t}}{K_{x,i}}).$$

Therefore, the population growth of stock *x* in pixel *i* gained from larval contributions from all sites (i.e., including pixel *i*) is:

[19] $$growth_{x,i,t} = \rho_{x,i \to i} r_x B_{x,i,t}(1-\frac{B_{x,i,t}}{K_{x,i}}) + \sum_{j \neq i} \rho_{x,j \to i} r_x B_{x,i,t}(1-\frac{B_{x,i,t}}{K_{x,i}}).$$

Altogether, the biomass of stock *x* in MPA pixel *i* (i.e., *i*=MPA) at time *t+1* following dive site protection upgrades is:

[20]
$$B_{x,i_{mpa},t+1} = s_{x,i \to i} B_{x,i,t} + \sum_{j \neq i} s_{x,j \to i} B_{x,j,t} + \rho_{x,i \to i} r_x B_{x,i,t}(1-\frac{B_{x,i,t}}{K_{x,i}}) + \sum_{j \neq i} \rho_{x,j \to i} r_x B_{x,i,t}(1-\frac{B_{x,i,t}}{K_{x,i}}).$$

We generate the equilibrium biomass for each MPA pixel by simultaneously running the biomass equation for all MPA pixels worldwide for 100 time-step iterations.

We derive the stock geographic ranges from Aquamaps predicted species distribution maps. Aquamaps produced a species-specific probability of occurrence maps with values ranging from 0 to 1, indicating the likelihood of the species being found in a specific area of the ocean. We apply a threshold of 0.5 to the Aquamaps predicted species distribution maps to generate the geographic range of stocks (i.e., the probability of occurrence must be 0.5 or above in pixel *i* for that pixel to be considered part of the stock range). The growth rate (r_x) and carrying capacity (

K_x) for each stock are derived from Sala *et al.*[4]. We assume the carrying capacity for each stock is homogeneously distributed across the entire geographic range of the stock (i.e., $K_{x,i} = K_{x,j}$).

1.6. Modeling biodiversity effects in response to protection upgrades

We used a similar approach to model the effects of MPAs on biodiversity as done by Sala *et al.*[4]. Biodiversity benefits are defined as the weighted sum of the marginal gain in the persistence of marine species resulting from the removal of abatable impacts relative to business as usual. We consider the native ranges of 4,242 marine species[15] that are directly or indirectly affected by fishing as reported by the International Union for Conservation of Nature (IUCN) or reported in global catch databases[16,17]. We use the approach of Sala *et al.*[4] to estimate the benefits that could be gained for each species from additional protection conferred by MPAs. These benefit functions resemble species-area relationships, meaning that the marginal benefits from each additional unit of protection are diminishing. We use the approach of Sala *et al.*[4] to define the biodiversity benefit (*S*) of protecting a set of pixels (*s*) as:

[21] $$S_s = \sum_x \gamma_x (X_{x_s})^{w_x}$$

where γ_x represents the weight given to stock *x* (see Section 2.3) and w_x dictates the curvature of the power function for stock *x* (diminishing marginal benefits as in a species-area curve). As in Sala *et al.*[4], we set w_x equal to 0.25 for all species based on a typical species-area relationship exponent value (i.e., z value) between 0.2 and 0.3[4,18]. X_{x_s} is the fraction of the stock *x's* total habitat that remains suitable given the set of protected pixels *s* and is defined as:

[22] $$X_{x_s} = \sum_{i \in s} v_{i,x}^{\text{in}} + \sum_{i \notin s} v_{i,x}^{\text{out}}$$

where $v_{i,x}^{\text{in}}$ and $v_{i,x}^{\text{out}}$ correspond to the fraction of the stock *x's* total habitat that remains suitable in pixel *i* if *i* is an MPA and if pixel *i* is left unprotected. This allows the model to account for both the value of protected and unprotected pixels toward the persistence of a species. $v_{i,x}^{\text{in}}$ and $v_{i,x}^{\text{out}}$ are defined as:

[23] $$v_{i,x}^{\text{in}} = v_{i,x_0}(1 - I_{u_i})$$ and

[24] $$v_{i,x}^{\text{out}} = v_{i,x_0}(1 - I_{u_i})(1 - I_{a_i})$$

where v_{i,x_0} is the fraction of total suitable habitat for stock *x* present in pixel *i*, I_{u_i} is the fraction of the habitat in pixel *i* that may be lost due to un-abatable impacts (sea surface temperature rise, light pollution, organics and nutrient pollution, ocean acidification, shipping, and sea-level rise) and I_{a_i} is the fraction of the habitat that may be lost as a result of abatable impacts (artisanal fishing, commercial fishing classified in pelagic high-by-catch, pelagic low-by-catch, demersal destructive, demersal non-destructive high by-catch, and demersal non-destructive low by-catch). We use the estimates of these parameters from Sala *et al.*[4], which were derived using data from 2009 - 2013 on human impacts[19].

2. Model Parameterization

2.1. Adult mobility

Adult mobility was modeled using species' home ranges (in km^2). Home range values for a total of 667 out of the 811 commercially relevant marine species (Supplementary Fig. 5) from Sala *et al.*[4] were predicted by a random forest regression model in *R* using the *tidymodels* package[20,21]. The model predicted home range values based on the intrinsic growth rate, carrying capacity, species length, species trophic level, movement keyword, and geographic range size. Empirical home range values for training the random forest were collected via literature review, which used a variety of field and analytic techniques for home range estimation. A total of 221 empirical home range values were collected, a number of which describe the same species. Life history information was obtained from Fishbase[22], and geographic ranges were obtained from Aquamaps[15]. Geometric means were calculated for species with home range values from multiple studies, resulting in 67 unique species with observed home ranges and all required model data (i.e., life history and geographic range data).

Observed home range data was split into a training (70% of data; 46 observations) and a testing (30% of data; 21 observations) dataset for model fitting and evaluation. This split was stratified by family in order to get a representative sample of fish families in the training set. All numeric predictors were normalized, and home range values were log-transformed due to the large range of values to prevent an over-influence of large values on the model predictions. The testing data set root mean square error or RMSE (3.35) and R^2 (0.55) values indicated good model fit. This model accurately predicted home range values within either the correct order of magnitude (10X) or one order off (80.95%). In general, the model underpredicted correct home range values leading to conservative estimates of fish movement.

In parameterizing the adult mobility component (s_x) from Eq. (21), we assume that the home range (in km^2) represents the area of a circle. So, the biomass at pixel *i* can only redistribute to other pixels within the radius $\zeta = \sqrt{homerange/\pi}$. We give the empirically derived home range data priority when available and then use the predicted home range data for species with no empirical information.

2.2. Larval dispersal

The distribution of larvae produced at a specific site to other sites can be represented by a Gaussian dispersal kernel. The spread of the kernel is driven by the amount of time spent by larvae in the pelagic (i.e., stock-specific pelagic larval duration, PLD_x) and the ocean currents.

We assume that the larvae spread symmetrically from the source (i.e., we are neglecting the drift component of the larval dispersal). The proportion of larvae of stock *x* from pixel *j* that settles in pixel *i* is given by:

[25] $$\rho_{x,j\to i} = \frac{1}{2\pi\sigma^2_{larvae,x}} e^{-\frac{d^2_{j\to i}}{2\sigma^2_{larvae,x}}}$$

where $d_{j\to i}$ is the distance from pixel *j* to pixel *i* and $\sigma_{larvae,x}$ is the spread of the larval dispersal kernel of stock *x*. The relationship between $\sigma_{larvae,x}$ and the pelagic larval dispersal of stock *x* (PLD_x) has been empirically derived by Siegel *et al.*[23] and is given by:

[26] $$\sigma_{larvae,x} = 1.33\sqrt{\frac{\pi}{2}} PLD_x^{1.3}$$

We computed the distance between every pair of pixels in our global ocean grid using the *gridDistance* function of the *raster* R package[24]. This function estimates the distance from a cell to all other cells, assuming the path must cross the center of neighboring cells. Each cell has eight (8) neighboring cells in this implementation, and the path cannot cross non-ocean cells. Total distance is then the sum of local distances between neighboring cells.

We calculate $\sigma_{larvae,x}$ for each stock. We constrained the distance matrices to $3\sigma_{larvae,x}$ (i.e., larvae will not propagate beyond this distance from the source). Ninety-nine and seven-tenths percent (99.7%) of the area of the dispersal kernel is already captured within a distance of $3\sigma_{larvae,x}$.

Our goal was to generate PLD values from which we could calculate $\sigma_{larvae,x}$ for as many of our 811 commercially relevant marine stocks as possible. Marshall *et al.*[25] reported life history data for 806 invertebrate species, 254 of which have "planktonic time" data. We were able to use data from Marshall *et al.*[25] for two invertebrate species in our list of commercially exploited stocks. Ramesh *et al.*[26] provide 1798 entries pertaining to life history data, but the actual number of species represented by this data set is less because there are duplicates. 361 entries in this data set contain PLD data. We were able to match data from Ramesh *et al.*[26] to 65 commercially exploited stocks on our list. Fontoura *et al.*[27] reported life history data for 528 unique species with PLD data. We were able to match data from Fontoura *et al.*[27] to 44 of our commercially exploited stocks.

We used a similar random forest regression approach (as that used to estimate home range) to estimate PLD in days using empirical data for 91 species. PLD values were averaged for species with multiple empirical PLD values and values for Elasmobranchs were removed, as they do not have pelagic larval stages. PLD values in days for 610 out of 811 of the commercially relevant species from Sala *et al.*[4] (Supplementary Fig. 6) were then predicted using intrinsic growth rate, carrying capacity, species length, trophic level, and movement keywords.

All numeric predictor variables were normalized, then the model was trained using 71 observations (80% of the data) for species with known PLD, while 20 observations (20% of the data) were withheld for model testing. As with the home range, this training/testing split was stratified by family. The testing data RMSE for this model was 114.08 and the R^2 was 0.19, indicating only a weakly positive relationship between predicted and observed testing values. PLD values were then converted to months (i.e., PLD/30). The correct month value was predicted by this model in 30% of predictions and was off by only a single month in another 55% of predictions. As with home ranges, PLD values were generally underestimated, leading to a conservative estimate for unknown values.

2.3. Species weights

Sala *et al.*[4] weighted species as a function of their extinction risk (*EX*), functional distinctiveness (*FD*) and evolutionary distinctiveness (*ED*) such that not all species would contribute equally to the biodiversity benefit. These weights were calculated based on the following relationship:

[27] $$\gamma_x = EX_x(FD_x + ED_x); \forall x \in \text{species}$$

where γ_x is the weight for stock *x*. Sala *et al.*[4] calculated *EX* using the IUCN classifications of extinction risk. Stocks' *FD* were calculated based on the extent to which the traits of each

species were distinct relative to the traits of all other species from the same taxonomic class. Sala *et al.*[4] calculated the functional distinctiveness of stock *x* (FD_x) as:

[28] $$FD_x = \sum_{x=1; y\neq x} N d_{x,y} N - 1$$

where $d_{x,y}$ is the functional distance between species *x* and *y*, and *N* is the total number of species in the taxonomic class. When all species in a taxonomic class have the same values, *FD* is 0; when species *x* is maximally differentiated from all other species, FD_x is 1. Sala *et al.*[4] also estimated species' evolutionary distinctiveness using a similar approach: the evolutionary distinctiveness of species *x* (ED_x) is high when that species is isolated in its phylogenetic tree (i.e., that species has a long unshared branch length compared to all the other species).

This method resulted in species in the subclass Elasmobranchii (sharks, rays, skates, and sawfish; 34.0%) being given the greatest aggregate weight, followed by species in the class Actinopterygii (ray-finned fishes; 27.3%), species in the subphylum Anthozoa (sea anemones, stony corals, and soft corals; 16.5%), birds (5.2%), mammals (5.0%), species in the class Malacostraca (crabs, lobsters, crayfish, shrimp, krill, prawns, woodlice, amphipods, mantis shrimp; 3.8%), and then cephalopods (squid, octopus, cuttlefish, and nautilus; 2.1%). All remaining species accounted for the remaining 6.1% (Supplementary Table 2).

We believe the methodology of Sala *et al.*[4] weighting species' relative impacts on the total biodiversity score based on extinction risk, functional distinctiveness, and evolutionary distinctiveness is likely to encompass many of the attributes that make species attractive to divers. Nonetheless, it is possible that the types of species valued most by divers might be different from those weighted most highly based on this method. Some studies have attempted to quantify diver preferences and their willingness to pay to see certain types of species underwater (e.g., ref. [28]), but such preferences are likely to vary depending on diver demographics–particularly dive experience–and region. To the best of our knowledge, no study has tried to estimate divers' relative preferences for different types of species that might be observed while diving (i.e., studies have only tried to quantify the willingness to pay for greater abundance of certain species or overall species diversity, see Sections 2.7.2 and 2.7.3). Though we were unable to find literature specifically investigating divers' relative species preferences, we believe some of these preferences might be revealed in other ways and we briefly discuss these here and how they support or differ the species weights used in our model.

Consider the species (or general groups of species, e.g., sharks, sea turtles, reef fishes) that dive operators advertise. We believe these could be reflective of the types of species divers like to see while diving. As part of the general information collected for each of the dive operators represented in our global database of diving prices (see Section 2.4.1 for more on the curation of this database), we recorded mentions of specific species (or groups of species) advertised as those likely to be seen while diving with that operator. In total, we recorded 1,800 mentions of

different species or groups of species that we were able to match to a taxonomic class from the websites of 240 different dive operators. We then calculated the percentage of total mentions corresponding to different taxonomic groups (Supplementary Table 2). Though not a perfect analog, we can compare this breakdown to the aggregate species weights obtained based on extinction risk, functional distinctiveness, and evolutionary distinctiveness by taxonomic group.

The majority of mentions were of species (or groups of species) of the class Actinopterygii and subclass Elasmobranchii. Together these two taxonomic groups accounted for 67.6% of all mentions recorded from the dive operator websites (compared to 61.3% of the aggregate species weight). However, species of the class Actinopterygii were responsible for a much greater percentage of the total mentions (49.1%) than species of the subclass Elasmobranchii (18.5%). This could suggest that divers place greater importance on seeing ray-finned fishes than seeing sharks and rays, but we feel this is unlikely to be the case given the high value of sharks and rays found in studies on diver WTP. We believe this discrepancy was largely due to the resolution with which operators tended to advertise ray-finned fishes (to the individual species level) versus sharks and rays (e.g., “sharks”). This often resulted in the same operator mentioning a greater number of ray-finned fishes as compared to elasmobranchs. It is also important to note that many of the species of the class Actinopterygii advertised on operator websites are not included in our model as they are not commercially relevant. Together we believe these factors make the lower aggregate weight placed on ray-finned fishes in our model appropriate in the context of diver preferences.

Species in the subphylum Anthozoa (sea anemones, stony corals, and soft corals; 16.5%) make up a much greater aggregate weight (16.5%) as compared to dive operator mentions (1.1%). We believe this discrepancy was largely because these species were often implied by dive operators (e.g., “reef diving”) instead of being individually identified (e.g., “brain corals”). Our method of counting species mentions likely greatly under-recorded coral species. Additionally, the presence of many ray-finned fishes and other species advertised by dive operators is only possible because Anthozoans are also present in those areas, something that we do not take into account. For these reasons, we believe it is appropriate that Anthozoans receive a higher aggregate weight in our model.

Marine reptiles made up a much greater percentage of total diver operator mentions (5.9%) as compared to the aggregate weight of these species in our model (0.5%). This is probably the only taxonomic group for which we feel the method used to calculate species weights may actually underestimate the importance of the group to divers. Sea turtles are very popular with divers, as evidenced by the high value of these species found in studies on diver WTP[29]. Our model may therefore underestimate changes in dive demand resulting from increases in the abundance of sea turtles because of the low aggregate weight of marine reptiles.

2.4. Price per dive

2.4.1. Creation of a globally representative database of diving prices

To curate a geographically representative dataset of prices for scuba diving, we utilized a global Google Maps-derived database of dive operators (*N* = 11,132; Supplementary Fig. 7A) as a sampling frame from which to extract data directly from operators on their prices[30]. This database was generated using the Google Maps API to look for query parameters based on business categories registered in Google. Business categories are standardized in Google to enhance searches, so businesses registering within the platform are advised to specify their category correctly. Schuhbauer *et al.*[30] used the "Diving center" business category as a keyword. Searches were conducted in English first, but the primary language for each country (if different than English) was also used. Searches were conducted at the country level and were repeated until global coverage was reached. Each country's results were joined in a single dataset where they were inspected for incorrect coordinates and mismatched business categories. Duplicates with the same coordinates were eliminated. As a preliminary check of validity, Schuhbauer *et al.*[30] created a program to ping each website listed in the dataset to see if it was active.

To collect price data for this study, we filtered the global operator database created by Schuhbauer *et al.*[30] to only include operators for which an active website had been obtained (*N* = 9,909) and then employed a geographically stratified sampling method to partition the filtered database into five strata defined based on region. We aimed to collect price data from approximately 10% of all operators in the dataset during the course of multiple data collection phases.

In the first phase, we collected two random samples, each comprising 2.75% of the total number of operators in each stratum across the entire filtered dataset. The first two samples overlapped slightly, with a randomly selected 25 operators (0.25% of the filtered dataset) to allow for QA/QC between data collectors. The same process was repeated in the second data collection phase to generate two more random samples each comprising 2.75% of the total total number of operators in each strata from the filtered dataset. Operators sampled in the first phase were removed from the sampling frame so they could not be selected to be sampled twice. We again allowed for the two samples to share 25 randomly selected operators to allow for QA/QC between data collectors. Across both phases, 1,021 distinct operators were sampled. The total breakdown of operators sampled by region is shown in Supplementary Fig. 7B-C.

For each operator sampled, general information was first collected about the website and business, and we predefined a number of cutoff points at which data collection would stop if certain conditions were not met. If the URL did not lead to a valid website, the first such cutoff point was reached, and data collection was stopped. Of the 1,021 operators sampled, 948 had a valid URL. If the URL was valid, the original language of the site was next recorded and if the site was not written in a language understandable by our data collectors (English or Spanish), Google Translate was applied to the site (and its use was recorded). At this point, data

collectors were asked to make a determination if they could understand enough of the information on the site to proceed. If not, the second cutoff point was reached and data collection stopped. Of the 948 operators with a valid URL, 902 were deemed to be comprehensible with or without the use of Google Translate. If understandable, the data collector was next asked to determine whether the website pertained to a business related to the diving industry in any capacity (e.g., recreational or technical scuba diving, snorkeling, freediving, commercial diving). If not, the third cutoff point was reached and data collection stopped. Of the 902 comprehensible websites, 762 were found to pertain to dive-related businesses. The data collectors then determined whether the business offered marine diving services. The business name (if different from that listed in the original operator database), location, and any affiliated certification agencies (PADI, NAUI, SSI or TDI, or Other) were also recorded. If the website contained mentions of any specific species or attractions (e.g., wreck diving, kelp diving, cavern diving) or contained photos of such species or attractions these were also noted. In total, 534 businesses (52.3% of all operators sampled) were found to offer marine diving classes or services (Supplementary Fig. 8).

Once the general information had been collected for each operator, the data collectors then collected any available price information for the following activities: 1-tank dives, 2-tank dives, multi-tank and/or multi-day dive packages, snorkeling trips, Discover Scuba packages (or equivalent introductory dives for non-certified divers), Open Water certification courses (or equivalent level 1 instructional courses), Advanced Open Water certification courses (or equivalent level 2 instructional courses), Rescue certification courses (or equivalent level 3 instructional courses), and specialty dives (e.g., night dives, diving with sharks or whales). Price data was only collected for operators offering marine diving services and for activities advertised to take place (at least partially, in the case of instructional courses) in a marine setting. Data on each available price was collected in the local currency available on the website, and any available information was noted about the location of the dive, whether the dive would be executed from shore or from a boat (for the open water dives, in the case of certification courses), whether the dives would be conducted from a multi-day liveaboard vessel, whether the price included full equipment rental (defined as including anything more than tanks and weights), and whether the price included food and drink.

Once the sampling phase was complete, all four samples were collated and cleaned. Duplicates included for QA/QC were randomly selected for removal, and currency codes were standardized. Prices were then converted into USD using exchange rates from June 22, 2022 from the *quantmod* package in R.

The median prices for day trip diving activities (1-tank dives, 2-tank dives, and snorkeling trips) were US$59, US$110, and US$47, respectively (Supplementary Table 3). There were no consistent differences in price by continent, though many of the higher price outliers were associated with shops located in the Americas (Supplementary Fig. 9). Prices per dive calculated from 1-tank dives, 2-tank dives, and n-tank dive packages were used to create a single database of price per dive. In cases where the same operator reported prices for more

than one of those activities, the 1-tank dive price was given first priority, followed by the 2-tank dive price–the other prices were then removed so that there was only one per-dive price for each operator. Per-dive prices calculated from these different activities were combined in a single database in order to encompass a greater geographic area (i.e., 1-tank dives are more common in certain areas and vice versa for 2-tank dives). The calculated per-dive prices were then spatially allocated based on the geographic coordinates of the operators and binned into 50 km x 50 km pixels. The median price per dive was calculated for each pixel, for each country, for each region, and globally.

Some of the pixels in which dive operators are located correspond to valid ocean pixels used in our model (i.e., the operator is situated directly adjacent to the ocean), but many are located inland. We therefore consider three scenarios for how to spatially apply our data on dive prices to different areas of the ocean (Supplementary Figs. 10, 21):

1. The global median dive price is uniformly applied to all ocean pixels (reference case scenario).
2. The median dive price per country is uniformly applied to all ocean pixels falling under that country's jurisdiction. Ocean pixels corresponding to countries without data on dive prices receive the median price for the region (sensitivity analysis scenario - see Section 3.2).
3. Dive prices are interpolated for all pixels based on the median price per dive per pixel (sensitivity analysis scenario - see Section 3.2).

2.4.2. Regional and national statistics on the prices of scuba diving

Some previous studies have quantified or made reference to the price of scuba diving in specific locations. We gathered data on dive prices from published and gray literature using Web of Science and Google Scholar for comparison with the database on global diving prices we curated. Prices of scuba diving activities reported in the literature are summarized in Supplementary Table 4.

In the East Asia and Pacific region, Clua *et al.*[31] reported a price per dive of US$62.50 - $75 for diving in Moorea, French Polynesia. It was noted that this range encompassed prices for both domestic and international divers. Pascoe *et al.*[32] conducted a survey of 578 divers at popular dive sites in Indonesia, Malaysia, and Thailand to estimate the consumer surplus associated with coral reef-based dive tourism in the region. They sampled both international and local divers and asked them about their total travel costs associated with the entire dive trip, which they normalized based on the total number of dives in the trip. The average cost per dive reported for international visitors was US$505.40 across all three countries. This cost is quite high because it includes the cost of the dive package, transport costs to the dive site from the diver's home (including international travel costs), accommodation, and other related expenditures. Given that our dataset of dive prices does not account for these types of costs, this value is not directly comparable to our data. Nonetheless, the average cost per dive reported for domestic visitors by Pascoe *et al.*[32] was US$60.75 across all three countries

surveyed. This cost also includes transport, accommodation, and other costs, but such costs were noted to be much lower (or zero) for this group. We therefore believe it is reasonable to discern from this study that the actual price per dive in this area was likely lower than US$60.75. For comparison, the median price per dive from all operators in the East Asia and Pacific region calculated from our database is US$62.43.

Studies reporting dive prices for Europe and Central Asia are more limited, but Rodrigues *et al.*[33] performed a choice experiment to elicit preferences of scuba divers in the Medes Islands MPA in the Spanish Mediterranean. They report that the price for a single 50-minute dive in the MPA was approximately €50 (US$66.42) in 2013. This is higher than the median price per dive calculated for all operators in the Europe and Central Asia region from our database of US$46.84.

Multiple studies have reported dive prices for locations in Latin America and the Caribbean. In their assessment of the dive tourism industry in Mexico, Arcos-Aguilar *et al*.[34] surveyed 106 of the 264 active dive operators in the country in 2019. The main goal of this study was to estimate revenues associated with the diving industry. They asked operators to report the prices of the scuba and snorkeling trips and courses they offer. The survey only encompassed single-day trips, which commonly include two dives in the case of scuba trips. The average cost per scuba diving trip ranged from US$95.13 - $131.50 across the four regions surveyed. This would correspond to a price of US$47.57 - $65.75 per dive. In a different study, Schuhmann *et al.*[35] conducted a survey of divers in Barbados to assess their willingness to pay for biodiversity. They asked 165 recreational divers about the price of their most recent dive in the country. Respondents reported an average price of US$105.58 (± US$38.40), and it was noted that this price pertained to dive trips that usually included two dives. This would correspond to a price of US$52.79 per dive, which falls within the range reported by Arcos-Aguilar *et al.*[34]. The median price per dive calculated from our database for operators in Latin America and the Caribbean is very similar to the high end of the range reported by Arcos-Aguilar *et al.*[34] at US$67.50.

2.5. Suitability for recreational dive tourism

The activity of scuba diving has many different facets, including recreational (or sport) diving, technical diving, and commercial diving. Recreational divers make up the majority of dive tourists by far, and thus, we have limited our parameterization of the global demand curve for dive tourism to recreational diving. Recreational diving is generally considered to include all activities involving scuba diving that occur no deeper than 130 ft (40 m) and do not require decompression stops upon ascent[36]. Conversely, technical diving is considered to be any activity occurring beyond this depth, in overhead environments where the surface is not readily available and more specialized equipment is necessary (e.g., inside caves, shipwrecks, under ice), or requiring planned decompression stops upon ascent. This distinction isn't always clear cut, and some types of recreational dives may require advanced training or specialized equipment regardless of depth (e.g., diving on shipwrecks, inside caverns, diving in heavy currents, or poor visibility). Certain niche areas where "blue water" or "black water" diving is

undertaken in pelagic environments (e.g., shark diving in the Azores[37]) may also be difficult to classify based on the aforementioned definition of recreational diving, but these dive experiences make up a very small subset of all dive tourism.

In reality, depth is not the only factor determining whether or not an area is suitable for diving. Oceanographic conditions (e.g., currents, visibility, water quality, temperature) play a role, as does the type of ecosystem (or benthic structure) present in that location (e.g., coral reefs, kelp forests, sand flats, mangroves, rocky reefs). The majority of scuba diving in marine environments occurs in tropical or subtropical coastal and nearshore areas where coral reef ecosystems dominate[38,39], though diving also happens in more temperate ecosystems such as kelp forests and rocky reefs[40]. There may also be other factors dictating the suitability of a site for diving that are more difficult to observe, such as the ease of accessibility (e.g., entry and exit conditions, distance from a port or shore, distance from a tank filling station), the presence of hazards (e.g., vessel traffic, geopolitics of the area), or the availability of hospitality infrastructure nearby (e.g., hotels, restaurants).

To identify the ocean pixels suitable for recreational dive tourism in our model, we used a crowdsourced database of logged dives provided by Diveboard to identify where recreational diving currently occurs. We used the following criteria to define a “real” recreational dive. We kept all dives with a valid date that occurred at or shallower than 140 ft (or 43 m) to provide a slight buffer for the “industry standard” 130 ft (40 m) limit defined for recreational diving, knowing that divers occasionally exceed this limit for short periods of time. We then identified valid dive sites from the Diveboard database by removing those where the user failed to specify a location (identified by a default latitude and longitude of 0°) or specified invalid coordinates. Finally, we matched the database of valid recreational dives back to their corresponding dive sites to determine whether or not each 50 km x 50 km ocean pixel is suitable for recreational diving. For our model, the presence of any dive site in a pixel with a logged recreational dive between 2010 and 2020 resulted in the pixel being deemed to be suitable for recreational diving in this analysis (Supplementary Fig. 11).

2.5.1. Dive site jurisdiction and protection status

Our cleaned Diveboard dataset contains 43,023 dive sites, with at least one logged recreational dive made at that location between 2010 and 2020. For summary purposes, we used two sources to determine the entity in whose jurisdiction each site is located. Since many dive sites in this dataset are not located in marine environments, we first overlaid the Natural Earth country boundaries (10m scale) accessed through the *rnaturalearth* package[41] to determine the administering country or territory and the sovereign state associated with each non-marine dive site. We then overlaid EEZ boundaries (v11) from Marine Regions[42] to determine the administering country or territory and the sovereign state associated with each marine dive site. Supplementary Fig. 12 shows the number of recreational dive sites by land area and EEZ in our final dataset.

All dives made at dive sites located within the boundaries of an EEZ area as determined from the Marine Regions EEZ boundaries data were considered to be marine for the purposes of this analysis. We next determined the current protection status for the marine dive sites in our cleaned Diveboard database using the boundaries of global MPAs and other effective area-based conservation measures (OECMs) from the Marine Protection Atlas database (MPAtlas, 12/23/2021 version)[43]. Since the most recent data from MPAtlas no longer classifies protected areas as "Fully/Highly Protected" or "Less Protected/Unknown"—used by Sala *et al.*[4]—we recreated these classifications for the December 2021 version of the MPAtlas utilizing their original methodology.

In cases where a dive site is covered by multiple protected area designations, we retain the protected area designation conferring the highest protection status ("Fully/Highly Protected," "Less Protected/Unknown," "Designated & Unimplemented," "Proposed/Committed") or the oldest designation (if all designations have confer equal protection).

Of the 43,023 dive sites in our cleaned database, 64.17% are marine (or 27,609 established marine dive sites worldwide). Of the marine dive sites, only 15.48% are located in fully or highly marine protected areas (that is, no-take MPAs or protected areas in which only minimal subsistence or recreational fisheries are allowed) (Supplementary Fig. 13). Supplementary Fig. 14 shows the breakdown of marine dive sites by protection status for the 30 EEZ areas with the greatest number of dive sites.

2.6. Quantity of dives

2.6.1. Estimation of the total number of recreational scuba dives made annually

The number of recreational scuba dives made worldwide each year is unknown, but estimates of its likely order of magnitude exist. Here, we provide a brief overview of global estimates pertaining to the global number of scuba dives made each year and detail the logic behind the assumptions used to parameterize our model.

The Dive Equipment and Marketing Association (DEMA) is a non-profit trade association that collects and disseminates statistics related to the recreational diving and snorkeling industries[44]. DEMA regularly reports the results of extensive annual studies done by the Sports and Fitness Industry Association (SFIA) and the Outdoor Industry Association. Drawing on these studies, DEMA reports in the "2022 Diving Fast Facts" that there are as many as 6 million active scuba divers worldwide and there were 2.59 million active divers in the United States in 2020 (estimates for the number of active divers in the United States in previous years have ranged between 2.7 million and 3.5 million; ref. [44]). The definition of an "active" scuba diver is simply anyone who has participated in scuba diving but the SFIA 2021 Topline Participation Report[45]—from which DEMA sourced the statistic on the number of active American divers—breaks down the information further by defining two categories of participants: those who participate in scuba diving one to seven times per year ("casual" divers) and those who

participate eight or more times per year (“core” divers). We use these categories to extrapolate the number of dives made each year.

Of the 2.59 million active divers in the United States in 2020, 1.88 million identified as casual divers and 708,000 identified as core divers (Supplementary Table 5; ref. [45]). If we assume that the casual diver only makes a single dive per year and the core diver makes eight dives per year, this would result in a lower estimate of 7.54 million dives made by American divers each year. If we instead assume that the casual diver makes seven dives per year and the core diver makes 15 dives per year, this would result in a higher estimate of 23.8 million dives made by American divers each year. As a reasonable midpoint, let’s assume the casual diver makes on average four dives per year and the core diver makes on average 10 dives per year. This results in an estimated 14.6 million dives being made by American divers each year. We can then extrapolate these values globally, assuming that active divers outside of the United States have the same participation rates. Assuming there are 6 million active divers worldwide (as reported by DEMA[44]), the total number of dives made globally in a year would be between 17.5 and 55.1 million, with 33.8 million as a realistic central value (assuming a casual diver makes four dives per year and a core diver makes 10 dives per year).

Given the lack of reliable global statistics about the dive industry, a number of organizations and individuals interested in market data and research have generated their own estimates of the total number of scuba divers worldwide. We use these to help refine our estimate. In an article posted on *Medium*, Kieran[46] estimates the global number of divers. In their extrapolation, they make reference to the estimate of 2.6 million active divers in the United States from the 2021 SFIA report, but also reference a World Recreational Scuba Training Council (RSTC) Europe report, which claims that the number of active divers in Europe is between 3 and 4 million. Kieran uses this figure to argue that the global estimate of 6 million active divers stated by DEMA is likely too low. Citing other information suggesting that the sizes of the American and European markets are likely roughly equivalent, they go on to argue that it would be reasonable to assume that the rest of the world comprises an approximately equal market share, putting the total number of active divers worldwide in the ballpark of 9 million. We believe this to be a realistic assumption, as other sources suggest the total number of active divers worldwide to be higher than 6 million. For example, Garrod and Gössling[47] report that there could be up to 7 million active certified divers in the world, and Cerrano *et al.*[48] claim that recreational diving engaged around 20 million people worldwide (not all of whom remained active) between the mid-1980s and 2016. Assuming the same participation rates, if we instead assume there are 9 million active divers worldwide, our extrapolated estimate of the total number of dives made per year would increase to be between 26.2 and 82.7 million, with 50.7 million as a realistic central value. We use this higher estimate of 50.7 million total dives (in marine and freshwater environments), consider only the dives made in the marine environment in our model as the base case scenario, and perform model sensitivity with the lower and upper bounds of the dive estimates in the marine environment (see Section 3.1).

2.6.2. Spatial allocation of dives

We used the crowdsourced database of dives provided to us by Diveboard to spatially disaggregate our global estimate of 50.7 million recreational dives per year made in both marine and freshwater environments. No previous analyses have been done to assess how representative the Diveboard data are of all diving worldwide. Though the number of logged dives represented in this database is clearly not representative of the true magnitude of diving globally, we believe it contains the locations at which most recreational dives are made across the world, especially those made by dive tourists. Because this crowdsourced dataset was primarily generated by divers from the United States and Europe, we recognize that dive sites that are visited only by local divers outside of these regions may be underrepresented. Furthermore, given that freshwater diving in North America is quite prevalent (e.g., Florida caves, Mexican cenotes, the Great Lakes, Lake Tahoe, quarries throughout the US and Canada, Bonne Terre Mine in Missouri), and accounts for a significant number of dives recorded in Diveboard database, we may have underrepresented the proportion of marine diving in other locations outside North America. Nonetheless, American and European divers make up a significant portion of all dive tourists worldwide. Here we detail the process by which we used the Diveboard database to spatially allocate our global estimate of the total number of dives made annually. We summarize our review of reference points from the literature to assess whether this method may have over- or under-estimated the total number of dives being made in different areas in the next section.

To estimate the proportion of total diving occurring within each ocean pixel, we first matched all dives logged between 2010 and 2020 in the Diveboard database to the sites at which they occurred. We then used the proportion of the total number of dives that were made at each site (both marine and freshwater) to allocate the estimated 50.7 million dives that are made each year. Accounting for rounding (so that no site could have partial dives), we spatially allocate 50,684,776 dives. Only the dives made at sites considered to be marine were matched to our ocean pixels for use in the model. We used the geographic coordinates of each marine site to bin the number of extrapolated dives into 50 km x 50 km pixels (Supplementary Fig. 15).

From the process of identifying the protection status of each dive site detailed in Section 2.5.1, we can estimate the number of dives made in highly and fully protected MPAs versus those made in areas that offer less protection from fishing or other potentially destructive activities (e.g., seabed mining, drilling) (Supplementary Fig. 16). Globally, we find that 70.15% of marine dives are made in established MPAs of any protection status, 2.93% are made in areas designated as MPAs but not yet implemented or in areas proposed or committed to be future MPAs, and 26.92% are made in unprotected areas (Supplementary Fig. 16). These proportions vary greatly depending on the administering country or territory of marine areas (Supplementary Fig. 17).

2.6.3. Regional, national, and sub-national estimates of the number of scuba dives and divers

Some previous studies have quantified or made reference to the number of divers visiting specific locations or the number of scuba dives made in those places. We again gathered data on the number of dives (and/or divers) for different locations where available from published and gray literature using Web of Science and Google Scholar for comparison with our database (Supplementary Table 6). It is worth noting that global extrapolation from these values is challenging because many reported values only pertain to a select number of sites, a subset of the total activity happening in an area, or are overlapping with other available estimates or reported values. Nonetheless, we summarize these studies here and discuss how these compare to our spatially allocated database of dives described in the previous section. We recognize that historical patterns in diving may not be representative of contemporary trends (i.e., some references are old), but this is the best estimate we can come up with for the current number of diving in a given location given the lack of data on diving worldwide. This exercise is also useful exercise for comparing the magnitudes of our estimates (and considering where they may be biased based on data limitations).

Supplementary Fig. 18 compares the annual number of dives reported in the literature at a national level to our extrapolated estimates where available. In general, our extrapolated estimates were on the same order of magnitude as literature-reported values, with no clear pattern of consistent over- or under-estimation by region. Our extrapolated estimates were lower than those reported in literature more often than they were higher. We attribute the major observed differences to the methods by which the literature estimates were derived (e.g., based on the number of divers and an average number of dives made per diver per year) and the mismatch in time periods associated with some literature estimates and our extrapolation (e.g., the 1980s and 1990s vs. present).

Since many studies reported statistics for diving in specific MPAs, Supplementary Fig. 19 shows the same comparison at the MPA level where available. Again there is no clear pattern of consistent over- or under-estimation by sub-national area, but the variance between literature-reported and extrapolated values does appear to be greater than that of the national level. In this case, our estimates were higher than those reported in literature more often than they were lower than reported values. We attribute much of this to be due to ambiguity in the literature about the area(s) included in their analysis (e.g., whether the reported value actually pertains to diving in the entirety of the MPA, or only in a fraction) and the fact that protected area boundaries have changed over time. For these reasons, we have less confidence in making comparisons at the MPA level but include them here as they may be of interest to some readers.

East Asia & Pacific

Many studies have reported statistics pertaining to the number of scuba divers or dives made in the East Asia and Pacific region. Many of these studies are focused specifically on diving in Australia. Davis and Tisdell[49] make reference to a study specifically looking at dives in the

waters of Queensland, Australia (notably home to the Great Barrier Reef Marine Park), and note that at least 800,000 dives per year (and likely closer to 1 million) were made in Queensland waters in the early 1990s. To corroborate this number, they also point to a separate study reporting 884,000 dives over the course of 12 months between 1989 and 1990 in Queensland waters. Wilks[50] also estimates the number of dives made annually in the Queensland region during the same time period to be approximately 1 million (if not more). Dimmock and Cummins[51] also report on diving in the Great Barrier Reef Marine Park and note that approximately 22,200 divers were traveling to the Park each year by the early 2000s. If we assume each diver makes four dives, this would suggest that approximately 90,000 dives per year were being made in the Great Barrier Reef Marine Park in the early 2000s, corresponding to ~10% of all dives reported for Queensland waters in the other studies. Though not representative of the entire Queensland region, from our spatial extrapolation of the number of dives made in different areas, we estimate that approximately 612,000 dives per year are made in the Great Barrier Reef Marine Park (Supplementary Fig. 19).

An estimate of the total number of dives made in Australian waters each year can be gleaned from statistics pertaining to annual scuba diving fatality rates. In their PhD thesis on incidences of diving fatalities in Australia, Lippmann[52] reports statistics pertaining to the annual scuba diving fatality rates for two groups: Australian residents (0.48 deaths per 100,000 dives or 8.73 deaths per 100,000 divers) and international visitors to Queensland (0.12 deaths per 100,000 dives or 0.46 deaths per 100,000 divers). They also report that an average of 203 annual deaths resulting from compressed gas occurred worldwide between 2010 and 2015, with approximately 3% occurring in Australia (approximately six deaths annually). Based on their calculated fatality rates, we would therefore estimate that 1.25 - 5 million dives were made in Australia each year between 2010 and 2015 (corresponding to 68,728 - 1.3 million divers). We estimate that 1.77 million dives are made in Australian waters per year (Supplementary Fig. 18), which is within the range of dives estimated based on scuba diving fatality rates.

For French Polynesia, Clua *et al.*[31] report that the average number of divers visiting Opunohu was approximately 15,000 each year between 2005 and 2009. Assuming each diver made two to four dives, that would equate to approximately 30,000 - 61,000 dives being made each year. We cannot make a direct comparison because the exact area to which these estimates pertain is unclear, but we estimate that approximately 98,000 dives are made in French Polynesia each year.

For Guam, van Beukering *et al.*[53] provide two estimates of the number of dives made in 2002: the first is based on the number of tanks filled per day and the number of divable days per year, and the second is based on the proportions of local and foreign divers and the number of dives each group makes per year on average. Based on the first method, they estimate that between 256,000 and 340,000 dives were made in the country over the course of a year. The second method yields a wider estimated range of approximately 187,000 - 375,000 dives per year. We estimate that 55,696 dives per year are made in the waters of Guam (Supplementary Fig. 18),

which is lower than both estimates made by van Beukering *et al.*[53] suggesting that our dataset may underestimate the amount of recreational diving that occurs in this country.

For Indonesia, Mazaya *et al.*[54] estimate the number of divers visiting Karimunjawa National Park each year to be approximately 853 based on the total number of annual visitors (13,252) and their primary intent for visiting gleaned from surveys. If we assume each diver makes between two and four dives, this would correspond to 1,700 - 3,500 dives being made in the park each year; we estimate that 5,004 dives are made in the park each year (Supplementary Fig. 19). For the entirety of Indonesia, Mustika *et al.*[55] report that a median of 772,171 divers visit the country each year, which would correspond to between approximately 1.5 and 3 million dives being made annually if we assume each diver makes two to four dives per year. We estimate that 2.23 million dives are made in Indonesia's EEZ each year (Supplementary Fig. 18).

For Malaysia, Hampton *et al.*[56] report that the number of divers staying in Sabah visiting Sipadan and nearby islands is approximately 63,000 annually. They also noted that 120 dive permits were issued each day, and assuming two dives are made per permit per day, 87,600 dives could be made in the area annually. Vianna *et al.*[57] provide a slightly lower estimate of the number of divers visiting Sipadan Island Park each year at 43,900. Nonetheless, the range of potential dives made is slightly higher if we assume each diver makes two to four dives (87,800 - 175,600). We estimate that 184,080 dives are made in Sipadan Island Park each year, which is within that range (Supplementary Fig. 19). Another subnational estimate for Malaysia comes from Teh *et al.*[58], who estimate that between 26,525 and 45,149 divers stayed in Sabah and visited the Semporna Priority Conservation Area in 2013. This would equate to 106,100 - 180,596 dives made if each diver made between two and four dives in the park. The entirety of the Semporna Conservation Area (in which the Sipadan Island Park is located) is not a protected area with established boundaries, and thus we cannot provide a direct comparison, but we estimate that 1.44 million dives are made across the entirety of Malaysia's EEZ each year.

For Palau, Vianna *et al.*[59] estimate that 40,976 divers visited the country in 2010 based on surveys of visitors of different nationalities and their relative rates of participation in scuba diving. If we assume each diver makes between two and four dives, approximately 82,000 - 164,000 dives would have been made in the country that year. We estimate that 96,288 dives are made in Palau's EEZ each year (Supplementary Fig. 18).

For the Philippines, Oracion *et al.*[60] report that more than 22,000 divers visited the Mabini area over the course of a year in the early 1990s. If we assume each diver made two to four dives, this would suggest that approximately 46,000 - 91,500 dives are made in the area each year. The exact area to which this estimate pertains is somewhat unclear, and thus we cannot make a direct comparison, but we estimate that 1.64 million dives are made each year across the entirety of the EEZ of the Philippines.

For Thailand, Wongthong and Harvey[61] report that 130,000 - 150,000 visitors traveled to Koh Tao annually between 2009 and 2011 and that dive tourism is the main attraction on the island. Assuming the average diver makes two to four dives, this would suggest that up to 600,000 dives could have been made in the area annually. Another subnational estimate comes from Tapsuwan and Asafu-Adjaye[62] who estimate that between 8,500 and 17,000 divers visited the Mu Koh Similan Marine National Park in 2003 based on the total number of visitors and their average participation rates in scuba. This would correspond to 17,000 - 68,000 dives being made in the park annually if each diver made between two and four dives. We estimate that 417,000 dives are made in the combined Mu Koh Similan, Mu Koh Surin, and Ao Phang-Nga MPA, and 2.87 million dives are made across all of Thailand each year.

Finally, Depondt and Green[10] surveyed dive operators in seven countries in Southeast Asia: Cambodia, Indonesia, Malaysia, Philippines, Singapore, Thailand, and Vietnam. They found a total of 417 dive operators across those countries. The operators surveyed reported a total of 107,320 visiting divers per year, which is noted to be less than the total number of divers visiting the wider Caribbean, which attracts 57% of all international scuba diving tourists. Assuming an average of three dives per person per year, they estimate that approximately 450,000 dives are made across the seven countries each year (56% of which take place in an MPA according to the operators). Our estimate is quite a bit higher for all seven countries combined: approximately 8.36 million dives per year in the EEZs of Cambodia, Indonesia, Malaysia, Philippines, Singapore, Thailand, and Vietnam.

Europe & Central Asia

Studies on marine diving in Europe and Central Asia are more limited than those for the East Asia and Pacific region, but are available for a few MPAs in the Mediterranean where diving is popular. In France, the Cerbère-Banyuls MPA is popular with divers, and Cerrano *et al.*[48] report that approximately 20,000 dives are made in the MPA each year; our estimate for the same area is 25,016 dives per year (Supplementary Fig. 19). Cerrano *et al.*[48] also report on the Natural Reserve of Bouches de Bonifacio in France, estimating that more than 33,000 dives are made in the MPA annually (compared to our estimate of 23,128 dives per year; Supplementary Fig. 19).

For Italy, Lucrezi *et al.*[63] undertook an analysis of the sustainability of the scuba diving tourism system in two MPAs, one of which is the Portofino MPA in Liguria, Italy. They reference an estimate made by the MPA Authority that between 20,000 and 60,000 scuba dives are made each year in the Portofino MPA, which only encompasses an area of 3.74 km^2. This estimate includes both the 21 established dive sites marked by buoys in the general reserve and in the entire partial reserve area where divers are allowed to dive anywhere with proper authorization. In a later study, Lucrezi *et al.*[64] provide a single estimate of 50,000 dives per year being made in the Portofino MPA. Cerrano *et al.*[48] provide a similar, yet slightly narrower, estimate of 45,000 - 60,000 dives per year for the Portofino MPA. We estimate that 27,376 dives are made in the park each year (Supplementary Fig. 19).

For the Netherlands, Rousseau and Tejerizo[65] estimate the number of annual dives made in Oosterschelde by Dutch and Belgian divers to be approximately 367,100. This area is an estuary and includes both freshwater and saltwater environments. We estimate that 190,216 dives are made each year in the marine portion of Oosterschelde (Supplementary Fig. 19).

For Spain, Rodrigues *et al.*[66] note that 55,647 dives were made in the Medes Islands MPA in 2012. This is similar to the estimate made by Cerrano *et al.*[48], that 60,000 - 70,000 dives are made in the area annually. For comparison, we estimate that 86,848 dives are made in the Medes Islands MPA each year (Supplementary Fig. 19). Cerrano *et al.*[48] also report on the Serra Gelada Marine Park in Spain, estimating that more than 24,000 dives are made annually (compared to our estimate of 9,440 dives per year for the same area; Supplementary Fig. 19).

For Turkey, Albayrak *et al.*[67] estimate that between 20,000 and 60,000 dives were made in the Kemer region in 2017. Their lower estimate only includes dives made by certified divers, while the higher estimate includes discovery or “resort” dives. The exact area to which this estimate pertains is somewhat unclear so we cannot provide a direct comparison, but we estimate that nearly 114,000 dives are made in the EEZ of Turkey per year.

Finally, on a broader scale, Cerrano *et al.*[48] estimate that more than 200,000 dives are made annually in 11 MPAs in southern Spain, southern France, and Italy. Across the entire Mediterranean, they estimate that nearly a million dives are made each year in 47 different MPAs that are frequented by divers.

Latin America & Caribbean

There are many available statistics relating to diving in Latin America and the Caribbean. For the Bahamas, Haas *et al.*[68] estimate that more than 314,000 dives were in the country between 2014 and 2015 based on a survey of divers and dive operators. Our estimate for the country is slightly lower at 229,864 dives per year (Supplementary Fig. 18).

For Barbados, Schuhmann *et al.*[69] estimated that between 30,000 and 50,000 divers visited the country between 2007 and 2008, which would correspond to approximately 60,000 - 200,000 dives being made each year if we assume each diver made two to four dives. Our estimate is slightly lower at approximately 50,000 dives made annually across Barbados’ EEZ (Supplementary Fig. 18).

For Belize, Hawkins *et al.*[70] report that 30,000 divers visited the Hol Chan Marine Reserve in 1990. Though potentially outdated, if the same number of divers still visit the park each year and make between two and four dives, up to 120,000 dives could be made in the area annually. We only estimate that 7,552 dives are made in the reserve each year (Supplementary Fig. 19).

Access fee programs exist for Bonaire, Sint Eustatius, and Saba from which it is possible to determine the number of divers who have visited the area. Parsons and Thur[71] determine that 28,000 tags were purchased by divers to dive in the Bonaire National Marine Park in 2001. In practice, each tag grants its holder the right to make unlimited dives in the MPA over the course of the year so they were unable to directly discern the total number of dives made, but if we assume that the average diver made two to four dives, this would suggest that 56,000 to 112,000 dives could have been made in the MPA that year. Our estimate is higher at approximately 204,848 dives made per year in the Bonaire National Marine Park, but this could be a result of the increase in diving popularity over the last 20 years. Hawkins *et al.*[70] estimate that 12,460 to 60,564 dives were made in the Saba Marine Park annually between 1993 and 2002 based on the average dives per site and the number of sites. Such an estimate was only possible because all sites are buoyed, only one dive boat is allowed per buoy at a time, and divers are not allowed to enter from shore. Our estimate for Saba is lower at 12,272 dives per year (Supplementary Fig. 19).

For Brazil, Pires *et al.*[72] report that the number of “diving operations” made in the Fernando de Noronha Archipelago in one year was 24,000. It is unclear whether this is the number of divers visiting the area, the number of dive trips, or the number of dives. We estimate that 59,000 dives are made in the park each year (Supplementary Fig. 19).

For Colombia, Trujillo *et al.*[73] report that 4,200 divers visit Los Corales del Rosario y San Bernardo annually, which would correspond to 8,400 to 16,800 dives made each year if each diver makes between two and four dives. Our estimate of 15,104 dives per year is within this range (Supplementary Fig. 19).

For Mexico, in their study on the dive industry, Arcos-Aguilar *et al.*[34] asked operators about the number of scuba (and snorkeling) clientele served each year. Based on survey responses, they estimated that the total number of scuba divers tended by all active operators per year in Mexico is between 1.33 and 1.70 million. If we assume each diver makes two to four dives, that would correspond to 2.66 to 6.80 million dives being made in Mexico each year. We only estimate that 1.28 million dives are made in Mexico’s EEZ each year (Supplementary Fig. 18), but given the popularity of cave diving in Mexico—which usually occurs inland in freshwater cenotes, and is often technical diving and thus wouldn’t be represented in our dataset—this figure likely isn’t comparable. If we include dives occurring in freshwater, our total estimate for Mexico (freshwater and marine) increases to just over 2 million dives per year.

For the Turks and Caicos Islands, Rudd[74] estimates that 150,000 dives are made annually across all dive sites in the country. Our estimate is similar at 134,048 dives per year across the entirety of the Turks and Caicos EEZ (Supplementary Fig. 18).

Finally, Green and Donnelly[75] surveyed 138 dive operators in 30 countries across the Wider Caribbean and Pacific Coast of Central America. They estimate that they surveyed 22% of the total number of operators in the region (not including the United States). They note that there

are at least 250 more operators in Florida that are active in the region, but they excluded these from their study to avoid bias associated with the disproportionate representation of U.S. operators. The surveyed operators are reported to serve 750,000 divers each year, with an average of four dives being made per person per year. By extrapolating across all operators, they estimate that a total of 15 million dives per year are made in the 30 countries they surveyed. Countries surveyed were Anguilla, Antigua and Barbuda, Bahamas, Barbados, Belize, Bermuda, British Virgin Islands, Cayman Islands, Colombia, Costa Rica, Dominica, Dominican Republic, El Salvador, Grenada, Guadeloupe, Honduras, Jamaica, Martinique, Mexico, Montserrat, Netherlands Antilles, Panama, Puerto Rico, St. Barthelemy, St. Lucia, St. Vincent & the Grenadines, Trinidad and Tobago, Turks and Caicos, U.S. Virgin Islands, and Venezuela. For the same subset of countries, we estimate that 4.67 million marine dives are made each year. This could suggest that our method of allocating dives underestimates the total number of dives made across the Caribbean region each year.

Middle East & North Africa

Studies on scuba diving in the Middle East and North Africa region are limited, but Cesar[76] conducted an analysis of scuba diving in Egypt in 2000 and estimates that more than 500,000 divers (both foreign and domestic) are active in the country each year. They also estimate that more than 4.1 million dives were made in the country across both the Red Sea and Gulf of Aqaba in 2000. Our estimate for Egypt is lower at 2.87 million dives annually (Supplementary Fig. 18).

North America

For the United States, Buzzacott *et al.*[77] report extrapolations pertaining to the number of dives made in the United States based on the SFIA statistics referenced in Section 2.6.1. They estimate that Americans made nearly 26 million dives in 2015. Oh *et al.*[78] estimate that between 23 and 46 million dives are made by Americans each year. Both studies are ambiguous as to whether or not the reported statistics pertain to the number of dives made by Americans in the United States or anywhere in the world; we assume it to be the latter based on other studies. For instance, DEMA reports that the state of California ranks highest in the United States in terms of the number of new diver certifications issued in 2021 (~14% in 2021, ref. [44]) and reports that approximately 1.38 million dives are made in California each year (though this includes both freshwater and marine diving). The same statistic for the number of dives is reported by Pendleton and Rooke[79]. If we assume California makes up the same proportion of new diver certifications as total dives, that would suggest that approximately 9.86 million dives are made across the United States annually.

In another state-level report, Northern Economics, Inc.[80] estimates that almost 2.2 million dives were made in public waters in the state of Washington in 2014, but this figure also includes freshwater diving. We only estimate that approximately 2.8 million marine dives are made in the United States each year. However, our estimate for the United States increases to approximately 7.5 million dives if we include freshwater diving.

Ladd *et al.*[81] undertook a comprehensive study of diving in British Columbia, Canada and estimated that 125,392 dives were made in the province in 1999 based on the number of tank fills reported by all dive shops and charter operators. We don't have a direct comparison for this statistic, but we estimate that only 102,424 marine dives are made across Canada each year. The total for Canada increases to approximately 595,000 dives per year if we include freshwater diving.

South Asia

Zimmerhackel *et al.*[82] report the total number of dive visitors to the Maldives to be just over 77,000 in 2016. They also report that the average dive tourist stays for 6.6 dive days and makes one to two dives per dive day, which would suggest that approximately 0.5 - 1 million dives are made each year. It is noted that these statistics pertain specifically to the shark diving industry in the country and therefore are not representative of all diving in the country. We estimate that 1.27 million dives are made in the EEZ of the Maldives each year, which is very comparable (Supplementary Fig. 18).

Sub-Saharan Africa

There are few statistics available on recreational diving in Sub-Saharan Africa. Lucrezi *et al.*[63] studied the Ponta do Ouro Partial Marine Reserve (PPMR) in southern Mozambique. For the Ponta do Ouro PPMR—which encompasses an area of 678 km^2—they were unable to provide an estimate of the number of dives for the entire area, but note that between 21,000 and 30,000 scuba dives are made each year from the main launching site on both commercial and private vessels. The same estimate of 30,000 dives is also reported by a later study[64]. Our estimate for the Ponta do Ouro PPMR is lower at 16,520 dives per year (Supplementary Fig. 19).

For South Africa, a number of studies have looked at diving activity in Sodwana Bay. Celliers and Schleyer[83] studied coral community structure on the high-latitude reefs in Sodwana Bay, South Africa. Of the reefs in South Africa, those in Sodwana Bay are part of the central reef complex (as opposed to the northern or southern complexes), which is the hub of recreational diving in South Africa. The southern reefs have sanctuary status and therefore should theoretically have no usage, and the northern reefs are isolated from tourist activities. They note the fact that scuba diving in Sodwana Bay has been popular since the 1980s and 1990s, with activity peaking at around 120,000 recreational dives per year in 1996 (corresponding to 240,000 - 480,000 dives if we assume the average diver made two to four dives). It is noted that this peak corresponds to the first signs of quantifiable damage to coral communities, ultimately resulting in the development of recommendations on sustainable diving limits (implying that a decrease in the annual number of dives has likely resulted).

Saayman and Saayman[84] corroborate that the number of divers visiting Sodwana Bay has decreased since the peak in 1996 and has likely been between 60,000 and 80,000 divers per year since 2005. Based on the characteristics of the average diver, this could mean that only

120,000 to 320,000 dives are made in Sodwana Bay each year. Dicken[85] and Schoeman *et al.*[86] both provide an even lower estimate of approximately 59,000 dives being made annually in Sodwana Bay. While we cannot provide a comparison for Sodwana Bay alone, we estimate that approximately 507,000 dives are made across the EEZ of South Africa each year.

Antarctica

Lamers and Gelter[87] estimate that between 10,000 and 11,000 dive tourists visit Antarctica each year. It is unclear whether the average visitor makes more than one dive, but the number of dives made each year would therefore likely be greater than 11,000. Our database does include an estimate for the number of dives made in Antarctica, but we only estimate that 1,888 marine dives are made across the entirety of the continent each year, suggesting that this area may be underrepresented in our database.

2.7. Factors affecting demand for diving

There is evidence that improvement in the biological condition of a dive site increases demand for diving at that site[88,89]. The increase in demand can be modeled as a horizontal or vertical shift in the demand curve. We use willingness-to-pay (WTP) studies to quantify the vertical shift in the demand curve. We parameterized three factors known to shift the demand curve. First, the MPA name effect: divers are willing to pay more to dive inside an area designated as an MPA as compared to an area that is not an MPA, all else being equal (i.e., controlling for differences in habitat and biodiversity)[2,3]. Second, the biomass (or abundance) effect: divers are willing to pay more to dive in areas with higher fish biomass (or abundance) or a higher proportion of bigger fishes. Third, the biodiversity effect: divers are willing to pay more to dive in areas with a greater diversity of species. Improvements in habitat quality due to improved area protection could also lead to a higher WTP, but we did not include this factor given our lack of general understanding about the magnitude of benefits MPAs provide to habitat maintenance and recovery. Furthermore, it has been shown that dive tourists respond more to changes in fish attributes (e.g., variety of fishes, abundance of fishes, big fishes) than habitat-based attributes[90].

We gathered data on diver WTP from published literature. We collected publications from the Web of Science and Connected Papers databases. We did not use Google Scholar for this search because test results were either irrelevant or redundant. We used the following search terms in Web of Science: “(((ALL=(MPA or marine protected areas)) AND ALL=(time*))) AND ALL=(WTP or willingness to pay)” or "WTP AND tourism". We used Connected Papers to check for papers that were connected to Grafeld *et al.*[13]. Finally, we collected more papers by looking at citation trails of publications that were found via Web of Science or Connected Papers. Citation trails were the most useful method of finding relevant literature. Citation trails for literature reviews published by Thur[91] and Peters and Hawkins[92] were especially useful in finding literature for the name effect parameter.

We initially collected 54 papers. Upon reading them, we found that 22 papers included relevant data, 12 papers did not include relevant data but included useful contextual information, and 18 were not relevant.

We conducted data collection between June 2021 and September 2021. The papers cited were published between the years 2000 and 2020. Data were then manually inputted into a data tracking spreadsheet in which each tab contained data for a different parameter in the tourism model. We used this data to parameterize MPA name effect, biomass, abundance, and biological diversity; all three of which are known to shift scuba divers' demand curve. We recorded as much of the following information as possible for all relevant studies:

- Country
- Marine park or location
- Year the park was established
- Year the study was conducted
- Change in parameter
- Change in diver demand
- WTP
- Units
- Standard deviation and/or error
- 95% CI (Lower Bound)
- 95% CI (Upper Bound)
- Whether the WTP value is absolute or marginal
- Base value(s)
- Method
- Citation

Since some studies report absolute WTP (i.e., the diver's total WTP including the base cost of diving) and others report marginal WTP (i.e., the change in the diver's WTP relative to the base cost of diving), we converted all reported values into marginal WTP for consistency and calculated the percent change in WTP relative to the base cost.

2.7.1. MPA name effect

Few studies have explicitly tried to quantify the MPA name effect on WTP, and thus, we must consider other similar effects. For example, a common attribute of fully protected MPAs is that they explicitly exclude fishing activities within their borders, and divers are known to be willing to pay to avoid encountering fishing gear[93]. Regardless of whether the designation of the MPA changes fishing patterns, this could be a perceived difference for divers.

To parameterize the effect of designating an area as an MPA (the "name effect") on the shift in demand for diving, we rely on the findings of Arin and Kramer[94]. They assessed the WTP of divers to dive in a hypothetical MPA by asking divers, "How much would you be willing to pay as a daily, per person entrance fee to a marine sanctuary where fishing is prohibited, in addition to the other costs of the trip?" Their findings suggest an average WTP of US$4.20 for a two-tank dive (or US$2.10 per dive), which is a lower estimate than that obtained from similar studies (Supplementary Table 7).

For comparison, Tongson and Dygico[95] found a WTP of US$41.11 per multi-day trip for divers to enter the Tubbataha MPA in the Philippines (US$13.70 per diving day assuming the average of three diving days per trip reported by the authors). Another reference point comes from Gill *et*

al.[93]. Though not directly estimating WTP to dive in an MPA, they studied WTP values of divers to avoid encounters with fishing activity or fishing gear while diving (which is a function that would be expected from no-take MPAs). They report WTP values of US$91.31 in St. Kitts and Nevis, US$67.99 in Honduras, and US$114.04 in Barbados for two-tank dives (US$45.65, US$34.00, and US$57.02 per dive respectively).

Given the limited availability of studies quantifying the MPA name effect, we chose to use the most conservative estimate of US$2.10 per dive in our model. This value is consistent with existing user fees for diving in MPAs in the Indian and Pacific Oceans (median value of US$5.00). Nonetheless, it is worth noting that Depondt and Green[10] suggest this to be set too low to realistically capture the benefits of MPAs.

2.7.2. Biomass, abundance, and size effects

The effects of increased biomass, abundance, or a greater number of large individuals are better quantified in the literature. To parameterize the effect of increased fish biomass and related metrics on the shift in demand for diving, we rely on the findings of Grafeld *et al.*[13] and Gill *et al.*[93] (Supplementary Table 8). For comparison, we also present findings from other studies pertaining to changes in WTP for increases in biomass and abundance of charismatic species (i.e., sharks, sea turtles, goliath groupers) and find similar magnitudes of the effect (Supplementary Table 9).

Grafeld *et al.*[13] found that an increase in biomass from low density (< 25 g/m^2) to medium density (25–60 g/m^2) results in a marginal WTP of US$8.34 (an increase of 8.34% relative to the base dive price of US$100). They also found that a further biomass increase from medium (25–60 g/m^2) to high (>60 g/m^2) results in additional WTP of US$13.48. In total, an increase in biomass from low to high could generate a marginal WTP of US$21.82 (an increase of 21.82% relative to the base dive price).

Gill *et al.*[93] found an average marginal WTP of US$83.64 (an increase of 89% relative to the base dive price) for fish abundance to increase from moderate (17 individuals per 50 m^2) to many (25 individuals per 50 m^2). They also found that an increase from low (6 individuals per 50 m^2) to many (25 individuals per 50 m^2) more than doubles this WTP value[93].

As some divers might be more interested in seeing particularly large or noteworthy fish, it's also worth looking at how the demand for diving might respond to changes in biomass (or abundance) of only large fish. Gill *et al.*[93] found an average marginal WTP of US$65.99 (an increase of 70% relative to the base dive price) for dive experiences with sightings of few large individuals (1% - 10%) to many (>50%). They also found that WTP more than doubles when going from zero large fish sightings to many (>50%).

Overall, across both studies, an increase from the base conditions to the biggest improvements in biomass, abundance, or proportion of large fish corresponds to an average increase in WTP

of 83.86% relative to the base dive price. We conservatively use this value to characterize the maximum increase possible in WTP arising from increases in biomass from our model. That is, this increase in WTP is only seen for the pixel yielding the greatest increase in biomass when all sites are protected in MPAs. We assume that the WTP for all other pixels is linearly scaled based on their resultant biomass increase relative to the maximum (i.e., a site with half the biomass increase relative to the site with the largest biomass increase from protection will have a marginal WTP of 41.93% of the base dive price).

2.7.3. Species diversity effect

The effects of increased species diversity on diver WTP are also fairly well quantified in the literature (Supplementary Table 10). Grafeld *et al.*[13] found that an increase in species diversity from low (two fish species/m^2) to medium (four fish species/m^2) in Guam resulted in a marginal increase in WTP of US$10.10 (an increase of 10.10% relative to the base dive price of US$100). They also found that a further increase in species diversity from medium (four fish species/m^2) to high (eight fish species/m^2) results in an additional marginal increase in WTP of US$13.33. In total, an increase in species diversity from low to high could generate a marginal increase in WTP of US$23.43 (an increase of 23.43% relative to the base dive price).

Schuhmann *et al.*[35] also used a choice experiment to quantify this effect and found that an increase in species diversity from low (five or fewer fish species) to high (25 or more fish species) in Barbados resulted in a marginal increase in WTP ranging from US$50.89 to US$109.65 depending on the model applied (an increase of 67.9% - 146.2% relative to the base price for a two-tank dive of US$75).

Similar to Schuhmann *et al.*[35], Cazabon-Mannette *et al.*[29] used a choice experiment to elicit diver WTP for species diversity and analyzed their results using a variety of models. They found that an increase in species diversity from low (five or fewer fish species) to high (25 or more fish species) in Tobago resulted in a marginal increase in WTP ranging between US$29.21 and US$40.45 (an increase of 58.4% - 80.9% relative to the base price for a two-tank dive of US$50).

Overall, across all three studies, an increase from the base condition to the biggest improvement in species diversity corresponds to an average increase in WTP of 81.86% relative to the base dive price. In the same way as with the biomass effect, we conservatively use this value to characterize the maximum increase possible in WTP arising from increases in biodiversity score from our model.

Though not directly incorporated into this analysis, we recognize that the presence of charismatic species such as sharks, sea turtles, and goliath groupers enhances the experience for many divers and adds to divers' WTP and demand for diving[13,29,35,88]. For instance, divers were found to be willing to pay more than US$62 (in addition to the base dive price) to see one turtle in Tobago. The addition of a further one or more turtles increased WTP by US$20 -

US$40[29]. Increases in divers' WTP for shark and sea turtles (see Supplementary Table 9) are of comparable magnitude to the effect of encountering high species richness (Supplementary Table 10). While some charismatic species, such as sharks and turtles, are incorporated in our model, there are many other species that divers care about (e.g., goliath groupers, Napoleon wrasse, frogfishes, seahorses, schools of jacks, etc.)[13,28] that are not as they tend not to be exploited by commercial fisheries. We therefore use the WTP estimates corresponding to changes in species richness to parameterize changes in demand for diving as a result of changes in biodiversity resulting from implementation of MPAs. We recognize that the average maximum willingness to pay for biodiversity improvements can be generated in Tobago, Guam, Barbados (as described in the studies presented in Supplementary Table 10), or in any parts of the world. It is less likely that an improvement in biodiversity in an MPA in India would negatively affect the WTP for diving in an MPA in Guam, although it is possible that this may happen within a smaller geographical area (such as within a country). We therefore normalize the biodiversity component of WTP for diving per EEZ area (defined by administering territory per Marine Regions) such that protecting the best dive site in a country's EEZ leads to the highest WTP for biodiversity at that site. We relaxed this assumption in our sensitivity analysis where we also tested the effect of normalizing per sovereign state and per region (see Section 3).

2.8. Diver origins

We gathered data on diver origins from published literature to estimate the global distribution of consumer benefits associated with dive tourism. We collected this information whenever available from publications quantifying the number of divers visiting specific locations or the number of scuba dives made in those places (see Section 2.6.3) and studies quantifying diver WTP (see Section 2.7).

Diver origin was recorded to the highest resolution reported in the study (i.e., "United States" instead of "North America"), but we categorized all diver origins for comparison across studies. Whenever possible, we categorized origin as "Domestic" or "Foreign" relative to the location in which the diving activity was occurring. In some cases, diver origin was reported in a way where domestic divers were lumped in with divers from nearby countries (e.g., diver origin reported as "North America" for diving activity occurring in the United States) or where the origin of divers from adjacent countries was distinguished from those traveling from further away (e.g., diver origin reported as "Europe" or "United States" for diving activity occurring in Spain). In both of these cases, we categorized the origin as "Regional". There were also some cases in which origin was not reported for some percentage of divers or was reported as "Other". We categorized origin as "Unknown" in these cases (Supplementary Fig. 20).

In order to estimate the distribution of benefits, we calculated the average percentage of divers that were domestic versus foreign by region (Supplementary Table 14). Studies where some percentage of divers were classified as being of "Regional" origin were only included in this calculation if all regional divers could be identified as being of foreign origin (e.g., diver origin reported as "Spain" OR "Europe" for diving activity occurring in Spain). Studies where domestic

divers were included with those of adjacent countries were not included (e.g., diver origin only reported as “Europe” for diving activity occurring in Spain), nor were those where the origin of some divers was unknown (Supplementary Table 14).

2.9. MPA management costs

The values used for estimating the management costs associated with protecting an additional 1% of the global ocean referenced in the main text were sourced from Balmford *et al.*[96] and Cullis-Suzuki and Pauly[97] (Supplementary Table 12).

3. Sensitivity analysis

We test the sensitivity of our reported maximum revenue that can be generated from combining dive fee and MPA to alternative parameter values. In particular, we run our model using the lower and upper bound of our estimated yearly global number of dives, alternative assumptions about the price per dive parameterization, and alternative weighting of biodiversity scores.

3.1. Quantity of dives

We estimate 50.7 million scuba dives are made worldwide each year (lower bound = 26.2 million, upper bound = 82.7 million), with 65.3% of those – or 33.1 million dives (lower bound = 17.1 million, upper bound = 54.0 million) – being made in the ocean. In our base model, we used 33.1 million dives as the global number of dives, with an uncertainty bound generated by randomly selecting a global dive value from a uniform distribution with a lower and upper limit of 17.1 million and 54.0 million, respectively. Using the lower and upper bound estimates of yearly global dive numbers, we estimate that the maximum revenue that can be generated from combining dive fees and MPA ranges from US$1.19 billion to US$3.76 billion (Supplementary Table 13).

3.2. Price per dive

In parameterizing the dive price, we used a global median value of US$58.75 per dive. We explore two other scenarios of parameterizing dive prices in our sensitivity analysis model. The first alternative scenario is where the median dive price per country is uniformly applied to all ocean pixels falling under that country’s jurisdiction. Ocean pixels corresponding to countries without data on dive prices receive the median price for the region. The second alternative scenario is where dive prices are interpolated for all pixels based on the median price per dive per pixel. The maximum revenue that can be generated from combining dive fee and MPA that results from the first and second alternative scenarios are US$2.30 billion and US$2.27 billion, respectively (Supplementary Table 13).

3.3. Biodiversity parameterization

Our synthesis shows that divers are willing to pay up to 84% more for biomass improvements (Supplementary Table 8). We use these empirical estimates to parameterize the magnitude of the diver's willingness to pay as a function of model-derived changes in biodiversity from MPAs, where willingness to pay is higher for bigger changes in biodiversity. We use the changes in our model-derived biodiversity scores as our metric for biodiversity changes in response to MPA (see Sections 1.6 and 2.3).

We recognize that this maximum willingness to pay for biodiversity improvements can be simultaneously achieved in any part of the world, i.e., biodiversity improvements from MPA in Hawaii and India can increase WTP in both places. Furthermore, we recognize that dive sites within a geographic or political boundary may interact – improvements in dive sites in one area can affect WTP and the demand for diving in adjacent areas. Therefore, we normalize the biodiversity score improvements from MPA using three different geographic/political boundaries: normalization of biodiversity values by territory (e.g., Hawaii is separate from the U.S. Mainland), sovereignty, and region. We use the territory partitioning as our primary model output, with the results of the other scenarios reported in the sensitivity analysis below. Partitioning by sovereignty has minimal effect on our results, while implementing the normalization by region reduced dive tourism benefits from MPAs. We note that normalization by region is less realistic as it means that an MPA in Australia that improves the WTP of divers diving in Australia negatively affects diver's WTP for biodiversity in all of East Asia and the Pacific region (i.e., the Philippines, Indonesia, Hawaii, Guam, etc.). Most importantly, normalizing per region unrealistically reduces the contribution of biodiversity to the dive tourism benefits from MPAs, i.e., the contribution of biodiversity changes from 49.3% of the dive tourism benefit from MPAs, as shown in Fig. 3A to 13.9% (with biomass improvements becoming responsible for 79.4% of the dive tourism benefits from MPAs) (Supplementary Fig. 22).

Supplementary Figures

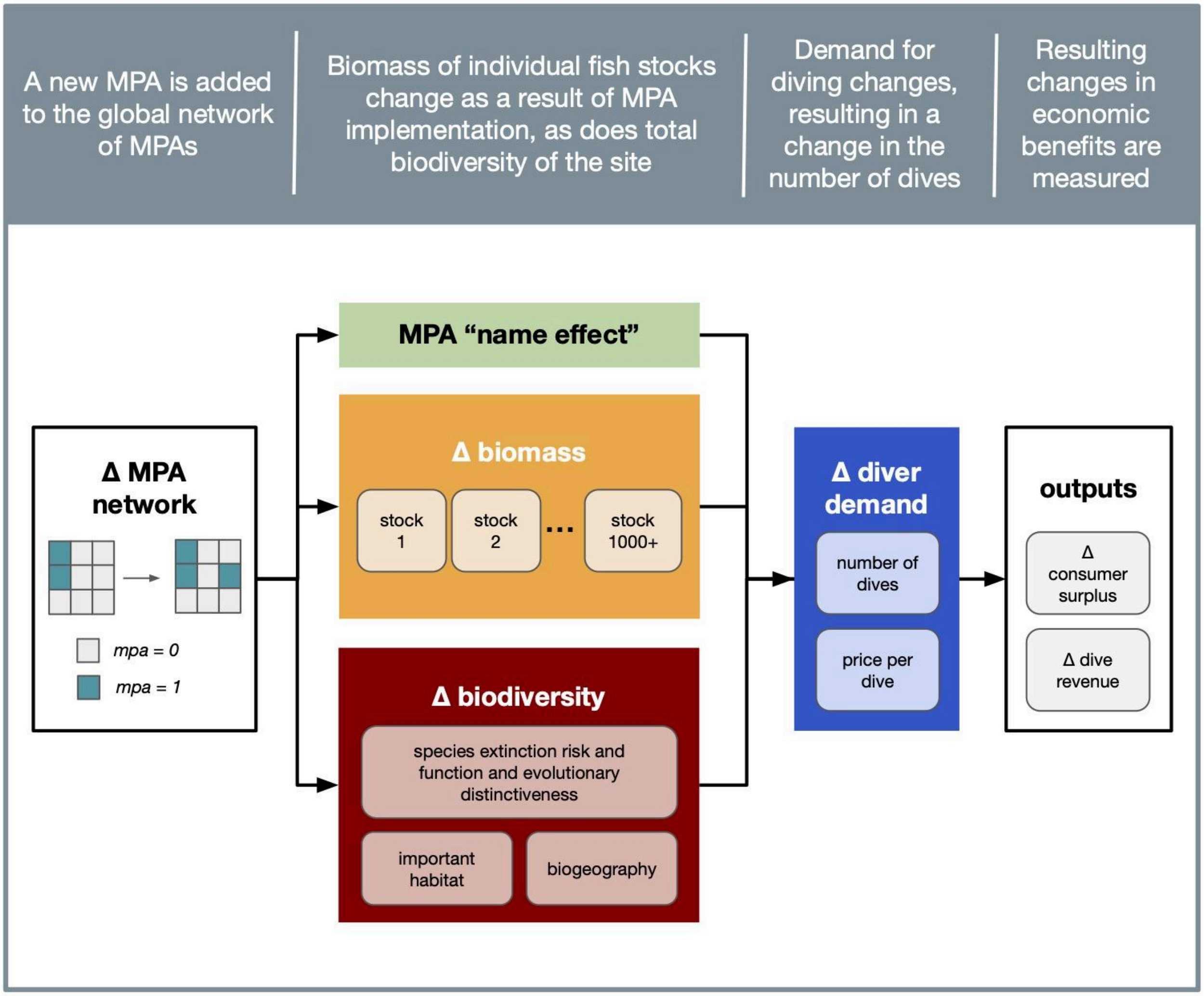

Supplementary Fig. 1 | Conceptual framework for how marine protected areas (MPAs) generate tourism benefits. Establishing MPAs improves fish biomass and species diversity. These, together with the MPA name, improve the demand for diving. An improvement in dive demand affects consumer surplus, producer surplus, and dive revenue. The delta symbol (Δ) indicates a change in the parameter listed.

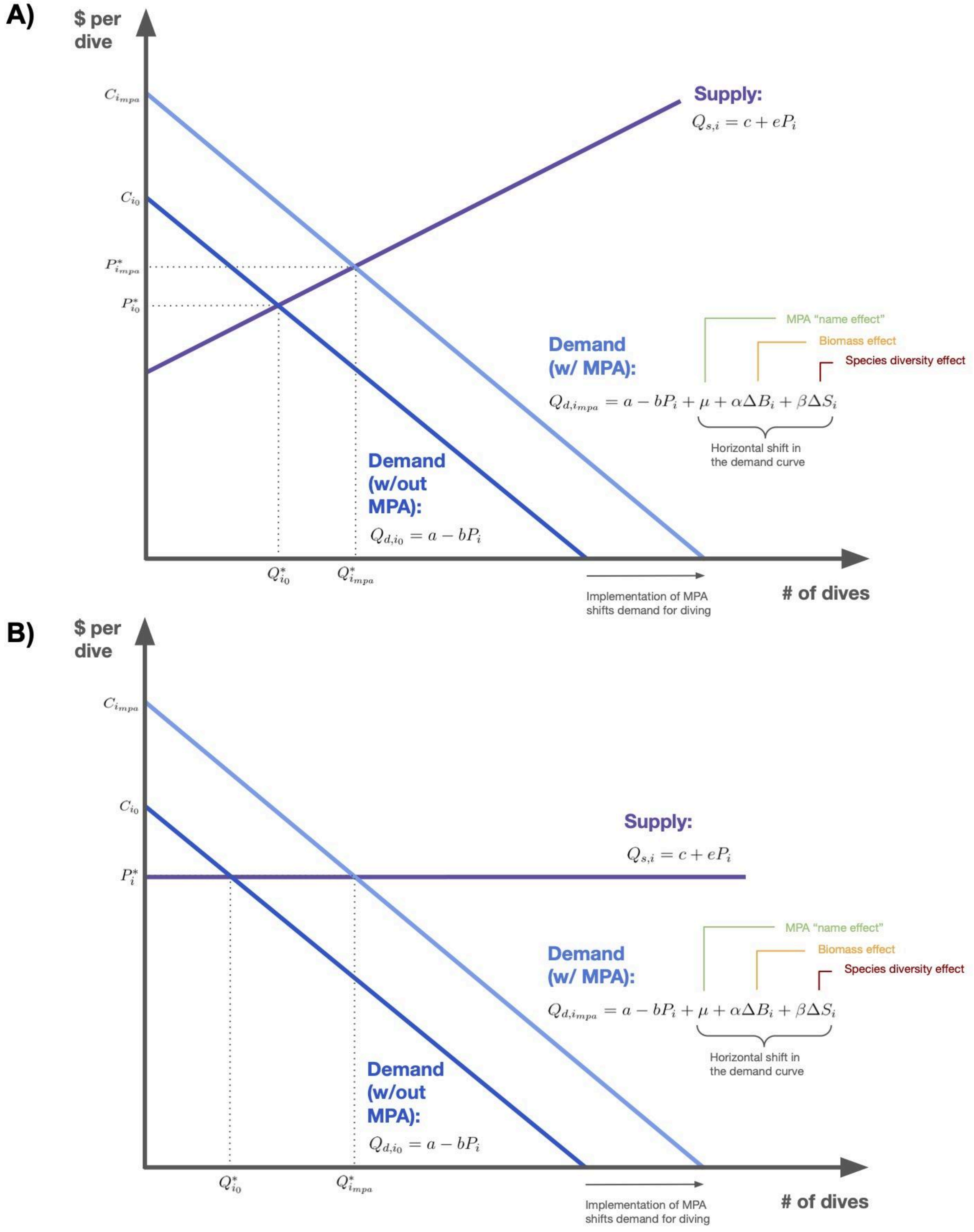

Supplementary Fig. 2 | Illustration of a shift in demand for diving driven by the implementation of an MPA. Panel A depicts a system where the supply curve is upward-sloping and panel B depicts a system where the supply curve is flat.

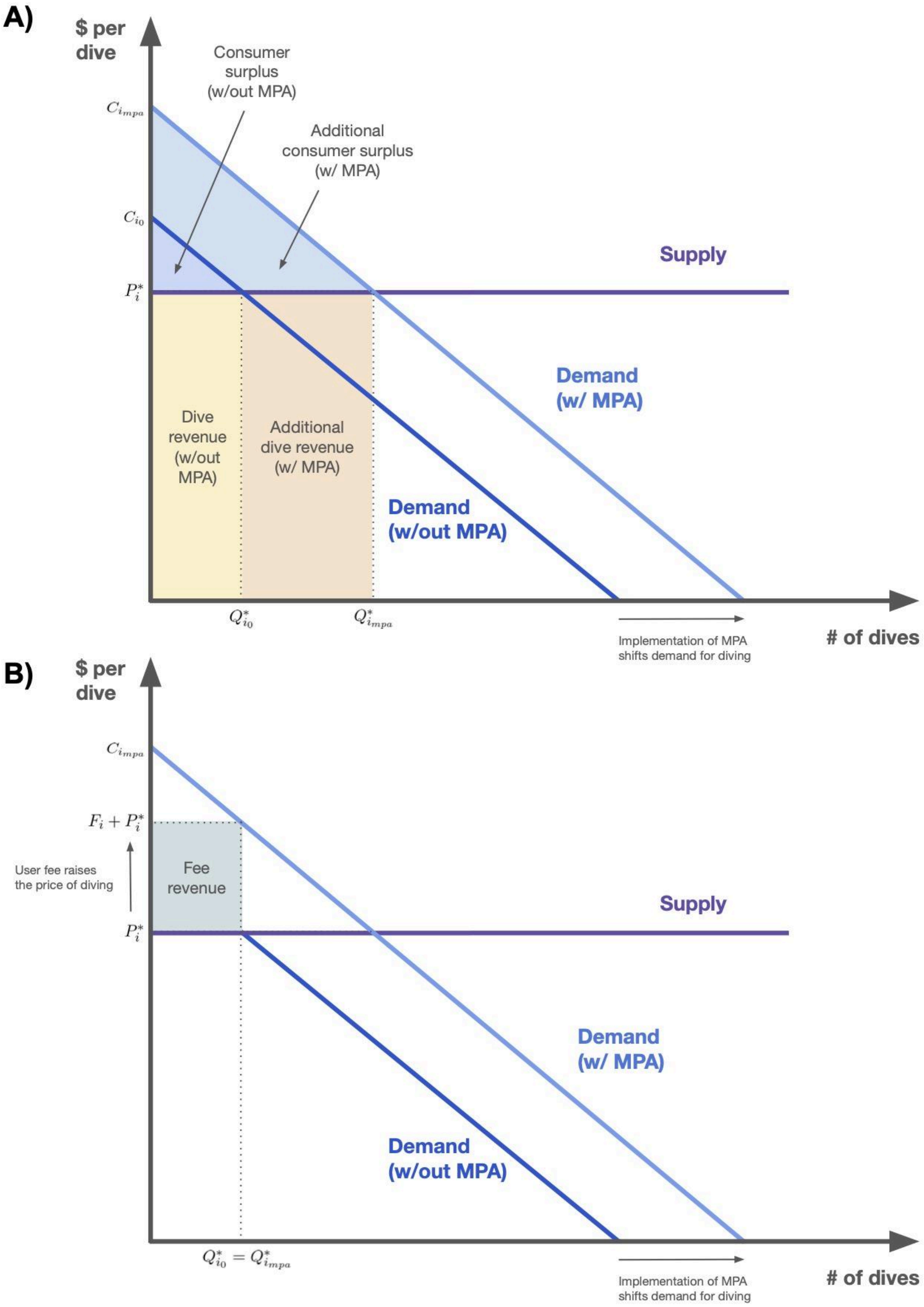

Supplementary Fig. 3 | Illustration of benefits driven by the implementation of an MPA. Placing an MPA in unprotected dive sites is expected to shift the demand curve to the right (or upward). Panel A depicts resulting changes in consumer surplus and dive operator revenue due to MPA. Panel B depicts resulting changes in dive fee revenue due to imposing an MPA dive fee of F_i. When a dive fee is imposed, the demand curve shifts to the left (or downward). In our example, implementing an MPA and imposing a dive fee of F_i holds visitation rate constant, while generating a fee revenue equivalent to the shaded area marked as “Fee revenue” in the figure.

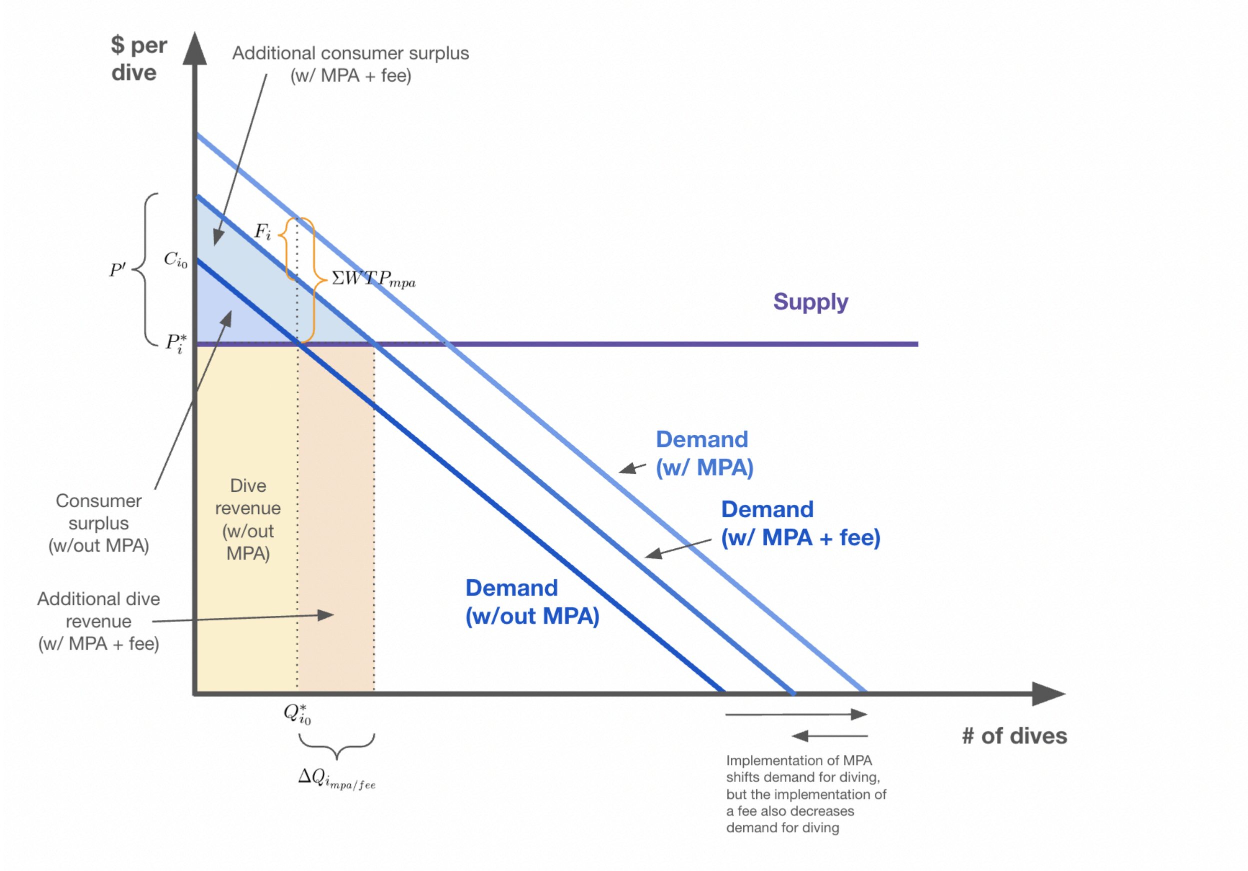

Supplementary Fig. 4 | Illustration of benefits driven by the implementation of an MPA and a fee for diving. Placing an MPA in unprotected dive sites is expected to shift the demand curve to the right (or upward). However, when a dive fee is imposed, the demand curve shifts back to the left (or downward). Changes in consumer surplus and dive operator revenue due to the implementation of an MPA and a fee are depicted by shaded areas.

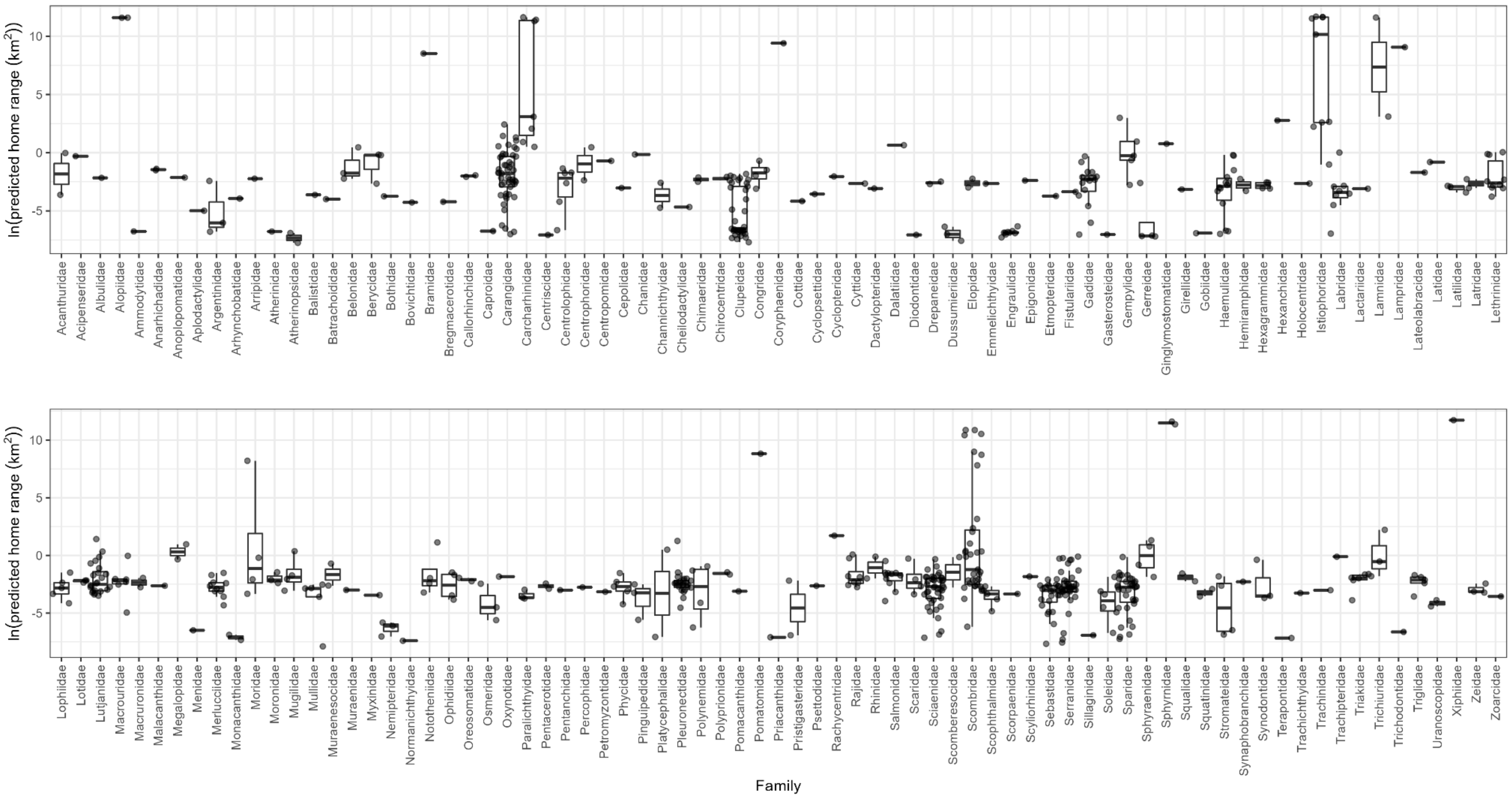

Supplementary Fig. 5 | Predicted homerange values (natural log of km^2) of marine fish species (N = 667) by family. Families are listed alphabetically and split between the two panels for clarity.

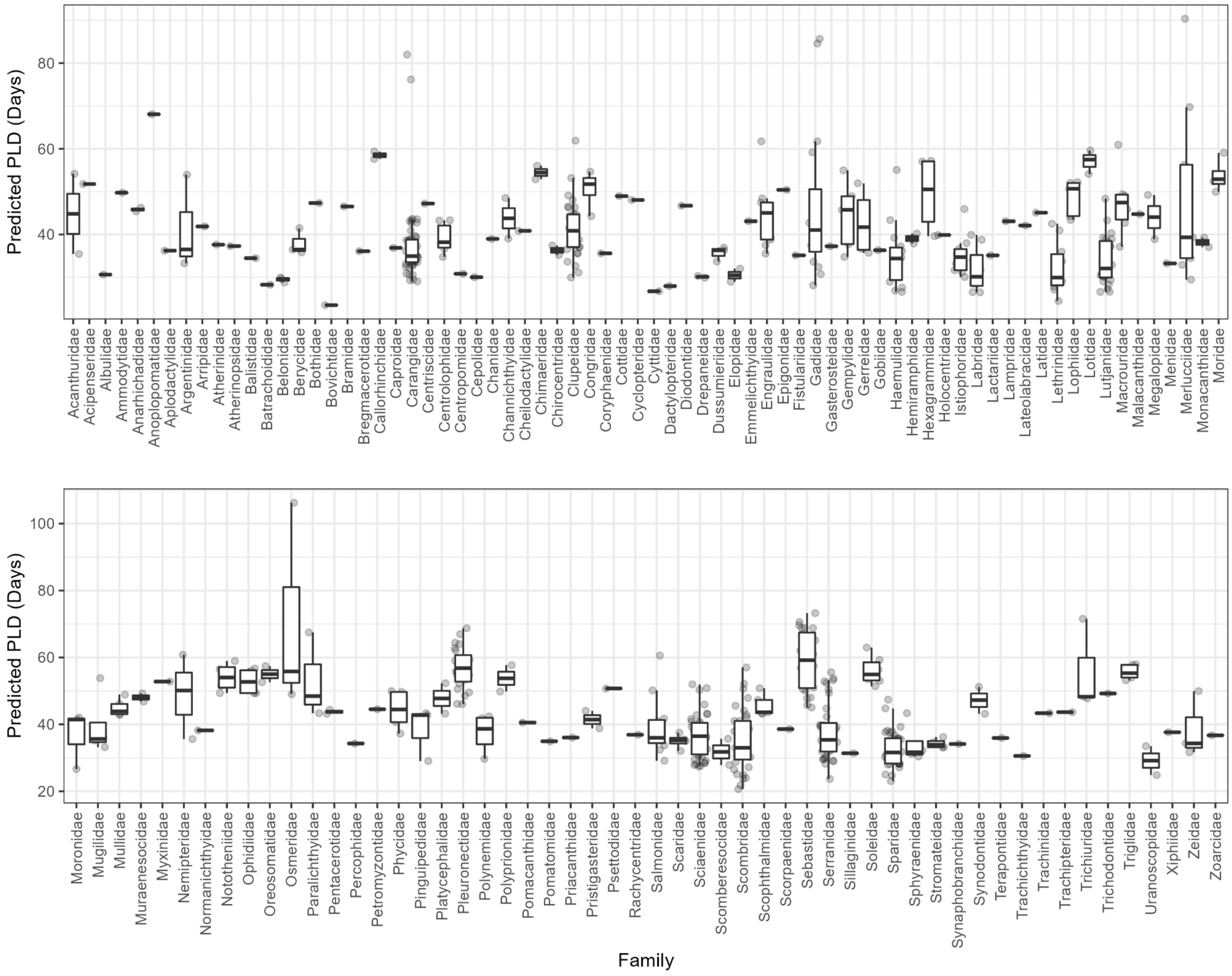

Supplementary Fig. 6 | Predicted pelagic larval duration (days) of marine fish species (N = 610) by family. Families are listed alphabetically and split between the two panels for clarity.

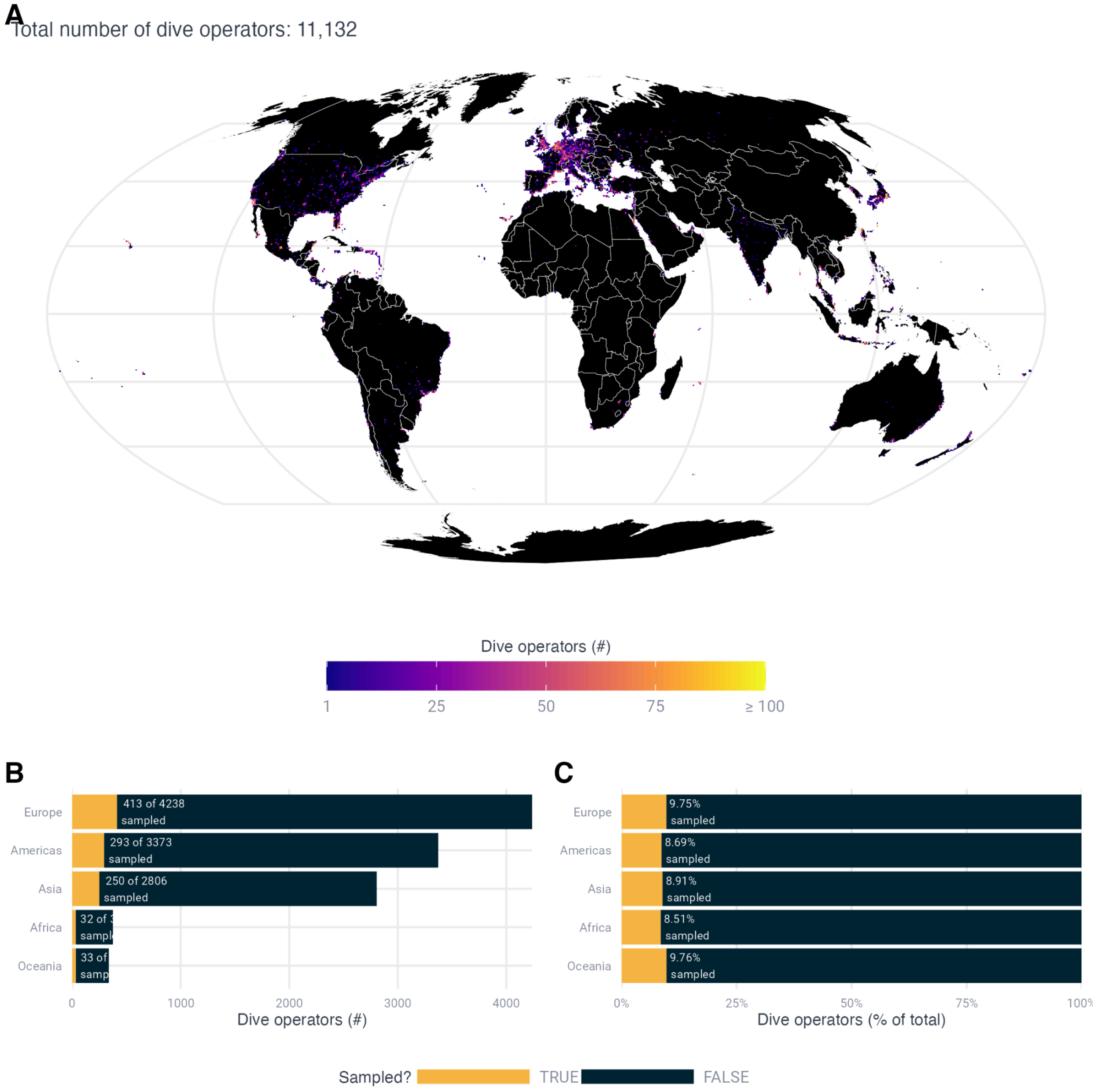

Supplementary Fig. 7 | Locations of and sampling rates of all dive operators worldwide. The websites of dive operators were used to collect general operating information and price data for instructional courses, snorkel excursions, single-day dive trips, and multi-day dive trips. Base map was generated using the R package rnaturalearth version 0.1.0[104].

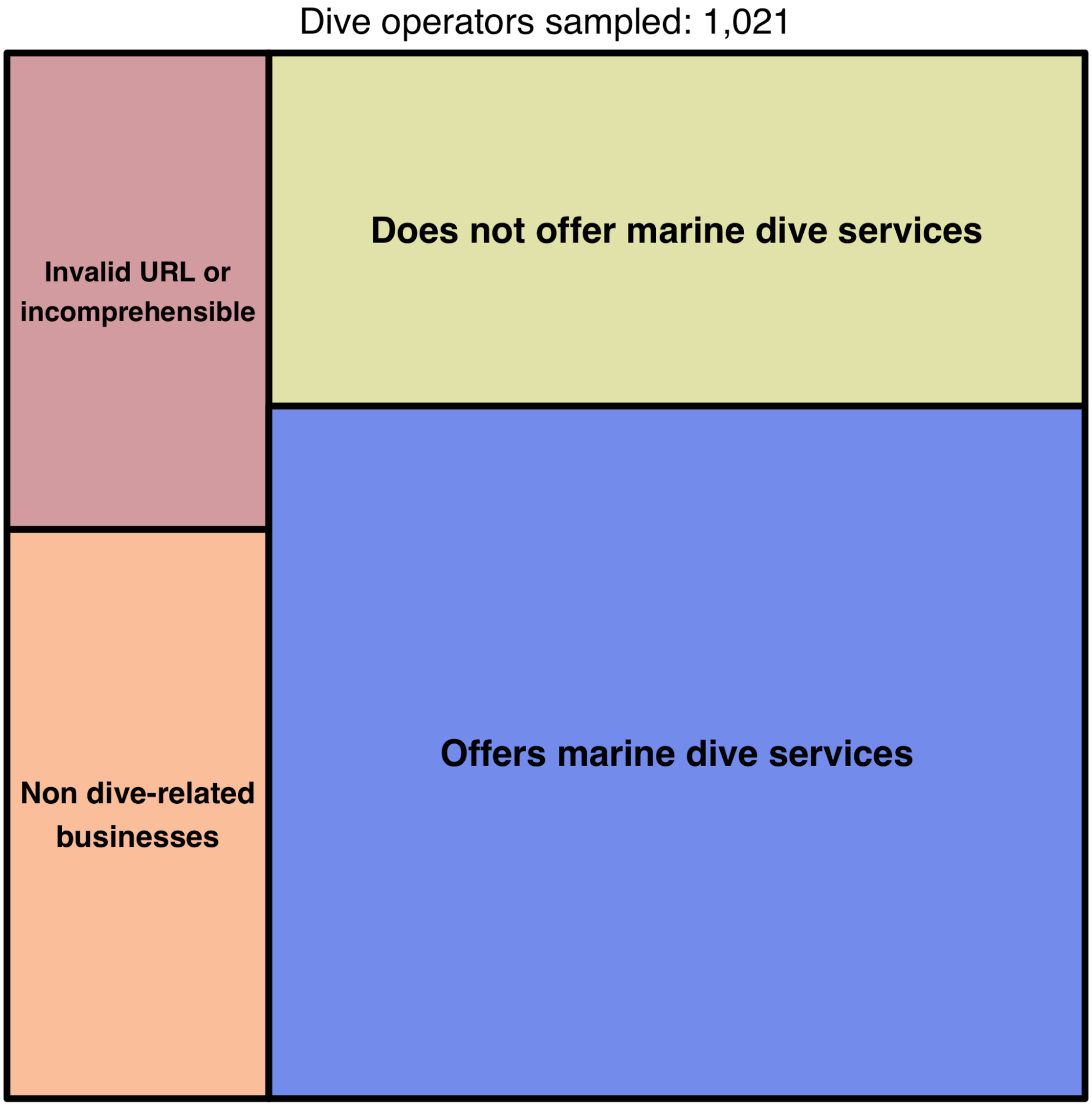

Supplementary Fig. 8 | Validity of sampled dive operators for the purpose of collecting data on prices for marine diving activities.

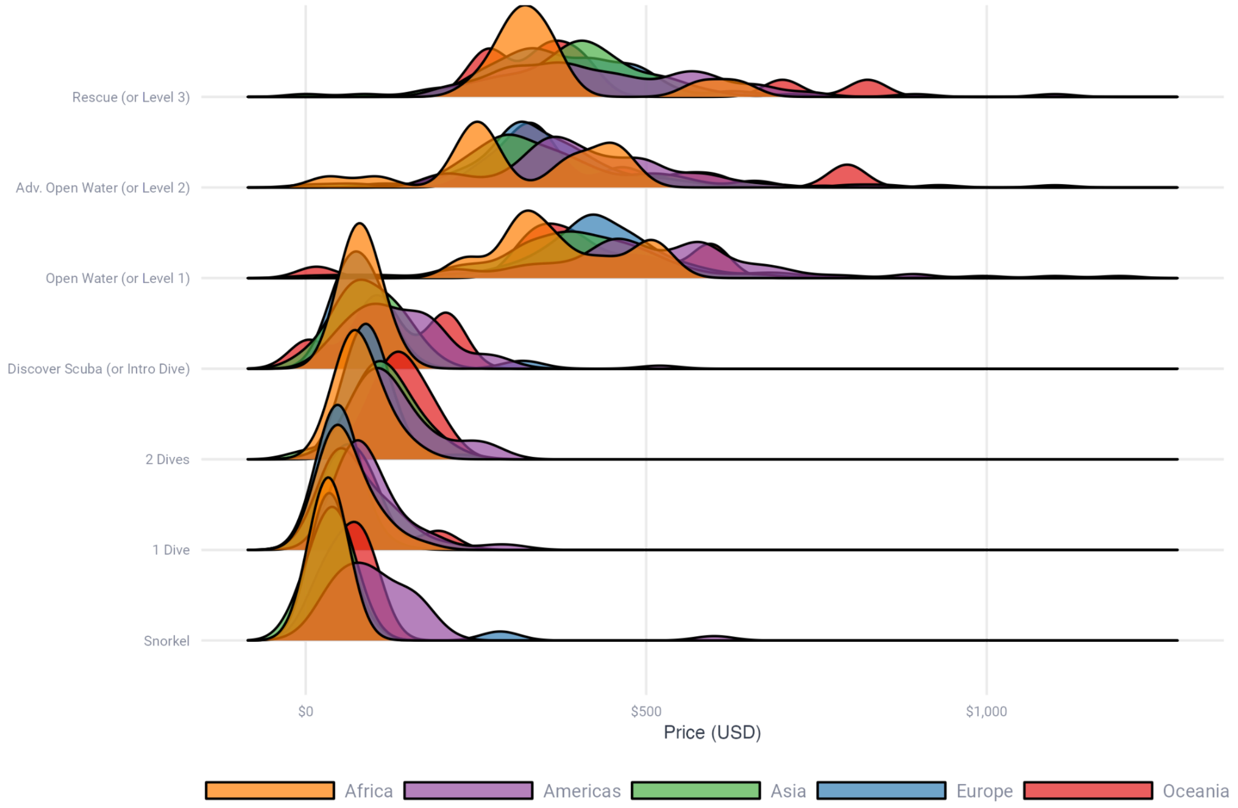

Supplementary Fig. 9 | Distribution of prices collected for single day dive trips and instructional courses by continent.

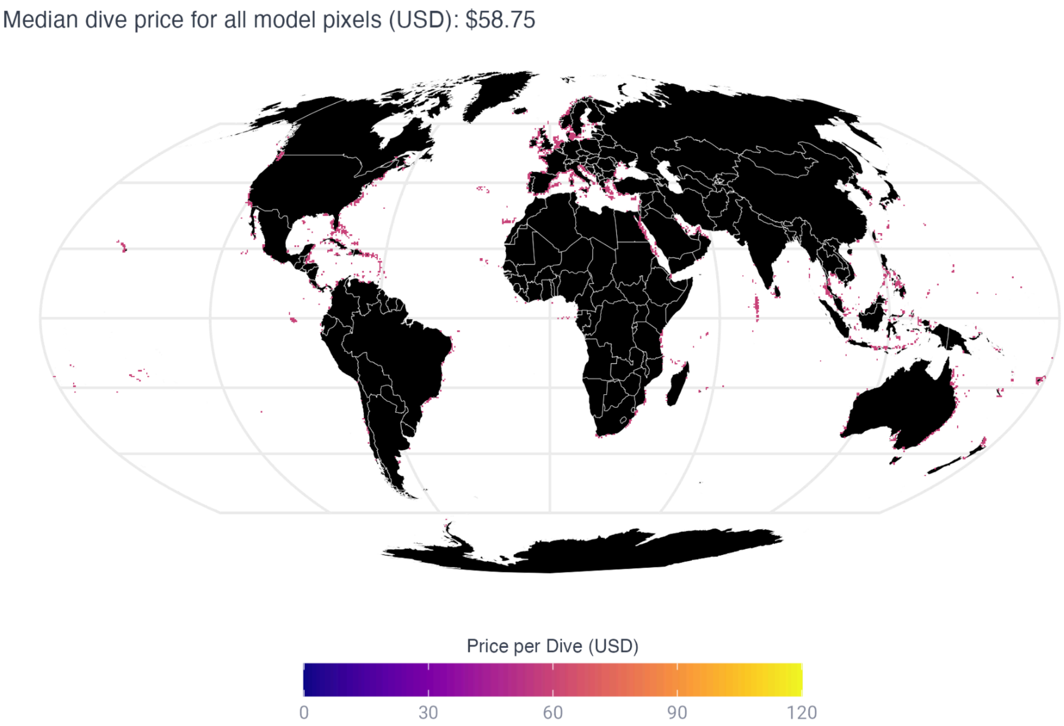

Supplementary Fig. 10 | Model input: Price per dive by ocean pixel. Prices were calculated by uniformly applying the global median dive price to all ocean pixels deemed suitable for diving (reference case). Base map was generated using the R package rnaturalearth version 0.1.0[104].

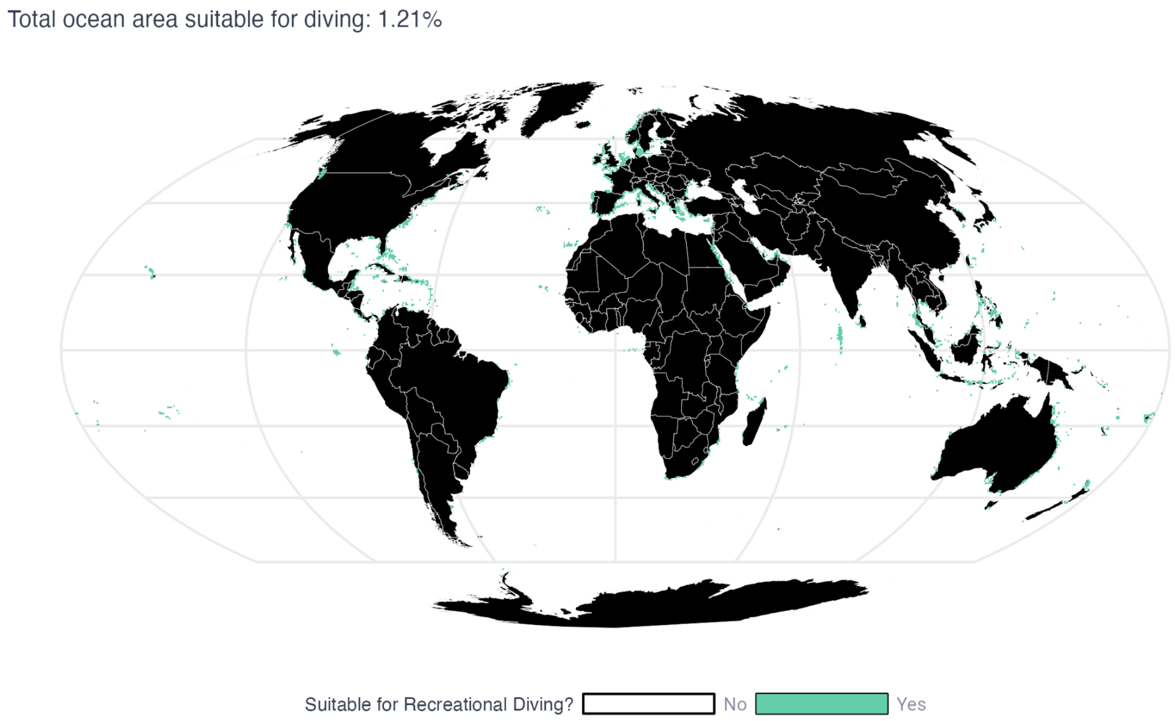

Supplementary Fig. 11 | Model input: Suitability for recreational diving by ocean pixel. Base map was generated using the R package rnaturalearth version 0.1.0[104].

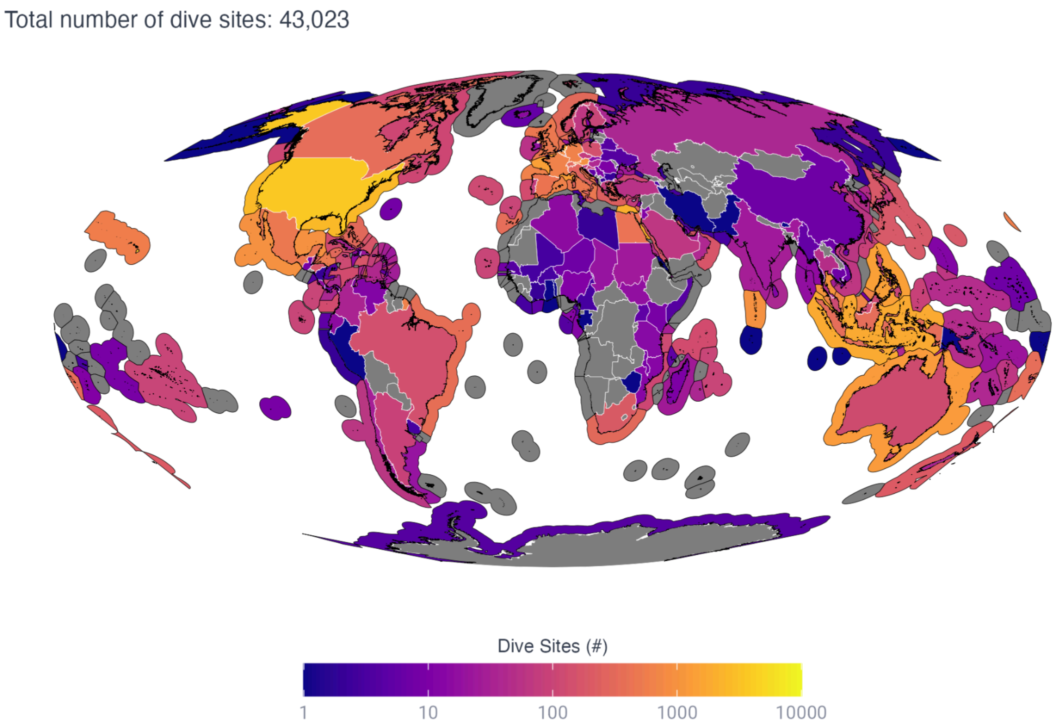

Supplementary Fig. 12 | Number of recreational dive sites by land and EEZ area. Dive sites and their locations are from the crowdsourced Diveboard database. Only sites with at least one valid recreational dive recorded at that location between 2010 and 2020 were included in the final dataset. Base map was generated using the R package rnaturalearth version 0.1.0[104].

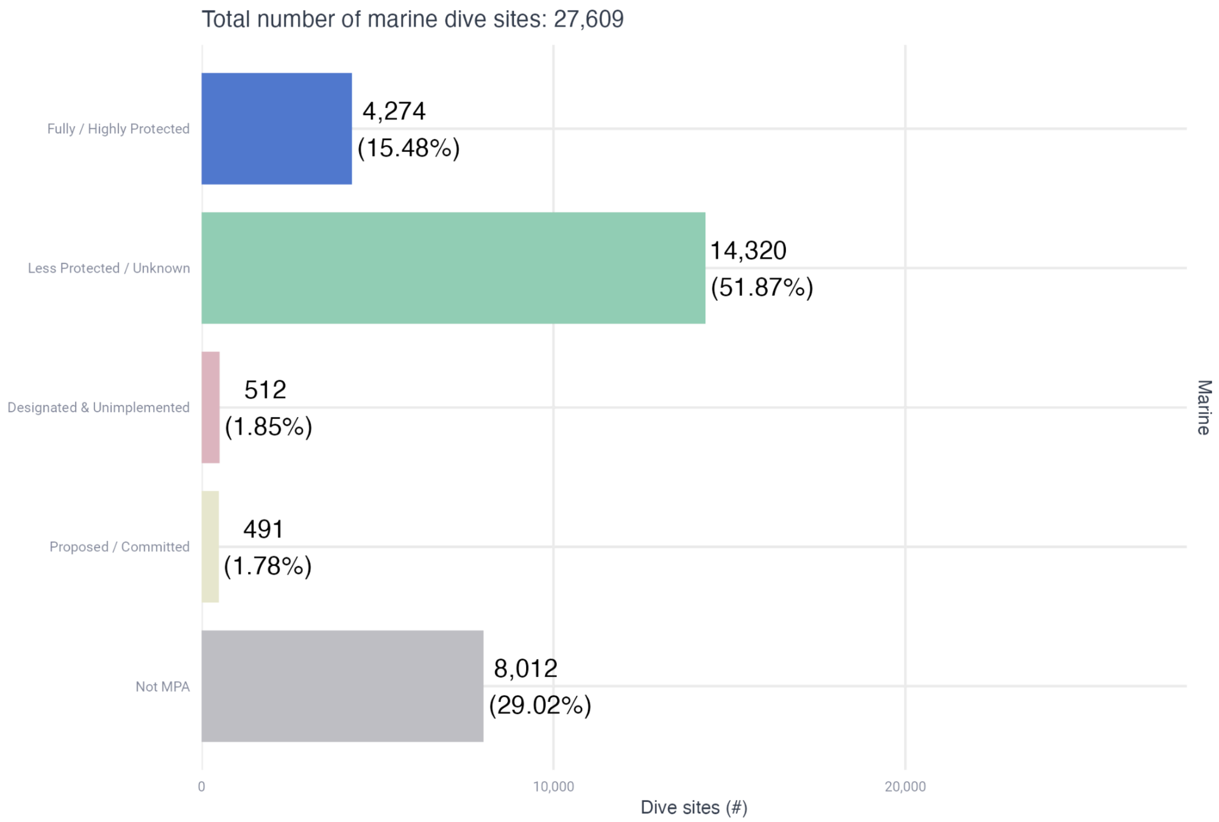

Supplementary Fig. 13 | Protection status of existing marine dive sites. Dive sites and their locations are those considered to pertain to recreational diving from the crowdsourced Diveboard database and protected area boundaries are from the Marine Protection Atlas database. Only sites falling within an ocean area are included.

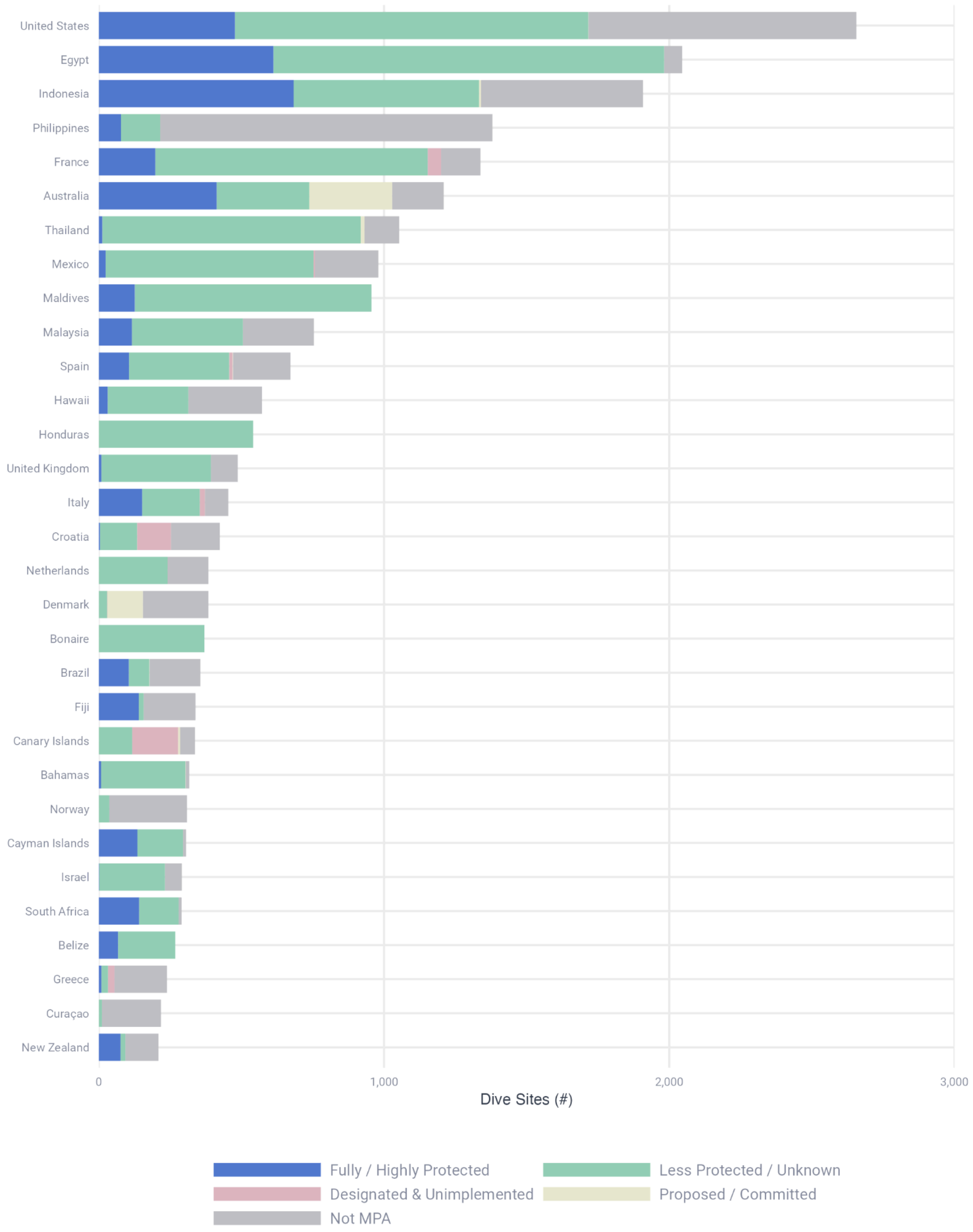

Supplementary Fig. 14 | Protection status of existing marine dive sites by EEZ area. Dive sites and their locations are those considered to pertain to recreational diving from the crowdsourced Diveboard database and protected area boundaries are from the Marine Protection Atlas database. Only sites falling within an ocean area area included.

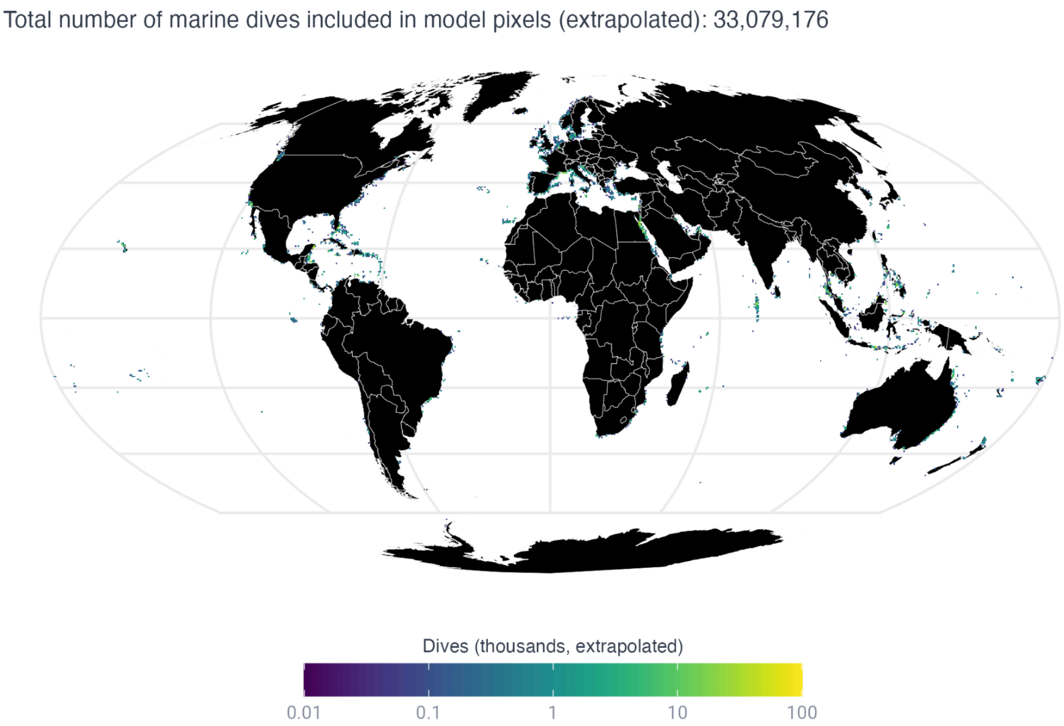

Supplementary Fig. 15 | Model input: Estimated number of scuba dives per ocean pixel per year. Pixels are 50 x 50 km^2. Base map was generated using the R package rnaturalearth version 0.1.0[104].

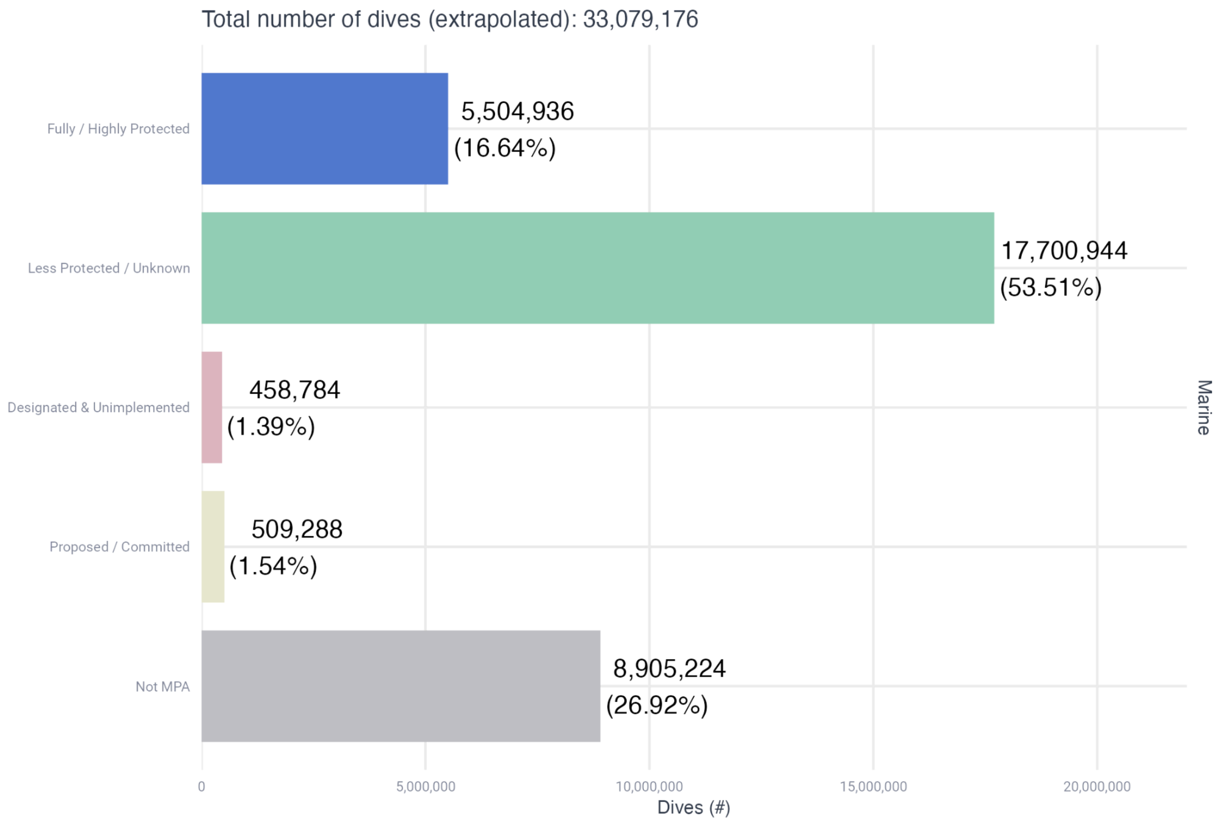

Supplementary Fig. 16 | Number of marine dives made globally by protection status of the corresponding dive site. Dive sites, their locations, and numbers of dives are from the crowdsourced Diveboard database and protected area boundaries are from the MPA Atlas database.

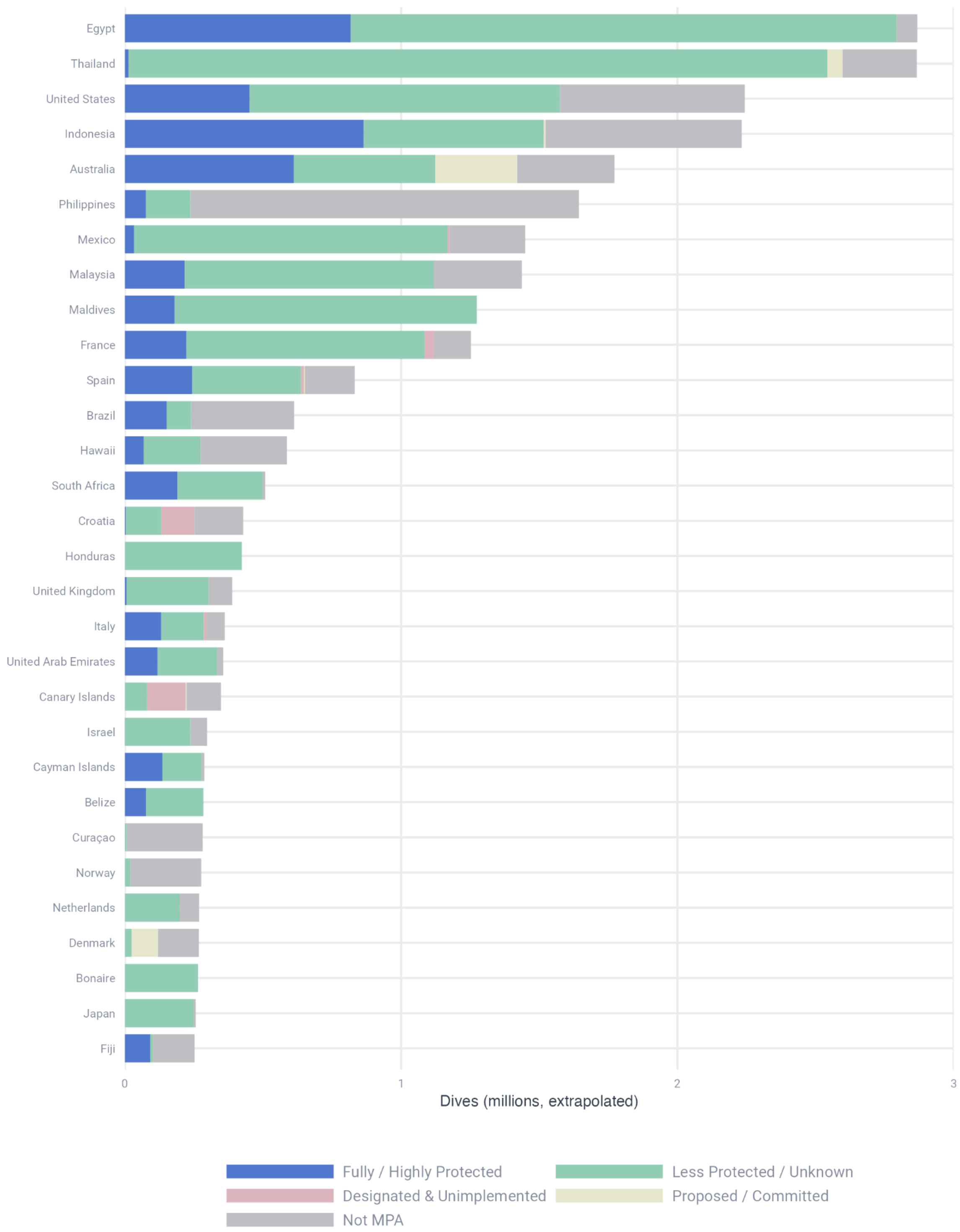

Supplementary Fig. 17 | Number of marine dives made globally by protection status of the corresponding dive site by EEZ area. Dive sites, their locations, and numbers of dives are from the crowdsourced Diveboard database and protected area boundaries are from the MPA Atlas database.

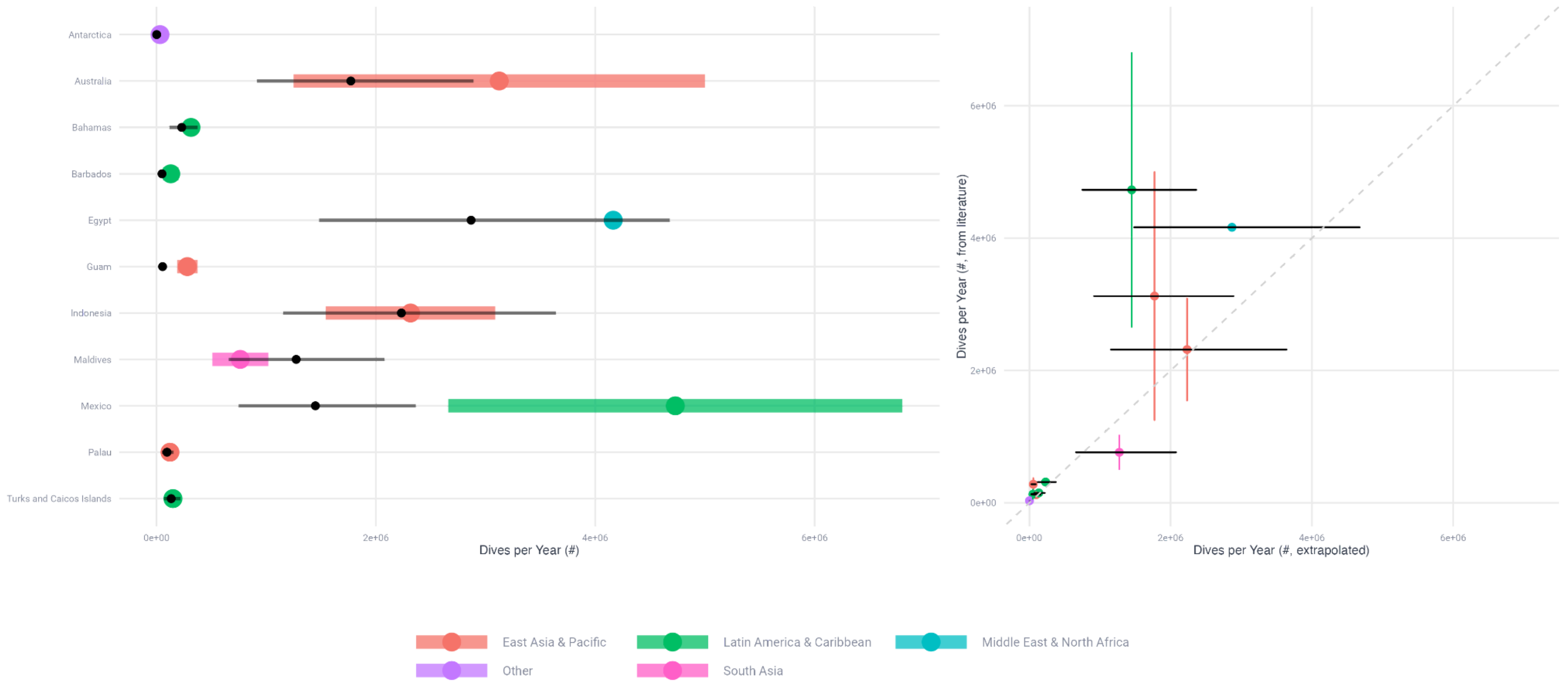

Supplementary Fig. 18 | Annual number of dives made in the EEZs of various countries: Literature-reported versus extrapolated values. In the left panel, colored bars indicate the range in reported dives from literature (where available) and the colored points indicate the median number of dives reported in literature (where available). Black points indicate our best extrapolated estimate for marine diving in that country based on a global total of 50.7 million dives annually and the black bars indicate the range in extrapolated dives considered in our sensitivity analysis. The colors of the bars showing literature-reported values represent the region in which the country's EEZ is located. The right panel shows extrapolated values (x-axis) plotted against literature reported values (y-axis) for each country. The diagonal dashed line indicates a 1:1 relationship. The colored and black bars indicate the ranges in reported dives from literature and that considered in our sensitivity analysis respectively.

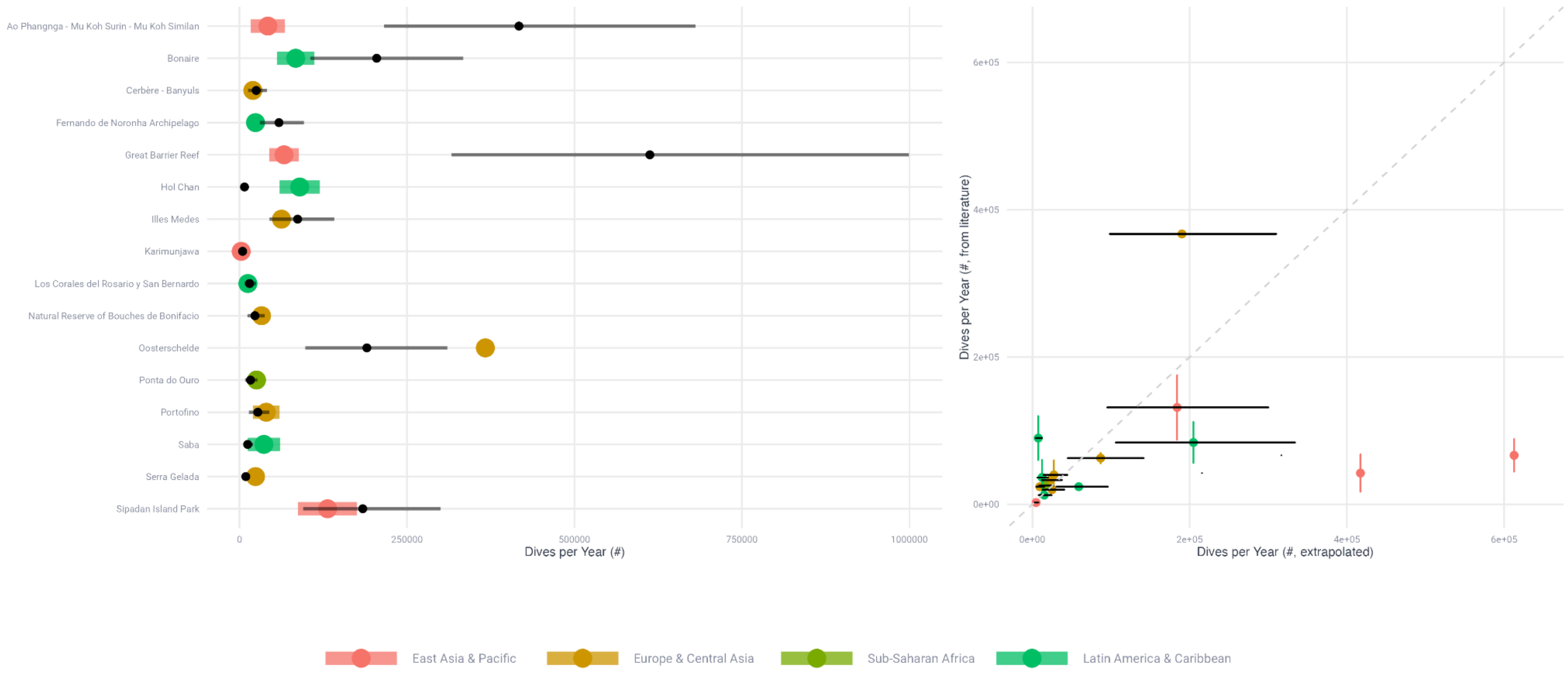

Supplementary Fig. 19 | Annual number of dives made in various Marine Protected Areas: Literature-reported versus extrapolated values. In the left panel, colored bars indicate the range in number of dives reported in literature (where available) and the colored points indicate the median number of dives reported in literature (where available). Black points indicate our best extrapolated estimate for the MPA based on a global total of 50.7 million dives annually and the black bars indicate the range in extrapolated dives considered in our sensitivity analysis. The colors of the bars showing literature-reported values represent the region in which the Marine Protected Area is located. The right panel shows extrapolated values (x-axis) plotted against literature reported values (y-axis) for each MPA. The diagonal dashed line indicates a 1:1 relationship. The colored and black bars indicate the ranges in reported dives from literature and that considered in our sensitivity analysis respectively.

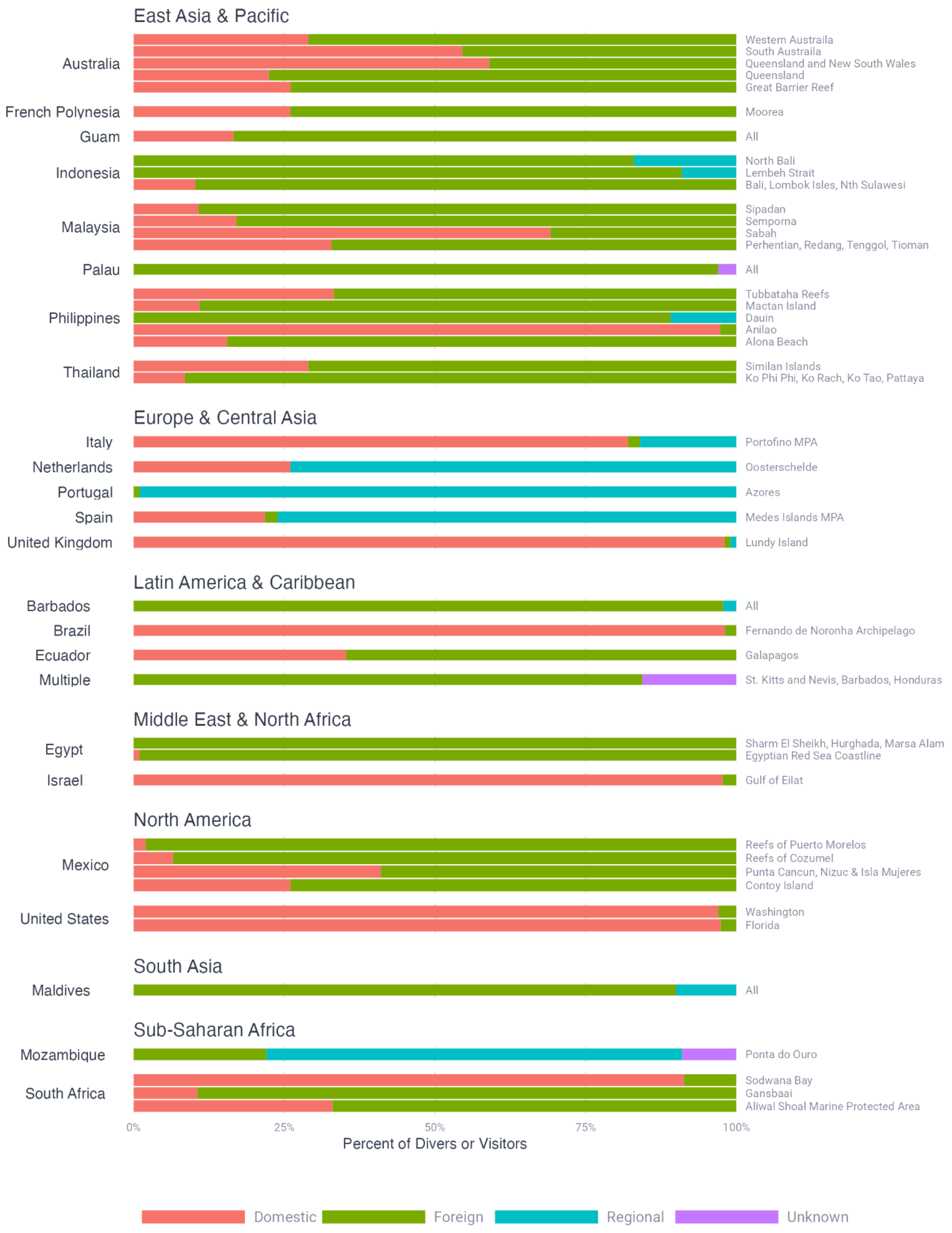

Supplementary Fig. 20 | Origins of divers (or other marine-based tourists) from literature.

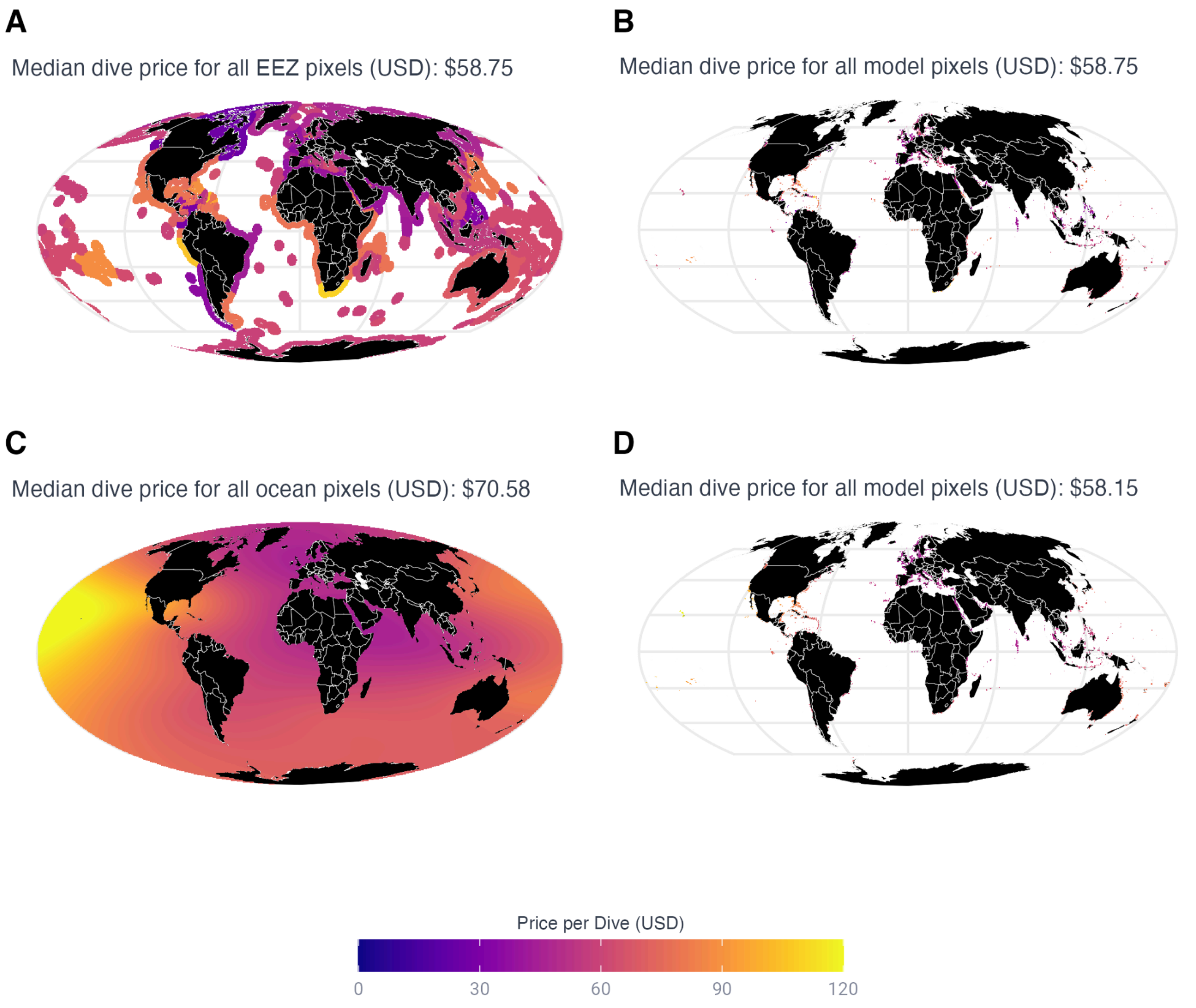

Supplementary Fig. 21 | Sensitivity analysis prices per dive by ocean pixel. Panel A shows the median per dive price calculated for each country applied to the EEZ areas under the jurisdiction of that country. If price data was not available for a country, the median price per dive for the region was applied instead. Panel B shows this for only the ocean pixels deemed suitable for diving (Scenario 3). Panel C shows the median per dive price interpolated spatially across all ocean pixels based on the location of the dive operator. Panel D shows this for only the ocean pixels deemed suitable for diving (Scenario 4). Base maps were generated using the R package rnaturalearth version 0.1.0[104].

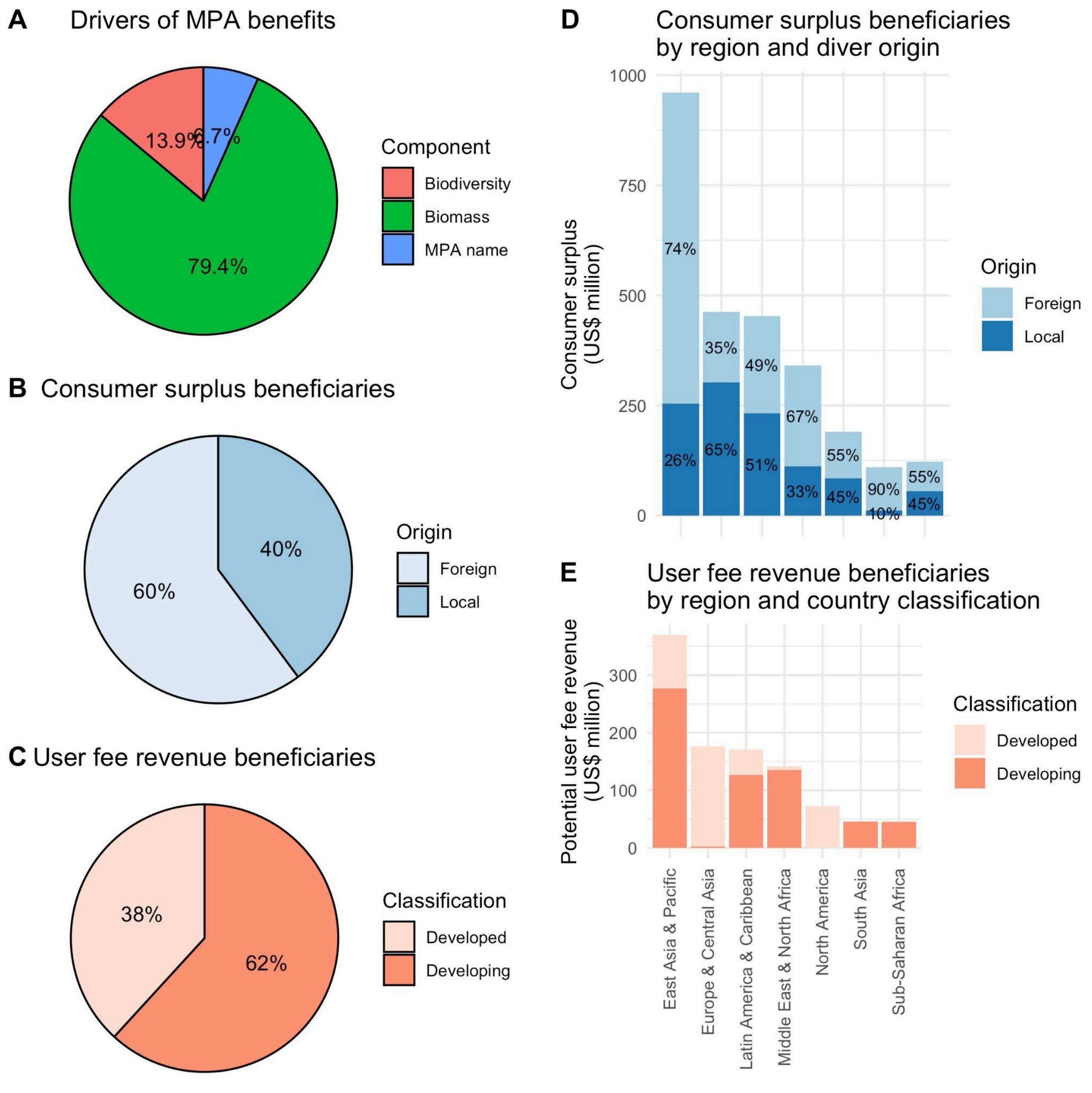

Supplementary Fig. 22 | Drivers and beneficiaries of dive tourism benefits using region attribute to normalize biodiversity score. A) Contribution of different components to dive tourism benefits from MPAs. B) Distribution of consumer surplus benefits associated with MPA-driven dive tourism benefits by diver origin globally. C) Distribution of dive fee revenue associated with dive tourism if a $53 dive fee were to be implemented to capture MPA-driven benefits by country classification globally. D) Distribution of consumer surplus benefits associated with MPA-driven dive tourism benefits by region. E) Distribution of dive fee revenue associated with dive tourism if a $53 dive fee were to be implemented to capture MPA-driven benefits by region. For panels C and E, a user fee of US$53 per dive was used, because it results in no change in the number of dives, dive revenue, and consumer surplus pre- and post-MPA.

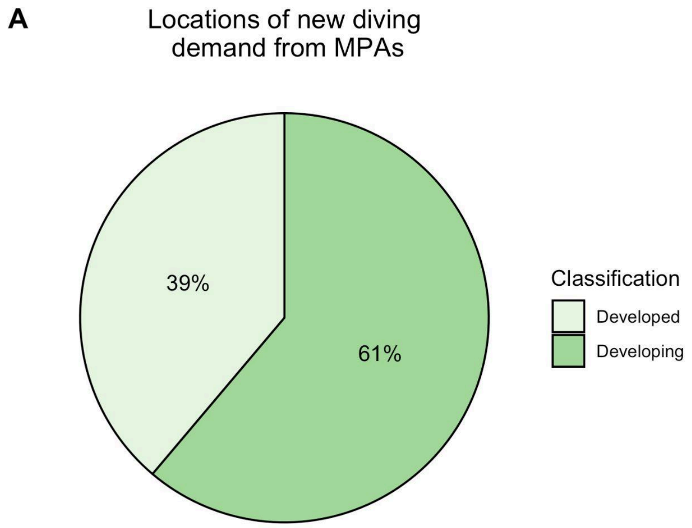

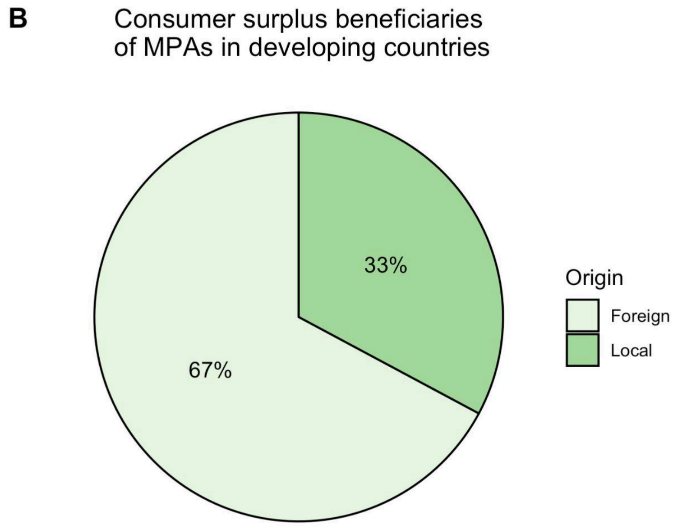

Supplementary Fig. 23 | Location and beneficiaries of new dive demand from MPAs. A) Locations of new diving generated by MPAs. 61% of new diving from protecting all dive sites will be demanded in developing countries. B) Consumer surplus beneficiaries of new MPAs in developing countries. 67% of the consumer surplus from new MPAs is expected to be captured by divers of foreign origin.

Supplementary Tables

Supplementary Table S1 | Glossary of model parameters and variables

Symbol	Meaning
N	number of samples
Δ	symbol for change
i	pixel index (area of each pixel is 50 km x 50 km)
Q_d	number of dives demanded
Q_s	number of dives supplied by the industry
mpa	indicator of MPA designation (“*mpa*” *if* pixel *i* is an MPA and *“0”* if pixel *i* is not an MPA)
P	price per dive
a	horizontal intercept of the demand curve (number of dives that would be demanded when *P = 0*)
b	slope of the demand curve
B	Biomass
ΔB	change in biomass resulting from implementation of an MPA
ΔS	change in species diversity resulting from implementation of an MPA
α	weight used to scale the effect of *ΔB* on the demand curve
β	weight used to scale the effect of *ΔS* on the demand curve
μ	effect of the MPA designation (“MPA name effect”) on the demand curve
x	fish stock index
F	dive fee
t	time index
$s_{i \to i}$	fraction of adult biomass from pixel *i* that remains in pixel *i*
$s_{j \to i}$	fraction of adult biomass from an adjacent pixel (*j*) that moves to pixel *i*
r	population growth rate
K	population carrying capacity
$\rho_{i \to i}$	fraction of viable larvae produced in pixel *i* that settle in pixel *i*
$\rho_{j \to i}$	fraction of viable larvae produced in an adjacent pixel (*j*) that settle in pixel *i*
$d_{j \to i}$	distance between an adjacent pixel (*j*) and pixel i

σ_{larvae}	spread of the larval dispersal kernel
PLD	pelagic larval dispersal
g	feature index
γ	feature weight
w	curvature of a power function analogous to a species–area curve
X	fraction of total habitat

Supplementary Table 2 | Aggregate weight of species per taxonomic class. Values from Sala et al. (2021) correspond to the relative importance of each taxonomic class and area function of the number of species, as well as their extinction risk and functional and evolutionary distinctiveness.

Taxonomic group	Aggregate weight from Sala et al. (2021)	Rank from Sala et al. (2021)	% of all species mentions on dive operator websites	Rank based on dive operator mentions
Elasmobranchii	34.0%	1	18.5%	2
Actinopterygii	27.3%	2	49.1%	1
Anthozoa	16.3%	3	1.1%	9
Mammalia	5.0%	4	4.8%	6
Malacostraca	3.8%	5	8.1%	3
Cephalopoda	2.1%	6	5.0%	5
Bivalvia	1.5%	7	0.6%	10
Holothuroidea	1.0%	8	0.4%	11
Reptilia	0.5%	9	5.9%	4
Gastropoda	0.7%	10	3.8%	7
Asteroidea	< 0.5%	11	1.4%	8
Echinoidea	< 0.5%	12	0.4%	12
Hydrozoa	< 0.5%	13	0.3%	13
Polychaeta	< 0.5%	14	0.2%	14
Porifera	< 0.5%	15	0.1%	16
Tunicata (Ascidiacea / Thaliacea)	< 0.5%	16	0.2%	15

Supplementary Table 3 | Prices for day trips and dive certification courses by continent (N = 1,658).

**Activity**	**Median Price [IQR]** (USD)	**N**
Snorkel	$47 [32,87]	97
1-tank dive	$59 [42,90]	262
2-tank dive	$110 [85,139]	282
Discover Scuba (or intro dive for non-certified divers)	$90 [63,127]	188
Open Water (or level 1 certification)	$430 [362,504]	319
Advanced Open Water (or level 2 certification	$350 [296,437]	277
Rescue (or level 3 certification)	$391 [317,476]	233

Supplementary Table 4 | Prices for SCUBA diving activities reported in scientific literature. Studies are ordered alphabetically by region.

Region	Study	Location(s)	Year	Price per dive (USD)	Price per dive trip* (USD) [± SD]	Price per snorkel trip (USD) [± SD]	Notes
East Asia & Pacific	Clua *et al.*, 2011[31]	Moorea, French Polynesia	2009	$62.50 - $75	$125 - $150	—	Range encompasses local and international prices. Diving with lemon sharks is a major attraction on these dives.
	Pascoe *et al.*, 2014[32]	Indonesia (Bali, Lombok Isles, Nth Sulawesi), Malaysia (Perhentian, Redang, Tenggol, Tioman), and Thailand (Ko Phi Phi, Ko Rach, Ko Tao, and Pattaya)	2010	$505.40†	—	—	International visitors only
				$60.75†	—	—	Domestic visitors only
Europe & Central Asia	Rodrigues *et al.*, 2016[33]	Medes Islands MPA, Spain	2013	~€50 ($66.42‡)	—	—	A single 50 minute dive
Latin America & Caribbean	Arcos-Aguilar *et al.*, 2021[34]	Mexico	2019	$49.10§	$98.20 [± $7.41]	$48.93 [± $24.29]	Mexican Pacific region
				$49.48§	$98.96 [± $90.75]	$20.83 [± $9.38]	Gulf of Mexico region
				$65.75§	$131.50 [± $37.17]	$65.67 [± $29.73]	Northwest Pacific region
				$47.57§	$95.13 [± $31.01]	$45.47 [± $18.62]	Yucatan Peninsula region
	Schuhmann *et al.*, 2013[35]	Barbados	2007 - 2009	$52.79§	$105.58 [± 38.40]	—	Respondents were given ranges and responses were coded using the midpoint of the indicated range (e.g., $75 for a range of $50-100) and responses for the highest range–"more than $200"–were coded as $201)

* Assumed to include two dives unless otherwise noted
† Includes the cost of the dive package, transport costs to the dive site from the diver's home (including any international travel costs), accommodation, and other expenditures
‡ Converted from EUR to USD using the average exchange rate in 2013
§ Calculated by us based on information reported in the study

Supplementary Table 5 | Scuba diving participation by Americans (2015-2020) as reported by the Sports and Fitness Industry Association (SFIA). Source: Adapted from ref. [45].

Year	Scuba Diving Participants (thousands)	"Casual" Divers (participate 1-7 times per year)		"Core" Divers (participate 8+ times per year)	
		**Number** (thousands)	**% of Total**	**Number** (thousands)	**% of Total**
2015	3,274	2,405	73.46%	869	26.54%
2016	3,111	2,292	73.67%	819	26.33%
2017	2,874	2,113	73.52%	761	26.48%
2018	2,849	2,133	74.87%	716	25.13%
2019	2,715	2,016	74.25%	699	25.75%
2020	2,588	1,880	72.64%	708	27.36%

Supplementary Table 6 | National or subnational scuba diving statistics reported in scientific literature. Studies are ordered alphabetically by region.

Region	Study	Location	Year	Divers (per year)	Dives (per year)	Dive sites	Certifications (per year)	Dive operators	Notes
East Asia & Pacific	Clua *et al.*, 2011[31]	Opunohu, French Polynesia	2005 - 2009	15,262	30,524 - 61,048†	—	—	—	Number of divers is the average per year over the range.
	Davis and Tisdell[49]	Queensland, Australia	1991	—	800,000 - 1 million	—	—	—	Higher end estimate includes resort dives.
	De Brauwer *et al.*, 2017[98]	Indonesia and Philippines	n.d.	101,505	203,010 - 406,020†	—	—	—	Number of divers participating in muck diving only.
	Depondt and Green, 2006[10]	Cambodia, Indonesia, Malaysia, Philippines, Singapore, Thailand, Vietnam	2004	107,320	450,000	—	—	417	Number of divers was reported by surveyed operators. Number of dives was estimated based on an average of 3 dives per diver per year by the authors and it was noted that 56% of all dives took place in an MPA.
		French Polynesia	2004	—	—	—	—	42	
	Dimmock and Cummins, 2013[51]	Great Barrier Reef Marine Park, Australia	2003	22,200	44,400 - 88,800†	—	—	—	Number of divers is 5% of all marine tourism visitors to the Great Barrier Reef Marine Park per year.
	Hampton *et al.*, 2018[56]	Sipadan Island Park, Malaysia	n.d.	63,000	87,600†	—	—	—	Number of divers staying in Sabah visiting Sipadan and nearby islands. Number of dives estimated based on daily number of dive permits issued (120) and an assumed 2 dives per permit per day.
	Huveneers *et al.*, 2017[99]	Neptune Islands Marine Park, Australia	2014	10,236	—	—	—	—	Number of divers participating in cage diving tours.
		Queensland and New South Wales, Australia	2014	13,978	—	—	—	—	Number of divers reported by operators offering tours to see gray nurse sharks.

Region	Study	Location	Year	Divers (per year)	Dives (per year)	Dive sites	Certifications (per year)	Dive operators	Notes
		Osprey Reef, Australia		1,848	—	—	—	—	Number of liveaboard divers.
East Asia & Pacific (continued)	Lippmann, 2018[52]	Australia	2010 - 2015	68,728 - 1.3 million†	1.25 - 5 million†	—	—	—	Estimated based on instances of diving fatalities and the average annual number of fatalities.
	Mazaya *et al.*, 2019[54]	Karimunjawa National Park, Indonesia	n.d.	853	1,706 - 3,412†	—	—	—	Number of divers was estimated by dividing the average number of annual visitors (13,252) by their primary intent for visiting
	Mustika *et al.*, 2020[55]	Indonesia	2017	772,171	1,544,342 - 3,088,684†	—	—	235	Number of operators only includes those offering shark SCUBA diving or snorkeling
	Oracion *et al.*, 2005[60]	Mabini, Luzon, Philippines	1994	22,870	45,740 - 91,480†	—	—	—	Number of divers to have visited the Mabini area in a year (the majority were identified as being domestic tourists from Manila).
	Tapsuwan and Asafu-Adjaye, 2008[62]	Mu Koh Similan Marine National Park, Thailand	2003	8,500 - 17,000	17,000 - 68,000†	—	—	—	Number of divers is estimated based on the average number of visitors to the park (34,000) and the knowledge that ~50% are SCUBA divers or snorkelers. Range corresponds to assuming that 25-50% of visitors are divers.
	Teh *et al.*, 2018[58]	Semporna Priority Conservation Area, Malaysia	2013	26,525 - 45,149	106,100 - 180,596†	—	—	—	Number of divers was estimated assuming that 80% of dive arrivals to Sabah visited Semporna each year.
	van Beukering *et al.*, 2007[53]	Guam	2002	61,746	256,000 - 340,000	—	—	—	Number of dives was estimated based on the number of tanks filled per day and the number of divable days per year.

Region	Study	Location	Year	Divers (per year)	Dives (per year)	Dive sites	Certifications (per year)	Dive operators	Notes
					187,492 - 374,984				Number of dives was estimated based on the number of local (~⅓) and foreign (~⅔) divers and the number of dives made per year.
East Asia & Pacific (continued)	Vianna *et al.*, 2012[59]	Palau	2010	40,976	81,952 - 163,904†	—	—	—	Number of divers was estimated based on the percentage of tourists of different nationalities and their participation rates in SCUBA.
	Vianna *et al.*, 2018[57]	Sipadan Island Park, Malaysia	2012	43,900	87,800 - 175,600†	—	—	—	Number of divers visiting the island of Sipadan each year.
	Wilks, 1993[50]	Queensland, Australia	1991	—	> 1 million	—	34,691	—	Certifications include courses of all levels (Open Water to Instructor, including specialties). Number of dives includes both training and leisure dives.
	Wongthong and Harvey, 2014[61]	Koh Tao, Thailand	2009 - 2011	130,000 - 150,000	260,000 - 600,000†	—	> 60,000	43	Number of divers is the number of visitors to the island (dive tourism is the main attraction). Number of dive certifications only includes those issued by PADI and SSI.
Europe & Central Asia	Albayrak *et al.*, 2019[67]	Kemer, Turkey	2017	—	20,000 - 60,000	—	—	20	Lower end estimate only includes dives made by certified divers; higher end estimate includes discovery (resort) dives.
	Cerrano *et al.*, 2017[48]	Medes Islands MPA, Spain	n.d.	—	60,000 - 70,000	—	—	—	
		Portofino MPA, Italy		—	45,000 - 60,000	—	—	—	
		Natural Reserve of Bouches de		—	> 33,000	—	—	—	

Region	Study	Location	Year	Divers (per year)	Dives (per year)	Dive sites	Certifications (per year)	Dive operators	Notes
		Bonifacio, France							
		Serra Gelada Marine Park, Spain		—	> 24,000	—	—	—	
		Cerbere - Banyuls, France		—	20,000	—	—	—	
Europe & Central Asia (continued)	Cerrano *et al.*, 2017[48]	11 MPAs in southern Spain, southern France, and Italy	n.d.	—	235,059	—	—	—	Number of dives corresponds to an average of 7,123 diver trips per year across the 11 MPAs with each having an average of 3 dives per diver trip.
		47 MPAs with diving in the Mediterranean	2012	—	1,000,000	—	—	—	Number of dives is likely greater than this.
	Lucrezi *et al.*, 2017[63]	Portofino MPA, Liguria, Italy	2010 - 2014	—	20,000 - 60,000	21 + partial reserve area	—	—	Number of dives includes dives made both at the 21 established dive sites in the general reserve and in the entirety of the partial reserve.
	Lucrezi *et al.*, 2019[64]	Portofino MPA, Liguria, Italy	2012 - 2017	—	50,000	—	—	—	Number of dives is the annual average based on trends since 2012
	Rodrigues *et al.*, 2016[66]	Medes Islands MPA, Spain	2012	—	55,647	13	—	—	Number of dives per day is capped at 450 in the MPA.
	Rousseau and Tejerizo, 2020[65]	Oosterschelde, Netherlands	2009 - 2010	—	367,100	—	—	—	Note: This is an estuary area (mixed fresh and saltwater). Number of dives was estimated for Dutch and Belgian divers only.
Latin America & Caribbean	Arcos-Aguilar *et al.*, 2021[34]	Mexico	2019	1.33 - 1.70 million	2.66 - 6.80 million†	864	—	264	Number of divers estimated to be serviced by all 264 active operators in Mexico.
	Green and Donnelly, 2003[75]	30 countries‡ in the Wider Caribbean and Pacific coast	2000	3.75 - 7.5 million†	15 million	—	—	—	Number of dives was estimated based on a survey of 22% of dive operators in the region.

	Haas *et al.*, 2017[68]	Bahamas	2014 - 2015	63,191 - 157,344†	314,688	—	—	44§	Number of dives was extrapolated from diver surveys based on dive operator characteristics.
	Hawkins *et al.*, 2007[70]	Hol Chan Marine Reserve, Belize	1990	30,000	60,000 - 120,000†	—	—	—	

**Region**	**Study**	**Location**	**Year**	**Divers** (per year)	**Dives** (per year)	**Dive sites**	**Certifications** (per year)	**Dive operators**	**Notes**
Latin America & Caribbean (continued)	Hawkins *et al.*, 2007[70]	Saba Marine Park, Netherlands Antilles	1993 - 2002	—	12,460 - 60,564	28	—	—	Minimum and maximum number of dives were estimated assuming an average of 224 - 2,163 dives per site per year. All dive sites are buoyed, divers cannot enter from shore, and only one boat is allowed on any buoy at one time.
	Parsons and Thur, 2008[71]	Bonaire National Marine Park, Bonaire	2001	28,000	56,000 - 112,000†	—	—	—	Number of divers was estimated based on the number of tags purchased that year ($10 USD each). All divers are required to display a valid tag to dive in the MPA.
	Pires *et al.*, 2016[72]	Fernando de Noronha Archipelago, Brazil	n.d.	—	24,000	—	—	—	Number of dives is the number of “diving operations” referenced in the text which we assumed to be dives rather than divers.
	Rudd, 2001[74]	Turks and Caicos Islands	n.d.	—	150,000	—	—	—	Number of dives estimated across all dive sites.
	Schuhmann *et al.*, 2008[69]	Barbados	2007 - 2008	30,000 - 50,000	60,000 - 200,000†	—	—	—	Number of divers estimated to visit Barbados annually.
	Trujillo *et al.*, 2017[73]	Los Corales del Rosario y San Bernardo, Colombia	n.d.	4,200	8,400 - 16,800†	—	—	—	
Middle East & North Africa	Cesar, 2003[76]	Egypt	2000	566,108	4,164,171	—	—	—	Estimates include both foreign and domestic divers and cover both the Red Sea and Gulf of Aqaba.

	Hasler and Ott, 2008[100]	Dahab, South Sinai, Egypt (Red Sea)	n.d.	—	> 30,000	4			Number of dives made on four frequently dived sites (all accessible from shore only).
					< 300	4			Number of dives made on four infrequently dived sites (all accessible from shore only)

Region	Study	Location	Year	Divers (per year)	Dives (per year)	Dive sites	Certifications (per year)	Dive operators	Notes
North America	Buzzacott *et al.*, 2018[77]	United States	2015	3,624,000	25,992,577	—	—	—	Values were estimated based on annual participation rates determined by the Sports and Fitness Industry Association (SFIA) survey adjusted for the total population size of the United States.
	Ladd *et al.*, 2002[81]	British Columbia, Canada	1999	—	125,392[¶]	—	—	76 - 84	Number of dives was estimated based on the number of tank fills reported by all dive shops and charter operators in the province. Number of operators only includes those with tank-filling stations.
	Northern Economics, Inc., 2016[80]	Washington, United States	2014	9,750	2,188,000	—	—	40	Number of divers is the number of active divers residing in the state that dive locally, regionally, or internationally. Total number of certified divers in the state was reported to be 65,000. Number of dives is the “resident diving participant days in public waters” adjusted assuming 2 dives per participant day.
	Oh *et al.*, 2008[78]	United States	2004	2.8 million	23 - 46 million[#]	—	—	—	It is unclear whether the reported number of dives is limited to those made in the United States or those made by American divers anywhere in the world.
	Pendleton and Rooke, 2006[79]	California, United States	2000	870,000	1,380,000	—	—	—	Number of divers is the number of participants in any coastal diving activity.

Region	Study	Location	Year	Divers (per year)	Dives (per year)	Dive sites	Certifications (per year)	Dive operators	Notes
South Asia	Zimmerhackel *et al.*, 2018[82]	Maldives	2016	77,168	509,308 - 1,018,617†	—	—	—	Number of divers is the number of dive visits per year. Number of dives was estimated assuming the reported average of 6.6 dive days per tourist and 1-2 dives per dive day. Note: both statistics pertain to shark diving only.

Region	Study	Location	Year	Divers (per year)	Dives (per year)	Dive sites	Certifications (per year)	Dive operators	Notes
Sub-Saharan Africa	Celliers and Schleyer, 2008[83]	Sodwana Bay, South Africa	1996	120,000	240,000 - 480,000†	—	—	—	Number of divers corresponds to the peak in the number of visiting divers that ultimately resulted in implementation of sustainable diving limits to mitigate damages.
	Depondt and Green, 2006[10]	Madagascar	2004	—	—	—	—	22	
		Mauritius		—	—	—	—	32	
		Mayotte		—	—	—	—	5	
		Réunion		—	—	—	—	16	
	Dicken, 2014[85]	Sodwana Bay, South Africa	2011 - 2012	15,295 - 16,277	59,553	—	—	—	Range of the number of divers is the 95% confidence interval.
	Dicken and Hosking, 2008[101]	Aliwal Shoal Marine Protected Area, South Africa	2007	1,065	2,133	—	—	—	Note: Both statistics only encompass tiger shark diving in the MPA.
	Lucrezi *et al.*, 2017[63]	Ponta do Ouro Partial Marine Reserve in Mozambique	2010 - 2014	—	21,000 - 30,000$^{\Delta}$	20 reefs	—	—	Prior to implementation of the reserve in 2009, it was estimated that up to 62,000 dives were made in the area each year.
	Lucrezi *et al.*, 2019[64]	Ponta do Ouro Partial Marine Reserve in Mozambique	2012 - 2017	—	30,000$^{\Delta}$	—	—	—	

Region	Study	Location	Year	Divers (per year)	Dives (per year)	Dive sites	Certifications (per year)	Dive operators	Notes
	Mabaleka *et al.*, 2020[102]	Gansbaai, South Africa	2019	—	—	—	—	8	Number of licensed shark cage diving operators.
	Saayman and Saayman, 2014[84]	Sodwana Bay, South Africa	2012	60,000 - 80,000	120,000 - 320,000†	—	—	—	Number of divers is the approximate annual number of visiting divers since 2005.
Sub-Saharan Africa (continued)	Schoeman *et al.*, 2016[86]	Sodwana Bay, South Africa	n.d.	15,780	59,553	—	—	—	
Other & Multiple	Depondt and Green, 2006[10]	5 Francophone countries and territories of the Indian and Pacific Oceans	2004	69,150	270,000	—	—	119	Number of divers was reported by surveyed operators. Number of dives was estimated based on an average of 3 dives per diver per year by the authors and it was noted that 5% of all dives took place in an MPA but reserves in these areas are not adequately protected.
	Healy *et al.*, 2020[103]	20 countries with shark diving	n.d.	590,000	—	—	—	—	Number of divers is the number of tourists participating in elasmobranch viewing activities across 20 different countries. Note: May include non-SCUBA activities.
	Lamers and Gelter, 2011[87]	Antarctica	2008 - 2009	10,000 - 11,000	20,000 - 44,000†	—	—	—	

†Our estimate based on values reported in the study. Unless otherwise noted, this estimate was made assuming the average diver makes 2-4 dives per year per location.

‡Anguilla, Antigua and Barbuda, Bahamas, Barbados, Belize, Bermuda, British Virgin Islands, Cayman Islands, Colombia, Costa Rica, Dominica, Dominican Republic, El Salvador, Grenada, Guadeloupe, Honduras, Jamaica, Martinique, Mexico, Montserrat, Netherlands Antilles, Panama, Puerto Rico, St. Barthelemy, St. Lucia, St. Vincent & the Grenadines, Trinidad and Tobago, Turks and Caicos, US Virgin Islands, Venezuela.

§Includes shore-based operators, domestic based live-aboard vessels, and foreign live-aboard vessels (all based in Florida).

¶The study encompassed a 14-month period so this value was proportionally adjusted to represent a 12 month period.

#The value reported in the study was the number of days of scuba participation. Range is our extrapolation based on an average of either 1 or 2 dives made per day of participation.

ΔNumber of dives is not representative of the entire reserve area, only the number of dives made from the primary launch site of Ponta do Ouro.

Supplementary Table 7 | Divers' willingness to pay (WTP) to avoid fishing or fishing gear encounters or to dive in a marine protected area (MPA) where fishing is prohibited. Studies are ordered alphabetically by first author.

Study	Value	Frequency / units and year	Marginal WTP (US$)	Base value (US$)	% change in WTP	N	Method and year	Location
Arin and Kramer, 2002[94]	Entrance fee to a hypothetical MPA in the Philippines where fishing is prohibited	Daily, in addition to the other costs of the trip, 1997	$3.70	—	—		Choice experiment (exploratory contingent valuation method)	Anilao, Batangas, Philippines
			$5.50					Mactan, Cebu, Philippines
			$3.40					Panglao, Bohol, Philippines
			$4.20					***Average across all locations***
Gill *et al.*, 2015[93]	Completely avoid encounters with fishing or fishing gear	Per two-tank dive, 2011 - 2012	$91.31	$110	83.01%	184	Choice experiment	St. Kitts and Nevis
			$67.99	$60	113.32%	162		Honduras
			$114.04	$110	103.67%	110		Barbados
			$103.20	***$93.96***	***109.83%***	***505***		***Pooled data for all locations***
Tongson and Dygico, 2004[95]	Entrance fee to the Tubbataha MPA in the Philippines where fishing is prohibited	Daily*, in addition to the other costs of the trip, 1999	$13.70	—	—		Choice experiment	Tubbataha MPA, Philippines

* Study reported WTP for a multi-day trip. Value was adjusted to reflect daily WTP assuming an average trip duration of 3 diving days.

Supplementary Table 8 | Divers' willingness to pay (WTP) to see a greater number of fish, more fish biomass, or a greater proportion of large fish while diving. Studies are ordered alphabetically by first author.

Study	Value	Frequency / units and year	Marginal WTP (US$)	Base value (US$)	% change in WTP	N	Method and year	Location
Gill *et al.*, 2015[93]	Fish abundance: median ($17/m^2$) to many ($25/m^2$)	Per two-tank dive, 2011 - 2012	$118.41	$110	107.65%	184	Choice experiment	St. Kitts and Nevis
			$52.89	$60	88.15%	162		Honduras
			$139.63	$110	126.94%	110		Barbados
			$83.64	***$93.96***	***89.02%***	***505***		***Pooled data for all locations***
	Proportion of large fish: few (1-10%) to most (> 50%)	Per two-tank dive, 2011 2012	$122.01	$110	110.92%	184	Choice experiment	St. Kitts and Nevis
			$39.28	$60	65.47%	162		Honduras
			$72.69	$110	66.08%	110		Barbados
			$65.99	***$93.96***	***70.23%***	***505***		***Pooled data for all locations***
Grafeld *et al.*, 2016[13]	Biomass: low (< 25 g/m^2) to high (> 60 g/m^2)	Per dive, 2013	$21.82	$100	21.82%	180	Choice experiment	Guam

Supplementary Table 9 | Divers' willingness to pay (WTP) to see a greater number of individuals of specific species (or species types) while diving. Studies are ordered alphabetically by first author.

Study	Value	Frequency / units and year	Marginal WTP (US$)	Base value(s) (US$)	% change in WTP	N	Method	Location
Cazabon-Mannette *et al.*, 2017[29]	Sea turtles: 0 to 3+ encounters	Per two-tank dive, 2007 - 1010	$111.97	$50	223.94%		Choice experiment (conditional logit model)	Tobago
			$108.68		217.36%		Choice experiment (mixed logit model)	
			$243.43		486.86%		Choice experiment (latent class logit model, class 1)	
			$41.99		83.98%		Choice experiment (latent class logit model, class 2)	
			$126.52		**253.04%**		***Average across all methods***	
Grafeld *et al.*, 2016[13]	Sharks & sea turtles: none to sharks and turtles both present	Per dive, 2013	$35.14	$100	35.14%		Discrete choice experiment	Guam
	Napoleon wrasse: few (1/m^2) to many (4+/m^2)	Per dive, 2013	$16.30	$100	16.30%		Discrete choice experiment	Guam
Schuhmann *et al.*, 2013[35]	Sea turtles: 0 to 3+ encounters	Per two-tank dive, 2007 - 2009	$97.77	$75	130.36%		Choice experiment (conditional logit model)	Barbados
			$100.86		134.48%		Choice experiment (mixed logit model)	
			$148.59		198.12%		Choice experiment (latent class logit model, class 1)	
			$56.16		74.88%		Choice experiment (latent class logit model, class 2)	
			$100.85		**134.47%**		***Average across all methods***	

Study	Value	Frequency / units and year	Marginal WTP (US$)	Base value(s) (US$)	% change in WTP	N	Method	Location
Shideler and Pierce, 2016[28]	Sharks: 0 to 5 encounters	Per two-tank dive, 2015	$219.38	$55	398.87%		Choice experiment (non-Florida divers only)	Florida, USA
			$128.94		234.44%		Choice experiment (Florida divers only)	
			$145.72		***264.95%***		***Pooled data for all divers***	
	Goliath groupers: 0 to 40 encounters	Per two-tank dive, 2015	$336.03	$55	610.96%		Choice experiment (non-Florida divers only)	Florida, USA
			$167.97		305.40%		Choice experiment (Florida divers only)	
			$202.37		***367.95%***		***Pooled data for all divers***	

Supplementary Table 10 | Divers' willingness to pay (WTP) to see a greater number of different species while diving. Studies are ordered alphabetically by first author.

Study	Value	Frequency / units and year	Marginal WTP (US$)	Base value (US$)	% change in WTP	N	Method	Location
Cazabon-Mannette *et al.*, 2017[29]	Low (≤ 5 fish species) to high (≥ 25 fish species)	Per two-tank dive, 2007 - 2010	$40.45	$50	80.90%		Choice experiment (conditional logit model)	Tobago
			$29.21		58.42%		Choice experiment (mixed logit model)	
			$38.61		77.22%		Choice experiment (latent class logit model, class 2)	
			$36.09		***72.18%***		***Average across all methods***	
Grafeld *et al.*, 2016[13]	Low (2 fish species/m^2) to high (8 fish species/m^2)	Per dive, 2013	$23.43	$100	23.43%		Discrete choice experiment	Guam
Schuhmann *et al.*, 2013[35]	Low (≤ 5 fish species) to high (≥ 25 fish species)	Per two-tank dive, 2007 - 2009	$109.65	$75	146.20%		Choice experiment (conditional logit model)	Barbados
			$101.58		135.44%		Choice experiment (mixed logit model)	
			$187.75		250.33%		Choice experiment (latent class logit model, class 1)	
			$50.89		67.85%		Choice experiment (latent class logit model, class 2)	
			$112.47		***149.96%***		***Average across all methods***	

Supplementary Table 11 | Effect of congestion on divers' willingness to pay (WTP). Studies are ordered alphabetically by first author.

Study	Value	Frequency / units	WTP (US$)	Base value(s) (US$)	% change in WTP	Method	Location and year
Cazabon-Mannette *et al.*, 2017[29]	Change in number of other divers at site: -5 (15 to 10)	Per two-tank dive	$0	$50	**0%**	Choice experiment, conditional logit result	Tobago, 2007 - 2010
	Change in number of other divers at site: -10 (15 to 5)	Per two-tank dive	$41.20	$50	**82.40%**	Choice experiment, conditional logit result	Tobago, 2007 - 2010
	Change in number of other divers at site: -15 (15 to 0)	Per two-tank dive	$62.80	$50	**125.60%**	Choice experiment, conditional logit result	Tobago, 2007 - 2010
Schuhmann *et al.*, 2013[35]	Change in number of other divers at site: -5 (15 to 10)	Per two-tank dive	$51.50	$75	**68.67%**	Choice experiment, conditional logit result	Barbados, 2007 - 2009
	Change in number of other divers at site: -10 (15 to 5)	Per two-tank dive	$74.92	$75	**99.89%**	Choice experiment, conditional logit result	Barbados, 2007 - 2009
	Change in number of other divers at site: -15 (15 to 0)	Per two-tank dive	$101.23	$75	**134.97%**	Choice experiment, conditional logit result	Barbados, 2007 - 2009
Shideler and Pierce, 2016[28]	Change in number of divers on charter: 4 (4 to 8)	Per two-tank dive	$0	$55	**0%**	Choice experiment	Florida, 2015
	Change in number of divers on charter: 11 (4 to 15)	Per two-tank dive	-$52.55	$55	**-95.55%**	Choice experiment	Florida, 2015
	Change in number of divers on charter: 21 (4 to 25)	Per two-tank dive	-149.12	$55	**-271.13%**	Choice experiment	Florida, 2015

Supplementary Table 12 | Cost estimates for managing an additional 1% of the global ocean area in MPAs.

Study	Reported MPA Management Costs	Estimated Cost to Manage an Additional 1% of the Global Ocean Area in an MPA
Balmford *et al*., 2004[96]	US$5.4 billion - US$12.5 billion per year for managing 20% of the global ocean area in MPAs	US$0.27 billion - US$0.625 billion
Cullis-Suzuki and Pauly, 2010[97]	US$25 billion per year for managing 20% of the global ocean area in MPAs	US$1.25 billion

Supplementary Table 13 | Dive revenue estimates from base and sensitivity model scenarios.

Scenario	Maximum possible revenue from combining dive fee and MPAs	Difference from the base scenario
0. Base scenario	$2.30 billion	—
1. Lower bound on the global number of dives	$1.19 billion	-$1.11 billion
2. Upper bound on the global number of dives	$3.76 billion	+$1.46 billion
3. Mean dive price per region instead of globally constant price	$2.30 billion	$0
4. Spatially extrapolated dive price instead of globally constant price	$2.27 billion	-$0.03 billion
5. Normalize biodiversity score by sovereignty category	$2.23 billion	-$0.07 billion
6. Normalize biodiversity score by region	$1.88 billion	-0.42 billion

Supplementary Table 14 | Diver origins by region calculated from literature studies.

Region	Local (%)	Foreign (%)
East Asia & Pacific	26.46	73.54
Europe & Central Asia	65.35	34.65
Latin America & Caribbean	51.18	48.82
Middle East & North Africa	32.82	67.18
North America	44.58	55.42
South Asia	10.00	90.00
Sub-Saharan Africa	44.97	55.03

Supplementary References

1. Marine Conservation Institute. Atlas of Marine Protection. http://mpatlas.org (2020).
2. Sala, E. *et al.* A General Business Model for Marine Reserves. *PLOS ONE* **8**, e58799 (2013).
3. Viana, D. F., Halpern, B. S. & Gaines, S. D. Accounting for tourism benefits in marine reserve design. *PLOS ONE* **12**, e0190187 (2017).
4. Sala, E. *et al.* Protecting the global ocean for biodiversity, food and climate. *Nature* **592**, 397–402 (2021).
5. Varian, H. R. *Intermediate Microeconomics: A Modern Approach*. (W. W. Norton & Company, 2009).
6. Whitelaw, P. A., King, B. E. M. & Tolkach, D. Protected areas, conservation and tourism – financing the sustainable dream. *J. Sustain. Tour.* **22**, 584–603 (2014).
7. Spergel, B. Raising Revenues for Protected Areas, A Menu of Options. (2001).
8. Fuest, C. & Kolmar, M. A theory of user-fee competition. *J. Public Econ.* **91**, 497–509 (2007).
9. Witt, B. Tourists' Willingness to Pay Increased Entrance Fees at Mexican Protected Areas: A Multi-Site Contingent Valuation Study. *Sustainability* **11**, 3041 (2019).
10. Depondt, F. & Green, E. Diving user fees and the financial sustainability of marine protected areas: Opportunities and impediments. *Ocean Coast. Manag.* **49**, 188–202 (2006).
11. Dharmaratne, G. S., Yee Sang, F. & Walling, L. J. Tourism potentials for financing protected areas. *Ann. Tour. Res.* **27**, 590–610 (2000).
12. Arin, T. & Kramer, R. A. Divers' willingness to pay to visit marine sanctuaries: an exploratory study. *Ocean Coast. Manag.* **45**, 171–183 (2002).
13. Grafeld, S. *et al.* Divers' willingness to pay for improved coral reef conditions in Guam: An untapped source of funding for management and conservation? *Ecol. Econ.* **128**, 202–213 (2016).
14. Sala, E. *et al.* A General Business Model for Marine Reserves. *PLOS ONE* **8**, e58799 (2013).
15. Kaschner, K. *et al.* AquaMaps: Predicted range maps for aquatic species. World wide web electronic publication, www.aquamaps.org version 08/2016c. www.aquamaps.org (2016).
16. FAO. *The State of World Fisheries and Aquaculture: Meeting the sustainable development goals*. (FAO, 2018).
17. RAM Legacy Stock Assessment Database. Version 4.44-assessment-only. Released 2018-12-22. (2018).
18. May, R. M. Islands biogeography and the design of wildlife preserves. *Nature* **254**, 177–178 (1975).
19. Halpern, B. S. *et al.* Recent pace of change in human impact on the world's ocean. *Sci. Rep.* **9**, 11609 (2019).
20. Kuhn, M. & Wickham, H. Tidymodels: A collection of packages for modeling and machine learning using tidyverse principles. (2020).
21. Wright, M. N. & Ziegler, A. ranger: A Fast Implementation of Random Forests for High Dimensional Data in C++ and R. *J. Stat. Softw.* **77**, 1–17 (2017).
22. Pauly, D. & Froese, R. FishBase. World Wide Web electronic publication. (2022).
23. Siegel, D. A., Kinlan, B. P., Gaylord, B. & Gaines, S. D. Lagrangian descriptions of marine larval dispersion. *Mar. Ecol. Prog. Ser.* **260**, 83–96 (2003).

24. Hijmans, R. J. raster: Geographic Data Analysis and Modeling. (2021).
25. Marshall, D. J., Krug, P. J., Kupriyanova, E. K., Byrne, M. & Emlet, R. B. The Biogeography of Marine Invertebrate Life Histories. *Annu. Rev. Ecol. Evol. Syst.* **43**, 97–114 (2012).
26. Ramesh, N., Rising, J. A. & Oremus, K. L. The small world of global marine fisheries: The cross-boundary consequences of larval dispersal. *Science* **364**, 1192–1196 (2019).
27. Fontoura, L. *et al.* Protecting connectivity promotes successful biodiversity and fisheries conservation. *Science* **375**, 336–340 (2022).
28. Shideler, G. S. & Pierce, B. Recreational diver willingness to pay for goliath grouper encounters during the months of their spawning aggregation off eastern Florida, USA. *Ocean Coast. Manag.* **129**, 36–43 (2016).
29. Cazabon-Mannette, M., Schuhmann, P. W., Hailey, A. & Horrocks, J. Estimates of the non-market value of sea turtles in Tobago using stated preference techniques. *J. Environ. Manage.* **192**, 281–291 (2017).
30. Schuhbauer, A. *et al.* Global economic impact of scuba dive tourism. *Nat. Commun.* (in review).
31. Clua, E. *et al.* Business partner or simple catch? The economic value of the sicklefin lemon shark in French Polynesia. *Mar. Freshw. Res.* **62**, 764–770 (2011).
32. Pascoe, S. *et al.* Estimating the potential impact of entry fees for marine parks on dive tourism in South East Asia. *Mar. Policy* **47**, 147–152 (2014).
33. Rodrigues, L. C., van den Bergh, J. C. J. M., Loureiro, M. L., Nunes, P. A. L. D. & Rossi, S. The Cost of Mediterranean Sea Warming and Acidification: A Choice Experiment Among Scuba Divers at Medes Islands, Spain. *Environ. Resour. Econ.* **63**, 289–311 (2016).
34. Arcos-Aguilar, R. *et al.* Diving tourism in Mexico – Economic and conservation importance. *Mar. Policy* **126**, 104410 (2021).
35. Schuhmann, P. W., Casey, J. F., Horrocks, J. A. & Oxenford, H. A. Recreational SCUBA divers' willingness to pay for marine biodiversity in Barbados. *J. Environ. Manage.* **121**, 29–36 (2013).
36. Evans, C. What's the Difference Between Technical Diving and Recreational Diving? https://blog.padi.com/whats-difference-technical-diving-recreational-diving/ (2017).
37. Torres, P., Bolhão, N., Tristão da Cunha, R., Vieira, J. A. C. & Rodrigues, A. dos S. Dead or alive: The growing importance of shark diving in the Mid-Atlantic region. *J. Nat. Conserv.* **36**, 20–28 (2017).
38. Agardy, M. T. Accommodating ecotourism in multiple use planning of coastal and marine protected areas. *Ocean Coast. Manag.* **20**, 219–239 (1993).
39. Spalding, M. *et al.* Mapping the global value and distribution of coral reef tourism. *Mar. Policy* **82**, 104–113 (2017).
40. Blamey, L. K. & Bolton, J. J. The economic value of South African kelp forests and temperate reefs: Past, present and future. *J. Mar. Syst.* **188**, 172–181 (2018).
41. South, A. rnaturalearth: World Map Data from Natural Earth. R package version 0.1.0. (2017).
42. Flanders Marine Institute. Maritime Boundaries Geodatabase: Maritime Boundaries and Exclusive Economic Zones (200NM), version 11. (2019).
43. Marine Conservation Institute. MPAtlas database. (2021).
44. DEMA. *2022 Diving Fast Facts: Fast Facts on Recreational Scuba Diving and Snorkeling*. 8 (2022).
45. SFIA. *2021 Sports, Fitness, and Leisure Activities Topline Participation Report*. (2021).
46. Kieran, D. The Size of the Scuba Diving Industry. *Medium* (2021).
47. Garrod, B. & Gössling, S. *New frontiers in marine tourism : diving experiences,*

management and sustainability. (Elsevier, 2008).
48. Cerrano, C., Milanese, M. & Ponti, M. Diving for science - science for diving: volunteer scuba divers support science and conservation in the Mediterranean Sea. *Aquat. Conserv. Mar. Freshw. Ecosyst.* **27**, 303–323 (2017).
49. Davis, D. & Tisdell, C. Recreational scuba-diving and carrying capacity in marine protected areas. *Ocean Coast. Manag.* **26**, 19–40 (1995).
50. Wilks, J. Calculating diver numbers: critical information for scuba safety and marketing programs. *S. Pac. Underw. Med. Soc. J.* **23**, 11–14 (1993).
51. Dimmock, K. & Cummins, T. History of scuba diving tourism. in *Scuba Diving Tourism* (Routledge, 2013).
52. Lippmann, J. Analysis of Scuba Diving-Related Fatalities in Australia. (Deakin University, 2018).
53. van Beukering, P. *et al. The economic value of Guam's coral reefs*. (2007).
54. Mazaya, A. F. A., Yulianda, F., & Taryono. Economic valuation of coral reef ecosystem for marine tourismin Karimunjawa National Park. *IOP Conf. Ser. Earth Environ. Sci.* **241**, 012025 (2019).
55. Mustika, P. L. K., Ichsan, M. & Booth, H. The Economic Value of Shark and Ray Tourism in Indonesia and Its Role in Delivering Conservation Outcomes. *Front. Mar. Sci.* **7**, (2020).
56. Hampton, M. P., Jeyacheya, J. & Lee, D. The political economy of dive tourism: precarity at the periphery in Malaysia. *Tour. Geogr.* **20**, 107–126 (2018).
57. Vianna, G. M. S. *et al.* Shark-diving tourism as a financing mechanism for shark conservation strategies in Malaysia. *Mar. Policy* **94**, 220–226 (2018).
58. Teh, L. S. L., Teh, L. C. L. & Jolis, G. An economic approach to marine megafauna conservation in the coral triangle: Marine turtles in Sabah, Malaysia. *Mar. Policy* **89**, 1–10 (2018).
59. Vianna, G. M. S., Meekan, M. G., Pannell, D. J., Marsh, S. P. & Meeuwig, J. J. Socio-economic value and community benefits from shark-diving tourism in Palau: A sustainable use of reef shark populations. *Biol. Conserv.* **145**, 267–277 (2012).
60. Oracion, E. G., Miller, M. L. & Christie, P. Marine protected areas for whom? Fisheries, tourism, and solidarity in a Philippine community. *Ocean Coast. Manag.* **48**, 393–410 (2005).
61. Wongthong, P. & Harvey, N. Integrated coastal management and sustainable tourism: A case study of the reef-based SCUBA dive industry from Thailand. *Ocean Coast. Manag.* **95**, 138–146 (2014).
62. Tapsuwan, S. & Asafu-Adjaye, J. Estimating the Economic Benefit of SCUBA Diving in the Similan Islands, Thailand. *Coast. Manag.* **36**, 431–442 (2008).
63. Lucrezi, S. *et al.* Scuba diving tourism systems and sustainability: Perceptions by the scuba diving industry in two Marine Protected Areas. *Tour. Manag.* **59**, 385–403 (2017).
64. Lucrezi, S., Milanese, M., Cerrano, C. & Palma, M. The influence of scuba diving experience on divers' perceptions, and its implications for managing diving destinations. *PLOS ONE* **14**, e0219306 (2019).
65. Rousseau, S. & Tejerizo Fuertes, A. Country borders and the value of scuba diving in an estuary. *Ocean Coast. Manag.* **184**, 105002 (2020).
66. Rodrigues, L. C., van den Bergh, J. C. J. M., Loureiro, M. L., Nunes, P. A. L. D. & Rossi, S. The Cost of Mediterranean Sea Warming and Acidification: A Choice Experiment Among Scuba Divers at Medes Islands, Spain. *Environ. Resour. Econ.* **63**, 289–311 (2016).
67. Albayrak, T., Caber, M. & Cater, C. Mass tourism underwater: a segmentation approach to motivations of scuba diving holiday tourists. *Tour. Geogr.* **23**, 985–1000 (2021).

68. Haas, A. R., Fedler, T. & Brooks, E. J. The contemporary economic value of elasmobranchs in The Bahamas: Reaping the rewards of 25years of stewardship and conservation. *Biol. Conserv.* **207**, 55–63 (2017).
69. Schuhmann, P., Casey, J. & Oxenford, H. A. The value of coral quality to SCUBA divers in Barbados. in *Proceedings of the 11th International Coral Reef Symposium* (2008).
70. Hawkins, J. P., Roberts, C. M., Kooistra, D., Buchan, K. & White, S. Sustainability of Scuba Diving Tourism on Coral Reefs of Saba. *Coast. Manag.* **33**, 373–387 (2005).
71. Parsons, G. R. & Thur, S. M. Valuing Changes in the Quality of Coral Reef Ecosystems: A Stated Preference Study of SCUBA Diving in the Bonaire National Marine Park. *Environ. Resour. Econ.* **40**, 593–608 (2008).
72. Pires, N. M., Garla, R. C. & Carvalho, A. R. The economic role of sharks in a major ecotourism archipelago in the western South Atlantic. *Mar. Policy* **72**, 31–39 (2016).
73. Trujillo, J. C., Navas, E. J. & Vargas, D. M. Valuing Coral Reef Preservation in a Caribbean Marine Protected Area. Economic Impact of Scuba Diving in Corals of Rosario and San Bernardo National Natural Park, Colombia. *Cuad. Desarro. Rural* **14**, 1–14 (2017).
74. Rudd, M. A. The non-extractive economic value of spiny lobster, Panulirus argus, in the Turks and Caicos Islands. *Environ. Conserv.* **28**, 226–234 (2001).
75. Green, E. & Donnelly, R. Recreational Scuba Diving In Caribbean Marine Protected Areas: Do The Users Pay? *AMBIO J. Hum. Environ.* **32**, 140–144 (2003).
76. Cesar, H. *Economic Valuation of the Egyptian Red Sea Coral Reef*. (2003).
77. Buzzacott, P., Schiller, D., Crain, J. & Denoble, P. J. Epidemiology of morbidity and mortality in US and Canadian recreational scuba diving. *Public Health* **155**, 62–68 (2018).
78. Oh, C.-O., Ditton, R. B. & Stoll, J. R. The Economic Value of Scuba-Diving Use of Natural and Artificial Reef Habitats. *Soc. Nat. Resour.* **21**, 455–468 (2008).
79. Pendleton, L. H. & Rooke, J. *Understanding the Potential Economic Impact of SCUBA Diving and Snorkeling: California*. https://dfg.ca.gov/mlpa/pdfs/binder3diii.pdf (2006).
80. Northern Economics, Inc. *Economic Impacts of Washington State Resident Scuba Divers*. https://static1.squarespace.com/static/5b071ddea2772cebc1662831/t/5b29b045758d466e7e7d813c/1529458773071/SCUBA-Economic-Valuation-Final-report.pdf (2016).
81. Ladd, G., Stepan, V. & Stevens, L. The Abacus Project: establishing the risk of recreational scuba death and decompression illness. *S. Pac. Underw. Med. Soc. J.* **32**, (2002).
82. Zimmerhackel, J. S. *et al.* How shark conservation in the Maldives affects demand for dive tourism. *Tour. Manag.* **69**, 263–271 (2018).
83. Celliers, L. & Schleyer, M. H. Coral community structure and risk assessment of high-latitude reefs at Sodwana Bay, South Africa. *Biodivers. Conserv.* **17**, 3097–3117 (2008).
84. Saayman, M. & Saayman, A. How Deep are Scuba Divers' Pockets? *Tour. Econ.* **20**, 813–829 (2014).
85. Dicken, M. Socio-economic aspects of the Sodwana Bay SCUBA diving industry, with a specific focus on sharks. *Afr. J. Mar. Sci.* **36**, 39–47 (2014).
86. Schoeman, K., Merwe, P. V. der & Slabbert, E. The Perceived Value of a Scuba Diving Experience. *J. Coast. Res.* **32**, 1071–1080 (2016).
87. Lamers, M. & Gelter, H. Diversification of Antarctic tourism: the case of a scuba diving expedition. *Polar Rec.* **48**, 280–290 (2012).
88. Zimmerhackel, J. S. *et al.* How shark conservation in the Maldives affects demand for dive tourism. *Tour. Manag.* **69**, 263–271 (2018).
89. Rudd, M. A. & Tupper, M. H. The Impact of Nassau Grouper Size and Abundance on Scuba Diver Site Selection and MPA Economics. *Coast. Manag.* **30**, 133–151 (2002).

90. Williams, I. D. & Polunin, N. V. C. Differences between protected and unprotected reefs of the western Caribbean in attributes preferred by dive tourists. *Environ. Conserv.* **27**, 382–391 (2000).
91. Thur, S. M. User fees as sustainable financing mechanisms for marine protected areas: An application to the Bonaire National Marine Park. *Mar. Policy* **34**, 63–69 (2010).
92. Peters, H. & Hawkins, J. P. Access to marine parks: A comparative study in willingness to pay. *Ocean Coast. Manag.* **52**, 219–228 (2009).
93. Gill, D. A., Schuhmann, P. W. & Oxenford, H. A. Recreational diver preferences for reef fish attributes: Economic implications of future change. *Ecol. Econ.* **111**, 48–57 (2015).
94. Arin, T. & Kramer, R. A. Divers' willingness to pay to visit marine sanctuaries: an exploratory study. *Ocean Coast. Manag.* **45**, 171–183 (2002).
95. Tongson, E. & Dygico, M. User Fee System for Marine Ecotourism: The Tubbataha Reef Experience. *Coast. Manag.* **32**, 17–23 (2004).
96. Balmford, A., Gravestock, P., Hockley, N., McClean, C. J. & Roberts, C. M. The worldwide costs of marine protected areas. *Proc. Natl. Acad. Sci.* **101**, 9694–9697 (2004).
97. Cullis-Suzuki, S. & Pauly, D. Marine Protected Area Costs as "Beneficial" Fisheries Subsidies: A Global Evaluation. *Coast. Manag.* **38**, 113–121 (2010).
98. De Brauwer, M. *et al.* The economic contribution of the muck dive industry to tourism in Southeast Asia. *Mar. Policy* **83**, 92–99 (2017).
99. Huveneers, C. *et al.* The economic value of shark-diving tourism in Australia. *Rev. Fish Biol. Fish.* **27**, 665–680 (2017).
100. Hasler, H. & Ott, J. A. Diving down the reefs? Intensive diving tourism threatens the reefs of the northern Red Sea. *Mar. Pollut. Bull.* **56**, 1788–1794 (2008).
101. Dicken, M. L. & Hosking, S. G. Socio-economic aspects of the tiger shark diving industry within the Aliwal Shoal Marine Protected Area, South Africa. *Afr. J. Mar. Sci.* **31**, 227–232 (2009).
102. Mabaleka, N., Ntloko, N. J. & Swart, K. Profiles of tourists participating in shark cage diving in Gansbaai, South Africa. *J. Transdiscipl. Res. South. Afr.* **16**, (2020).
103. Healy, T. J., Hill, N. J., Barnett, A. & Chin, A. A global review of elasmobranch tourism activities, management and risk. *Mar. Policy* **118**, 103964 (2020).
104. South, A. rnaturalearth: world map data from natural earth. The R Foundation. https://cran.r-project.org/web/packages/rnaturalearthdata/index.html. (2017).
